# Structure-Activity Relationships of Baicalein and its Analogs as Novel TSLP Inhibitors

**DOI:** 10.1038/s41598-019-44853-5

**Published:** 2019-06-19

**Authors:** Bernie Byunghoon Park, Jae Wan Choi, Dawon Park, Doyoung Choi, Jiwon Paek, Hyun Jung Kim, Se-Young Son, Ameeq Ul Mushtaq, Hyeji Shin, Sang Hoon Kim, Yuanyuan Zhou, Taehyeong Lim, Ji Young Park, Ji-Young Baek, Kyul Kim, Hongmok Kwon, Sang-Hyun Son, Ka Young Chung, Hyun-Ja Jeong, Hyung-Min Kim, Yong Woo Jung, Kiho Lee, Ki Yong Lee, Youngjoo Byun, Young Ho Jeon

**Affiliations:** 1 College of Pharmacy, Korea University, 2511 Sejong-ro, Sejong, 30019 Republic of Korea; 2 Korea Brain Research Institute (KBRI), 61 Cheomdan-ro, Dong-gu, Daegu, 41062 Republic of Korea; 3 School of Pharmacy, Sungkyunkwan University, 2066 Seoburo, Jangan-gu, Suwon 16419 Republic of Korea; 4 Department of Food Science & Technology, Hoseo University, 20 Hoseo-ro 79 beon-gil, Baebang-eup, Asan, Chungcheongnam-do 31499 Republic of Korea; 5 Department of Pharmacology, College of Korean Medicine, Kyung Hee University, 26 Kyungheedae-ro, Dongdaemun-gu, Seoul 02447 Republic of Korea; 6 Biomedical Research Center, Korea University Guro Hospital, 148 Gurodong-ro, Guro-gu, Seoul 08308 Republic of Korea

**Keywords:** Screening, Structure-based drug design

## Abstract

Thymic stromal lymphopoietin (TSLP) plays an important role in the differentiation and proliferation of Th2 cells, resulting in eosinophilic inflammation and numerous allergic diseases. Baicalein (**1**), a major component of *Scutellaria baicalensis*, was found to be the first small molecule to block TSLP signaling pathways. It inhibited effectively eosinophil infiltration in house dust mite-induced and ovalbumin-challenged mouse models. Structure-activity relationship studies identified compound **11a**, a biphenyl flavanone analog, as a novel human TSLP inhibitor for the discovery and development of new anti-allergic drugs.

## Introduction

The prevalence of allergic diseases such as asthma, atopic dermatitis and allergic rhinitis has been steadily increasing over the past few decades^1^, severely affecting the quality of life of millions of people worldwide. Allergic diseases are characterized by abnormal inflammatory responses to allergens due to an imbalance in T helper 1 (Th1) and Th2 cell responses and overproduction of Th2 cells^2,3^. Th2-mediated inflammatory responses, triggered by activated dendritic cells (DCs), play a key role in the pathogenesis and maintenance of allergic diseases^4^. In response to tissue damage or exposure to various environmental allergens, epithelial cells release cytokines such as thymic stromal lymphopoietin (TSLP), interleukin-33 (IL-33) and IL-25^5,6^. These cytokines activate DCs, which in turn stimulate the differentiation of naïve T cells into Th2 subset and secretion of IL-4 from T cells in lymphoid organs, leading to the production of immunoglobulin E (IgE) by B cells^4,7^. Moreover, these cytokines activate group 2 innate lymphoid cells (ILC2 cells)^8,9^, which release IL-13 for mucus secretion, as well as IL-9 and IL-5 for the recruitment of mast cells and eosinophils, respectively. These cytokines orchestrate allergic inflammation, resulting in the development of allergic diseases such as asthma and atopic dermatitis^7^.

Among these cytokines, TSLP, an IL-7-like cytokine, is recognized as a major pro-allergic cytokine in DC-mediated Th2 immune responses^10^. TSLP, primarily produced by epithelial cells, binds to its cognate receptor TSLPR (or cytokine receptor-like factor 2)^11^ and IL-7Rα to initiate the signal transducer and activator of transcription 5 (STAT5) signaling pathway^12^, resulting in the development of Th2 cells and activation of type 2 immune cells^7,13^. Modulation of TSLP/TSLPR signaling by a soluble TSLPR fragment fused to the Fc fragment of immunoglobulin (TSLPR-Ig)^14^ or an anti-TSLP antibody^15^ reduced eosinophilic airway inflammation and allergen-induced bronchoconstriction, alleviating the severity of allergic diseases. These data suggest that blocking TSLP signaling is a promising strategy for the control of allergic diseases. However, despite the pressing need for the discovery of anti-allergic drugs, there have been few reports on small molecule inhibitors targeting the TSLP signaling pathways^16,17^.

Recent studies in cytokine signaling have uncovered the critical role of human TSLP (hTSLP) in allergic responses. As an effort to identify novel low molecular weight (M.W) inhibitors of hTSLP signaling, we screened extracts of an in-house natural product library and identified an active component that binds selectively to hTSLP to inhibit its interaction with the human TSLPR (hTSLPR). Based on the chemical structure of the active component, structure-activity relationship (SAR) studies were performed to identify novel small molecules that inhibit hTSLP/hTSLPR-mediated signaling pathways.

## Results and Discussion

### Identification of compound 1 as an inhibitor of hTSLP-hTSLPR interaction

**Figure 1:**
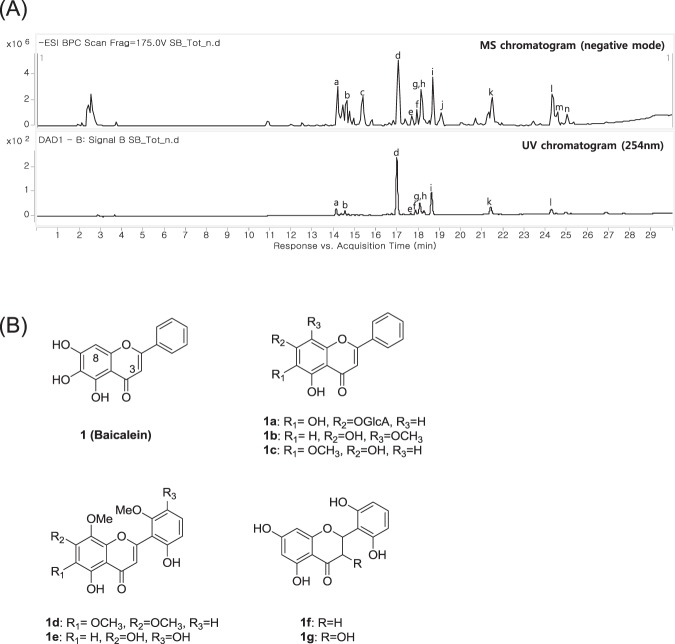
(**A**) MS and UV chromatograms of *S. baicalensis* extracts. (**B**) Structures of compounds isolated from *S. baicalensis*.

Using enzyme-linked immunosorbent assay (ELISA) and STAT5 assay, we screened in-house natural products including *Scutellaria baicalensis*, *Schisandra chinensis, Magnolia officinalis*, and *Persicaria tinctoria*. Among these natural products, *S. baicalensis* and *S. chinensis* displayed weak hTSLP inhibitory activities in the *in vitro* STAT5 assay. The crude extracts of these two plants were subjected to assay-guided isolation over silica gel or C18 semi-preparative HPLC to identify hTSLP-inhibitory components. We analyzed the major peaks of *S. baicalensis* extract by using liquid chromatography-quadruple time-of-flight mass spectrometry (LC-QTOF-MS). The structures of major peaks were assigned based on the UV spectrum, molecular weight, tandem MS fragmentation, and previously reported data (Fig. 1A and Table 1). Among 14 major peaks, 8 were isolated in the pure form and their chemical structures were confirmed (Fig. 1B). Inhibitory activities of eight compounds (**1** and **1a–1g**) against hTSLP/hTSLPR interaction were determined using an ELISA (Table 2). Compound **1** showed the highest hTLSP inhibition among the investigated compounds showing >50% inhibition at 0.3 mM. However, the other seven compounds did not show >50% inhibition at 1 mM. In addition, no active component was identified in the extract of *S. chinensis*. Therefore, compound **1** was selected as a hit molecule to further investigate hTSLP-associated biological activities and for structural modification. Compound **1**, a flavonoid analog, was tested to treat allergy-related diseases^18,19^. It was also reported to reduce airway inflammation in ovalbumin (OVA)-treated mice^20^ and lower the skin severity scores in house dust mite (HDM)-challenged mice^21^.

**Table 1:** LC-QTOF MS/MS analysis of components in *S. baicalensis* extracts.

Peak No.	Compound identification	R_t_ (min)	observed *m/z*	MS/MS (m/z)	UV (λ_max_, nm)
**a**	chrysin-6-C-ara-8-C-glu	14.163	547.1447	337, 367, 457	273, 315
**b**	chrysin-6-C-glu-8-C-ara	14.600	547.1452	337, 427, 367	273, 315
**c**	2′,3,5,6′,7-pentahydroxyflavanone (**1g**)	15.350	303.0499	125, 177	285
**d**	baicalin (**1a**)	17.037	445.0772	269	275, 315
**e**	dihydrobaicalin	17.662	447.0925	271	285
**f**	baicalein-6-glucuronide	17.912	445.0767	269	280
**g**	oroxylin A-7-glucuronide	18.099	459.0925	283, 268	272, 314
**h**	viscidulin III (**1e**)	18.162	345.0604	330, 315	265
**i**	wogonoside	18.661	459.0925	283, 268	275
**j**	2′,5,6′,7-tetrahydroflavanone (**1f**)	19.036	287.0551	125, 161	—
**k**	baicalein (**1**)	21.473	269.0453	195, 136, 167	274, 325
**l**	wogonin (**1b**)/skullcapflavone II (**1d**)	24.283	283.0612/373.0933	268, 163/358, 343	275
**m**	chrysin	24.534	253.0506	63, 143	—
**n**	oroxylin A (**1c**)	24.971	283.0614	268, 109	—

**Table 2:** hTSLP-inhibitory activities of compounds (**1** and **1a**–**1g**) by ELISA.

Compound	% inhibition	
	0.3 mM	1 mM
**1**	52.5 ± 2.3	77.5 ± 1.6
**1a**	12.1 ± 1.8	19.9 ± 0.2
**1b**	<5	<5
**1c**	<5	11.6 ± 6.3
**1d**	<5	<5
**1e**	12.8 ± 0.6	26.3 ± 0.0
**1f**	14.0 ± 3.8	8.4 ± 5.8
**1g**	<5	<5

### Binding of compound 1 to hTLSP

To investigate whether compound **1** binds to hTSLP, we performed NMR binding studies based on NMR signals broadening using a T_2_ relaxation experiment^22,23^. In addition, we established an NMR ligand binding assay for hTSLP using a Carr-Purcell-Meiboom-Gill (CPMG) relaxation-edited 1D spectroscopy. We measured the NMR signals of **1** at a concentration of 100 μM and monitored signal changes following the addition of hTSLP at concentrations ranging from 2.5 to 10 μM. We compared signal changes observed in the presence of hTSLP to those of other proteins including hTSLPR and carbonic anhydrase. When a small molecule binds to a protein within μM to mM range (medium to low-affinity), the intensities of the bound molecules are greatly reduced because of T_2_ relaxation, whereas unbound compounds do not show a significant reduction. In addition, reduction in signal intensities is also seen with chemical exchange processes in millisecond order. Thus, comparison of the NMR signal intensities of compounds in the absence or presence of a protein provides valuable information on the binding properties of a compound to the target protein.

**Figure 2:**
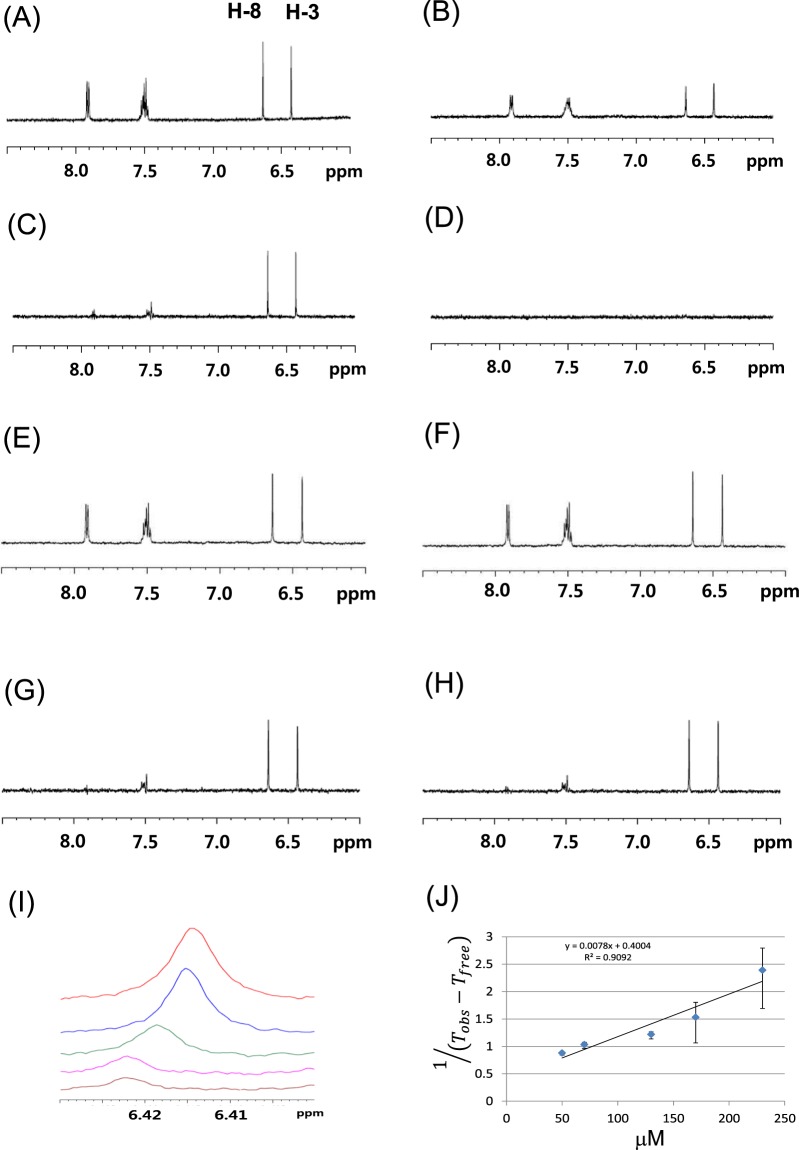
(**A**–**D**) Series of 1D NMR spectra of **1** in the aromatic region in the absence (**A** and **C**) or presence (**B** and **D**) of hTSLP. Normal 1D spectra of **1** (**A** and **B**), and 1D relaxation-edited NMR spectra with 400 ms-long CPMG pulse sequences (**C** and **D**). (**E**,**F**) Series of ^1^H 1D NMR spectra of **1** in aromatic region in the presence of hTSLPR (**E**) and carbonic anhydrase (**F**). (**G**,**H**) 1D relaxation-edited NMR spectra of **1** in aromatic region in the presence of hTSLPR (**G**) and carbonic anhydrase (**H**). (**I**) ^1^H NMR spectra of H3 signal of **1** at various concentrations. (**J**) Plot of the equation, $1/({T}_{obs}-{T}_{free})$, *versus* concentration of **1**. The line was determined using weighted linear least-squares fit.

In the phosphate-buffered saline (PBS) buffer condition, 1D NMR signals of **1** were significantly broadened in the presence of hTSLP (Fig. 2A *vs*. B), whereas no signal broadening was observed following incubation with other proteins such as TSLPR and carbonic anhydrase (Fig. 2E *vs*. F). Results showed that the signals of **1** completely disappeared in the presence of hTSLP upon the enhancement of T_2_ relaxation using the CPMG pulse (Fig. 2C *vs*. D). However, these signals were minimally affected in the presence of hTSLPR or carbonic anhydrase (Fig. 2G *vs*. H). These results indicate that compound **1** potentially binds to hTSLP and inhibit the hTSLP/hTSLPR signaling pathway. The signal broadening effects of **1** by hTSLP were dose-dependent. The *K*_*d*_ value of compound **1** was 50.3 μM (Fig. 2I,J), calculated according to the method described by Miller *et al*.^22^. The pull-down assay was performed using a His-tagged hTSLPR and a FLAG-tagged hTSLP and determined by Western blotting (See Supporting Information). The amount of the FLAG-tagged hTSLP bound to the His-tagged hTSLPR was reduced in a dose-dependent manner with the addition of compound **1** (0, 10, 50, and 100 μM). In addition, we determined the *K*_*d*_ value of compound **1** (27 μM) by microscale thermophoresis (See Supporting Information).

**Figure 3:**
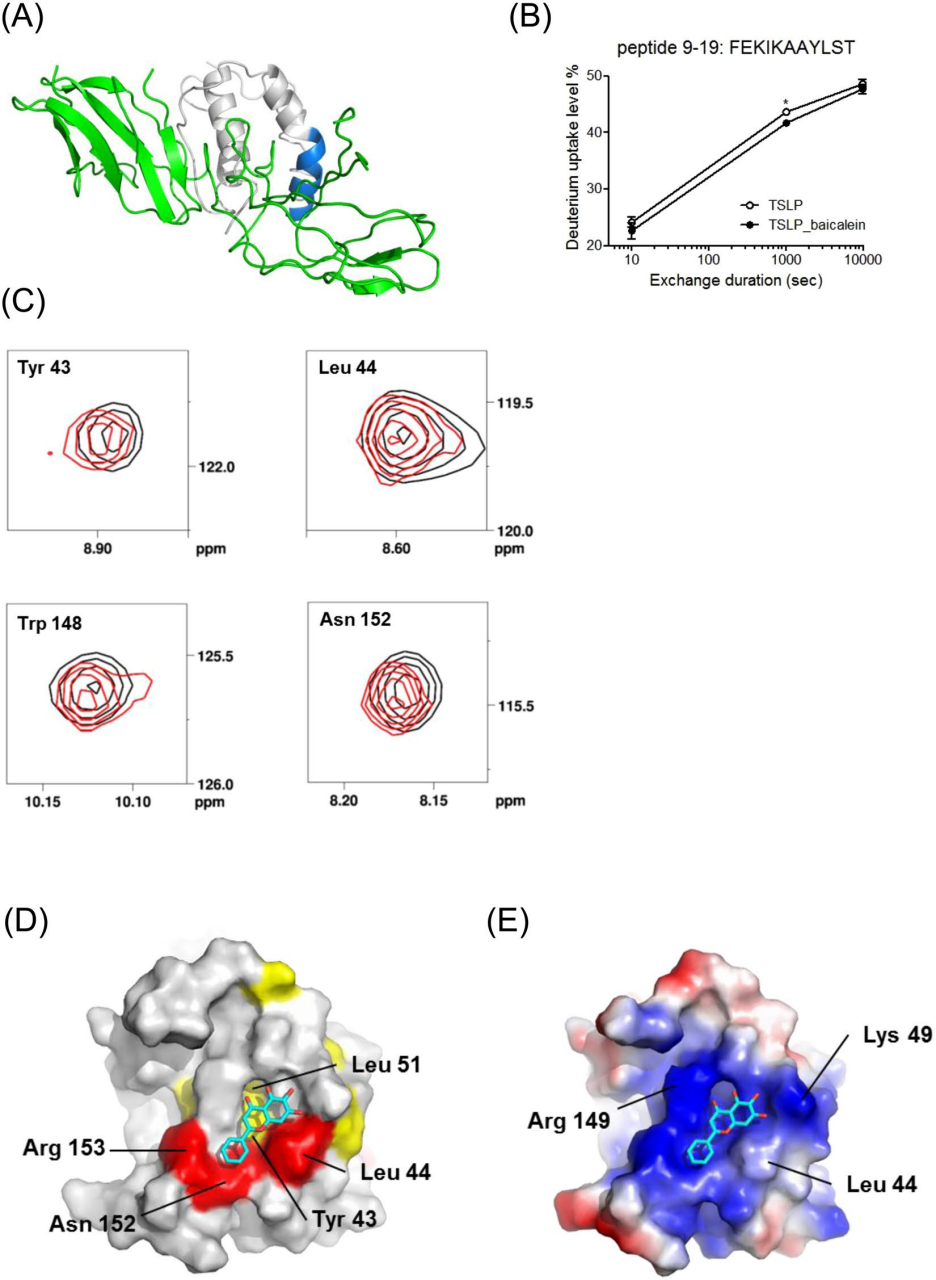
(**A**) Hydrogen-deuterium exchange (HDX) of **1** in hTSLP measured using MS. Deuterium uptake profiles are color-coded onto the modeled structure of hTSLP. Regions showing lower and constant deuterium uptake after binding of **1** are colored blue and grey, respectively, whereas hTSLPR is indicated in green. (**B**) Deuterium uptake level plot of the blue-colored region. (**C**) CSP in the ^1^H-^15^N HSQC spectrum of ^15^N-labeled hTSLP in the presence (red) and absence (black) of **1** in 1:4 molar ratio. The expanded spectra for the amide signals of the residues Tyr 43, Leu 44, Asn 152, and Arg 153 were presented. (**D**) Mapping of the CSP results on the surface of hTSLP. Red and yellow color denotes strongly (CSP > 0.014) and weakly (0.011 < CSP < 0.014) perturbated residues, respectively. Compound **1** is shown as a stick model in cyan color. (**E**) Modeled structure of compound **1** bound in the pocket of hTSLP. The key residues of hTSLP interacting with compound **1** were denoted. Surface electrostatic potentials are shown in blue and red color for positive and negative charges, respectively.

The binding site of **1** in hTSLP was confirmed using hydrogen-deuterium exchange (HDX)-MS. HDX-MS monitors the exchange between deuterium in the solvent and backbone amide hydrogen, which generally provides information on the binding of a compound to a protein^24,25^. Following the addition of **1**, the *N*-terminal half of the H1 helix in hTSLP showed decreased deuterium uptake as illustrated in Fig. 3A,B. As the *N*-terminal residue (FEKIKAAYLST) is positioned close to the hTSLPR, compound **1** might bind to the interface of the hTSLP-hTSLPR interaction. In order to identify the binding site of **1** on hTSLP, we performed chemical shift perturbation (CSP) experiments using ^15^N-labeled ${{\rm{TSLP}}}_{29-159{\rm{\Delta }}127-131}$ with **1**. Our results revealed chemical shift changes of the perturbated signals in the NMR spectrum of hTSLP following the binding of **1**. The backbone amide group of Leu 44, Leu 93, Ile 108, Tyr 113, Asn 152 and Arg 153 showed strong CSP (Δδ > 0.014) as shown in Fig. 3C. Amino acid residues including Phe 36, Tyr 43, Ile 47, Asp 50, Thr 58, Cys 75, Glu 78, Ser 81, Leu 93, Leu 106, Ile 108, Leu 144, and Gln 145 showed weak CSP (0.011 < Δδ < 0.014) after the binding of **1** (Fig. 3D).

Furthermore, we analyzed the binding mode of **1** on the surface of hTSLP using molecular docking simulations. Computer-aided binding analysis of **1** and hTSLP revealed that **1** was bound to the positively charged pocket (Lys 49 and Arg 149) through its hydroxyl groups, and the B ring of **1** interacted with the hydrophobic residues including Tyr 43 and Leu 44 (Fig. 3E). Docking studies of **1** with hTSLP suggested that **1** bound to the hTSLPR-binding interface of hTSLP.

### Compound 1 inhibited hTSLP-hTSLPR interaction and hTSLP signaling

**Figure 4:**
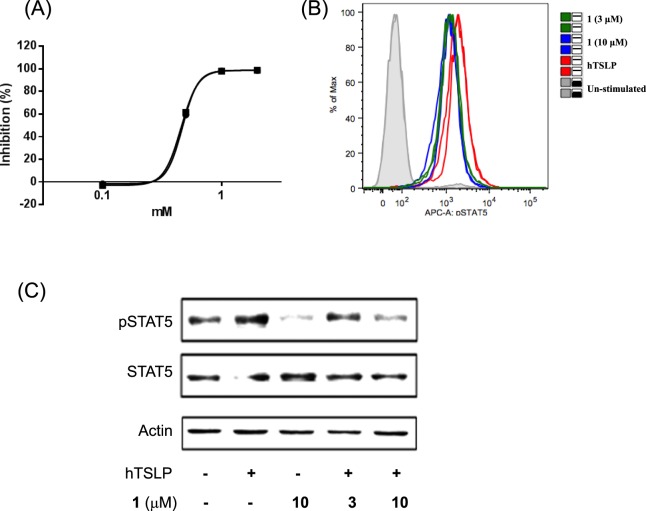
(**A**) Percentage inhibition plot of **1** measured by ELISA. (**B**) Flow cytometric analysis of STAT5 phosphorylation levels in hTSLP-stimulated HMC-1 cells. HMC-1 cells were pretreated with **1** (3 or 10 μM) and then stimulated with hTSLP (100 ng/mL) for 30 min. (**C**) Western blot analysis of STAT5 phosphorylation levels. HMC-1 cells were pretreated with **1** (3 or 10 μM) and then stimulated with hTSLP (20 ng/mL) for 2 h.

To understand the function of **1** in hTSLP signaling, we performed a series of bioassays. First, an ELISA was performed to determine the effect of **1** on the interaction between hTSLP and hTSLPR. We constructed vectors expressing hTSLP with the *N*-terminal FLAG tag (FLAG-hTSLP) and hTSLPR with *C*-terminal octa-histidine tag (hTSLPR-his). Compound **1** inhibited the interaction between hTSLP and hTSLPR in a dose-dependent manner as shown in Fig. 4A. In addition, we established a cell-based assay to monitor STAT5 phosphorylation in human mast cell line-1 (HMC-1) cells after stimulation with hTSLP. Treatment with hTSLP increased STAT5 phosphorylation, which was inhibited by **1** as shown in Fig. 4B. Western blot (Fig. 4C) analyses also showed the dose-dependent inhibition of STAT5 signaling by **1**. These results demonstrated that **1** inhibited the interaction between hTSLP and hTSLPR, which further inhibited STAT5 phosphorylation in hTSLP-stimulated cells.

### Compound 1 inhibited eosinophil infiltration in HDM-challenged mice

**Figure 5:**
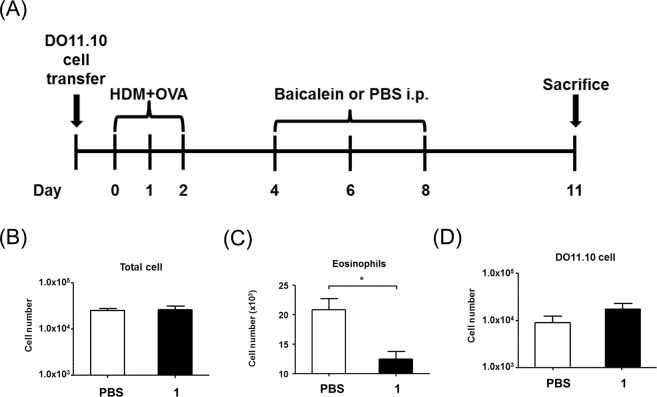
(**A**) Experimental design. On day 11 post allergen challenge, BAL was performed to determine eosinophilic inflammation. (**B**) Total cells from airways were counted to determine differential immune responses between the control and HDM-challenged groups. (**C**) Number of eosinophils assessed using cytospin followed by Diff-Quik staining. (**D**) Number of allergen-specific T cells was determined by flow cytometry. Data are means of three independents experiments (3/group). Values are mean ± standard error of the mean (SEM) (*P < 0.05).

To determine the effect of **1** on TSLP *in vivo*, we used a mouse model of airway inflammation, which is predominantly mediated by TSLP and Th2 cells^26^. Naïve mice receiving OVA-specific DO11.10 CD4 T cells were challenged with a mixture of house dust mite (HDM) and OVA for 3 days (Fig. 5A). These mice were then treated with either PBS or **1** (200 μg/mL) on day 4, 6, and 8 as indicated. On day 11 after the first challenge, the mice were euthanized and analyzed for eosinophilic inflammation. Although the total number of leukocytes in the bronchoalveolar lavage fluid (BALF) was comparable to that in the PBS control (Fig. 5B), the number of eosinophils was substantially reduced in mice treated with **1** compared with the PBS-treated control (Fig. 5C). Since this model relies on the function of Th2 cells, we measured the number of allergen-specific T cells, which interestingly was not changed by compound **1** treatment (Fig. 5D).

### Effect of compound 1 on pulmonary eosinophilia in OVA-sensitized and challenged mice

**Figure 6:**
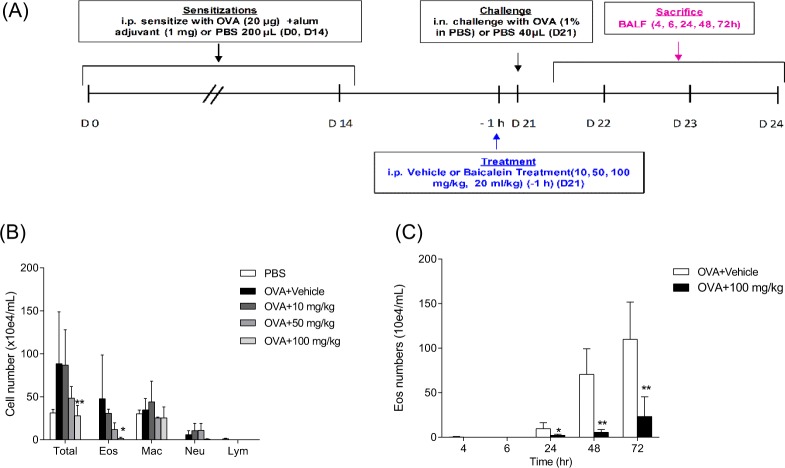
(**A**) Experimental design. Female BALB/c mice were sensitized and challenged with OVA. IP sensitization of OVA was performed on day 0 and 14. Vehicle and **1** (10, 50, or 100 mg/kg) were administrated 1 h prior to intranasal challenge on day 21. For control group (vehicle only), number of eosinophils in the BALF was determined on day 23. For groups treated with **1**, BALF was obtained at the indicated time points (4, 6, 24, 48, and 72 h). (**B**) Dose-dependent inhibition of OVA-induced eosinophilia by **1**. BALF cells were assessed 48 h after administration of vehicle or different doses of **1** (10, 50, or 100 mg/kg). Eos = eosinophils; Mac = macrophages; Neu = neutrophils; Lym = lymphocytes. (**C**) Temporal profile of eosinophil numbers in the BALF of mice pretreated with 100 mg/kg of **1** at 1 h before OVA challenge (n = 3–4). Values are means ± SEM (*P < 0.05 and **P < 0.01).

To determine the effect of **1** on pulmonary eosinophilia in mice, a murine model of OVA-induced pulmonary eosinophilia was used (Fig. 6A)^27–29^. Eosinophil-rich inflammation is the hallmark feature of both asthma in humans and allergic airway inflammation in mice^30^. A single intraperitoneal (IP) administration of **1** inhibited airway inflammation in a dose-dependent manner (Fig. 6B). At a dose of 100 mg/kg, the number of eosinophils recruited to the airways was significantly reduced. We further assessed the temporal relationship between the dose of **1** and airway eosinophilia to confirm the anti-inflammatory activity of compound **1**. As expected, administration of 100 mg/kg decreased the eosinophil numbers as early as 24 h post injection, and persisted for up to 72 h (Fig. 6C).

### Strategy for structural modification of compound 1

**Figure 7:**
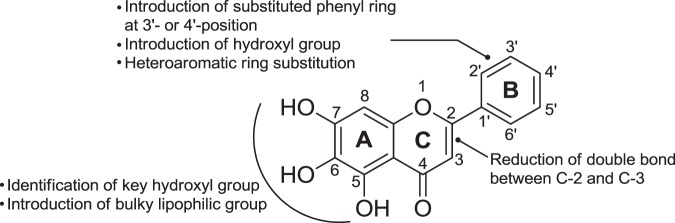
Strategy for structural modification of compound **1**.

The biological evaluation of compound **1** suggested that targeting hTSLP signaling using small molecules is possible and could be a promising treatment option for allergic diseases. Based on the chemical structure of **1**, the SAR studies focused on three purposes: to identify the essential OH groups, improve the physicochemical properties, and synthesize new analogs of **1**. As shown in Fig. 7, compound **1** was subdivided into three ring regions: A, B, and C. First, we aimed to investigate the necessity of the three hydroxyl groups in ring A of **1** for hTSLP inhibition. Second, we intended to introduce a biphenyl moiety instead of the phenyl group in ring B because hydrophobic amino acid residues were observed near the binding site of **1** to hTSLP in the *in silico* docking studies. Third, we attempted to elucidate the planarity of the ring C on hTSLP-binding. Reduction of the double bond between C-2 and C-3 carbons was expected to convert the planar flavone structure into a non-flat flavanone structure.

### Analog synthesis of compound 1

**Figure 8:**
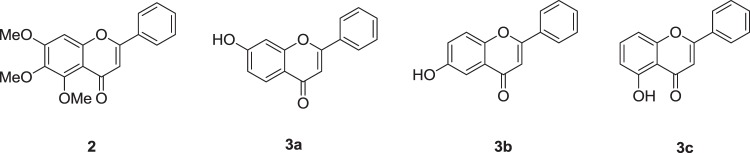
Chemical structure of **2** and **3a–3c**.

In order to determine the importance of the hydroxyl groups in the A ring for hTSLP inhibition, we converted three OH groups of **1** into corresponding methoxy groups (**2**) by reacting **1** with methyl iodide in acetone^31^. The hTSLP inhibitory activities of **2** and commercial mono-hydroxylated flavones (**3a–3c**, Fig. 8) were measured using ELISA. None of the compounds showed >50% inhibition at 1 mM, suggesting that at least two OH groups are needed to block the interaction between hTSLP and hTLSPR (Table 3).

**Table 3:** hTSLP-inhibitory activities of compounds (**2** and **3a–3c**) by ELISA.

Compound	% inhibition	
	0.3 mM	1 mM
**1**	43.1 ± 1.0	57.1 ± 1.7
**2**	<5	12.0 ± 2.8
**3a**	16.4 ± 2.7	23.3 ± 6.8
**3b**	22.2 ± 2.5	38.7 ± 5.3
**3c**	24.1 ± 5.4	16.8 ± 2.0

**Figure 9:**
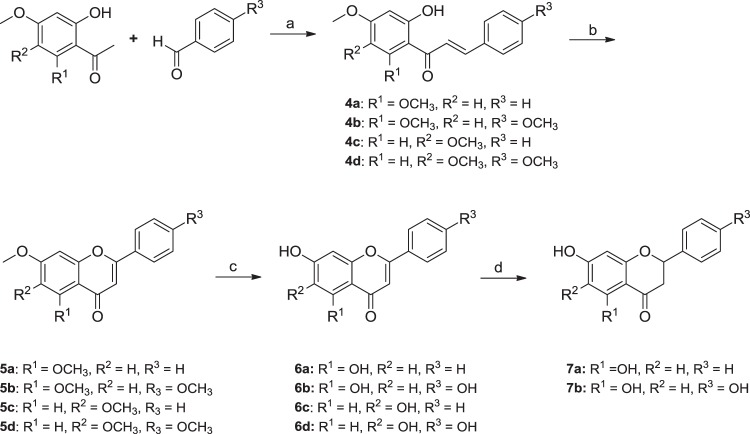
Synthesis of flavone analogs (**6a**–**6d**) with dihydroxyl groups at A ring and their corresponding flavanone analogs (**7a**–**7b**). Reagents and conditions: (a) NaOCH_3_ (1.2 eq), THF, 0 °C, rt, 8 h; (b) I_2_ (1.1 eq), DMSO, 130 °C, 3 h; (c) BBr_3_ (10–15 eq), CH_2_Cl_2_, reflux, 12 h, (d) H_2_, Pd/C, 1,4-dioxane, methanol, rt, 24 h.

**Figure 10:**
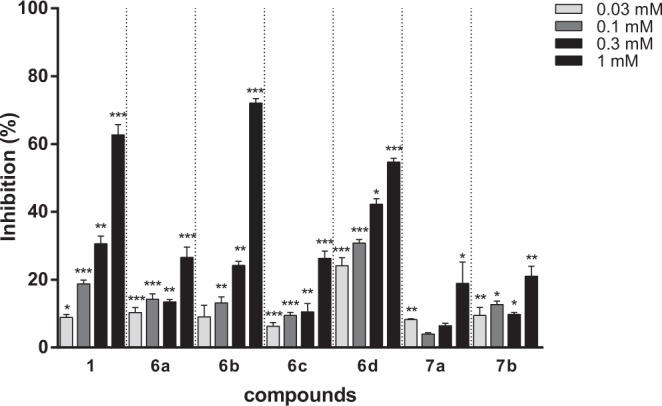
hTSLP-inhibitory activities of flavone analogs (**6a–6d**) and flavanone analogs (**7a–7b**).

Next, di-hydroxylated flavones (**6a–6d**) and flavanones (**7a–7b**) were synthesized in four or five steps from commercially available 2′-hydroxy-dimethoxyacetophenones and appropriate benzaldehydes (Fig. 9). Briefly, condensation of acetophenone with benzaldehyde (benzaldehyde for **4a** and **4c**, and *p*-anisaldehyde for **4b** and **4d**) in THF under basic condition produced the corresponding chalcones **4a–4d** in 64–98% yield. Treatment of **4a–4d** with iodine powder in DMSO provided the flavone compounds **5a–5d** in 38–78% yield^32^. Demethylation of **5a–5d** was achieved by treating BBr_3_ in CH_2_Cl_2_ under reflux to afford the flavone analogs **6a–6d** in 47–82% yield^33,34^. Reaction temperature and maintenance of anhydrous reaction conditions were critical for the demethylation step. Reflux condition provided the desired fully-demethylated compounds **6a–6d** while reaction at room temperature resulted in mono-demethylated compounds as major products. Hydrogenation of compounds **6a–6d** was carried out in the presence of Pd/C and H_2_^35^. The 5,7-dihydroxylated flavones (**6a**–**6b**) were converted into the corresponding flavanones (**7a–7b**) in 56–74% yield. However, the 6,7-dihydroxylated flavones (**6c–6d**) remained intact under the same condition. As the intramolecular hydrogen bonding between the OH group of the A ring and the carbonyl group of the B ring was significantly increased in the 5,7-dihydroxylated flavones, compounds **6a–6b** were more reactive than the 6,7-dihydroxylated flavones **6c–6d** towards catalytic hydrogenation. The conversion of the planar flavone to the non-flat flavanone structure was expected to affect hTSLP binding and physicochemical properties. Flavanone analogs showed weaker hTSLP-inhibitory activities than the corresponding flavones did (**7a** *vs*. **6a** and **7b** *vs*. **6b**) as shown in Fig. 10. However, the kinetic solubility of the flavanones (111 μM and 505 μM for **7a** and **7b**, respectively) in PBS was increased compared to that of the corresponding flavones (30 μM and 486 μM for **6a** and **6b**, respectively). Therefore, the flavanone scaffold could be utilized to improve the physicochemical properties of flavones.

**Figure 11:**
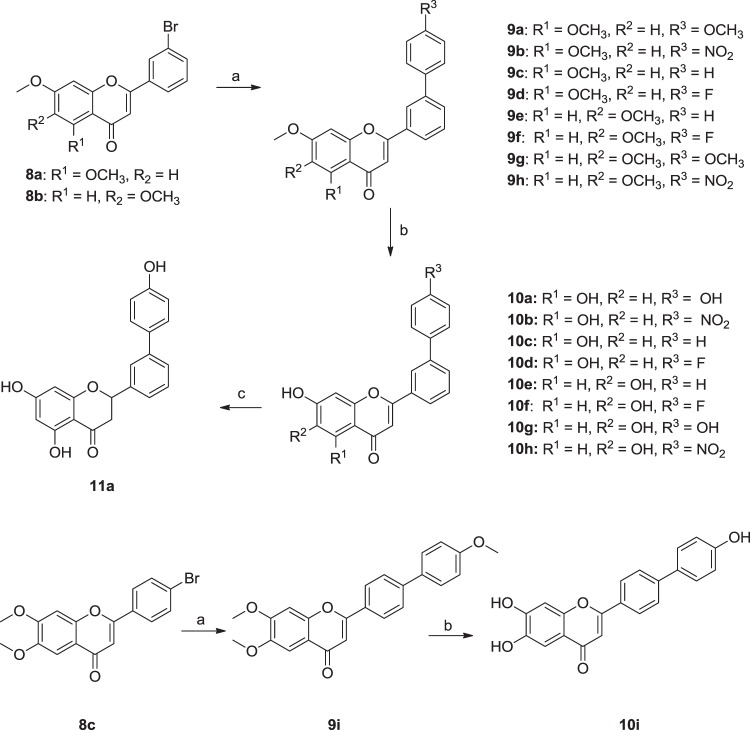
Synthesis of biphenyl-derived flavone analogs (**10a**–**i**) and flavanone analog (**11a**). Reagents and conditions: (a) appropriate benzeneboronic acid, Pd(PPh_3_)_4_, toluene, H_2_O, Cs_2_CO_3_, 90 °C, 5 h; (b) BBr_3_ (10–15 eq), CH_2_Cl_2_, reflux, 12 h; (c) H_2_, Pd/C, 1,4-dioxane, methanol, rt, 24 h.

To increase the hTSLP-binding affinity, a lipophilic ring-extended biphenyl group was introduced into the C ring region. Ring-extended compounds **10a–10i** were obtained in two steps from bromoflavones **8a–8c** (Fig. 11), which contained a bromo group on the 3′- or 4′-position of B ring. They were prepared from 2′-hydroxy-dimethoxyacetophenones and bromobenzaldehydes by applying the synthetic procedure described in Fig. 9. Reaction of 3′-bromoflavones (**8a–8b**) or 4′-bromoflavone (**8c**) with appropriate benzeneboronic acid (benzeneboronic acid for **9c** and **9e**, 4-fluorobenzeneboronic acid for **9d** and **9f**, 4-nitrobenezeneboronic acid for **9b** and **9h**, and 4-methoxybenzeneboronic acid for **9a**, **9g**, and **9i**) in the presence of tetrakis(triphenylphosphine)palladium at 90 °C for 5 h afforded the biphenyl compounds **9a–9i** in 23**–**78% yield. Demethylation of **9a–9i** by treatment of BBr_3_ under reflux for 12 h afforded the final compounds **10a–10i** in 13**–**46% yield^33,34^. Reduction of the C ring was attempted under hydrogenation conditions (Pd/C, H_2_). Hydrogenation of 6,7-dihydroxylated biphenyl analogs (**10e–10i**) did not occur under this condition as observed with compounds **6c–6d** and the starting materials were recovered. Among the 5,7-dihydroxylated biphenyl flavones (**10a–10d**), only compound **10a** was reduced to provide compound **11a** in 68% yield. The higher reactivity of compound **10a** than the other 5,7-dihydroxylated analogs might be explained by the stability of its carbocation resonance structure (See Supporting Information). As the OH group of the biphenyl ring of **10a** increased electron-releasing effect toward the C ring, the carbocation structure of **10a** was more stabilized and the single-bond character between C-2 and C-3 was enhanced than that of the others. When acetic acid was added in the hydrogenation step to increase the reactivity of compounds **10b–10d**, the flavanone ring was broken and no desired product was obtained.

**Figure 12:**
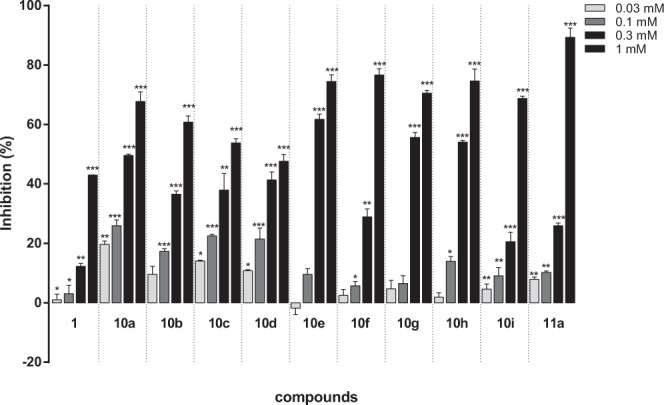
hTSLP-inhibitory activities of flavone analogs (**10a**–**10i**) substituted with biphenyl moiety and flavanone compound **11a**.

Biphenyl-based flavones (**10a–10i**) showed increased TSLP-inhibitory activities compared to **1** (Fig. 12), which was used as the positive control in the ELISA assay. In particular, four compounds (**10a**, **10e**, **10g**, and **10h**) exhibited >50% inhibition at 0.3 mM. The 6,7-dihydroxy analogs (**10e–10h**) displayed slightly stronger hTSLP-inhibition than that of the 5,7-dihydroxy analogs (**10a–10d**). In addition, the introduction of a phenyl group at the 3′-position of the B ring (**10f**) was more favorable for hTSLP binding than at the 4′-position of the B ring (**10i**). The combination of dihydroxyl groups on the 6- and 7-positions with the phenyl ring at the 3′-position of the B ring enhanced hTSLP-binding affinity compared to that of **1**. Compounds **10e** and **10g** were the most potent biphenyl analogs with IC_50_ values of 177 and 210 μM, respectively. Western blot analyses also showed that **10e** and **10g** inhibited STAT5 phosphorylation stronger than compound **1** (See Supporting Information). However, the solubility of the biphenyl compounds in PBS (pH 7.4) was much lower than that of compound **1**.

**Figure 13:**
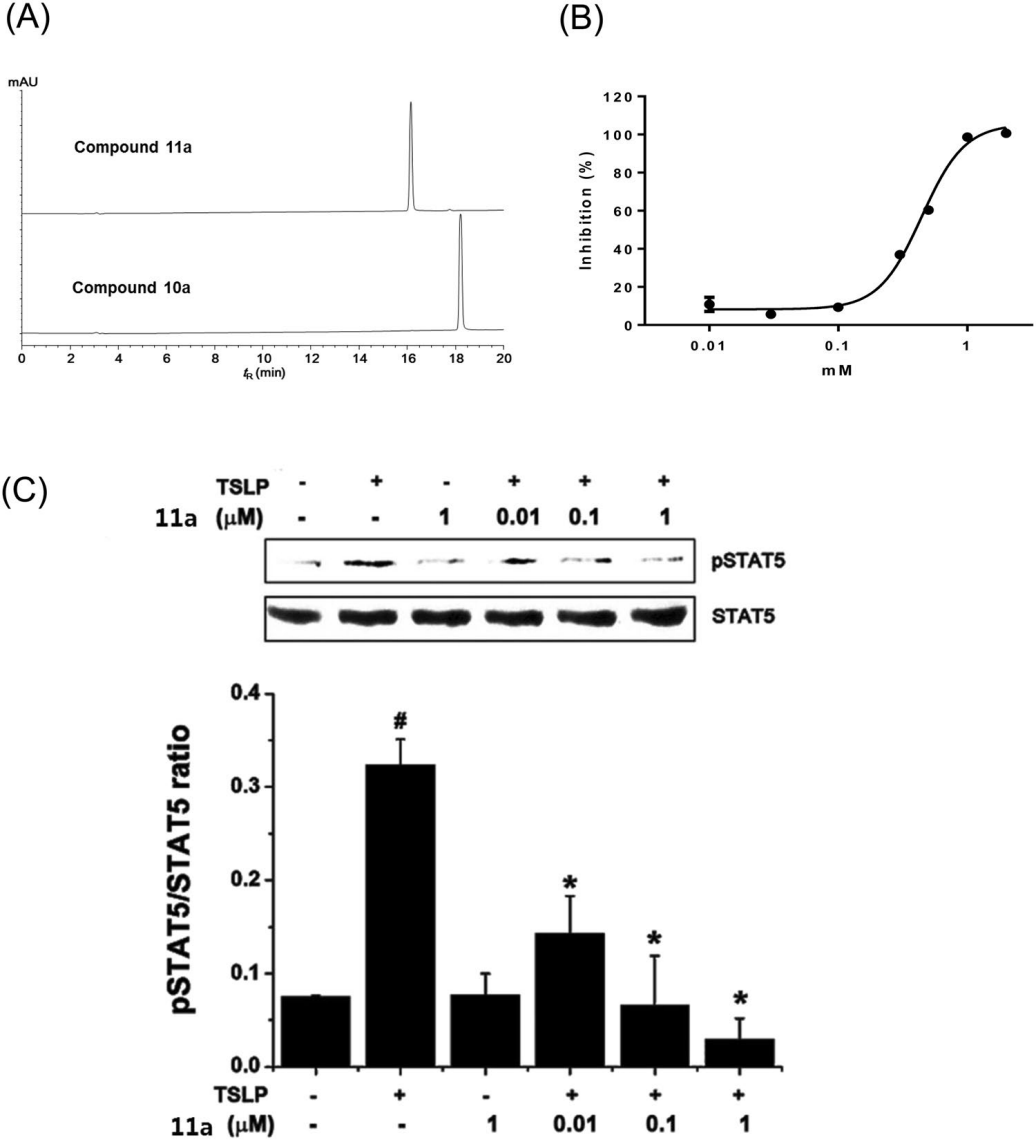
(**A**) HPLC chromatograms of **11a** (R_t_ = 16.152 min) and **10a** (R_t_ = 18.214 min). (**B**) Plot of percentage inhibition at different concentrations of **11a** measured by ELISA. (**C**) Western blot analysis of pSTAT5 levels. HMC-1 cells were pretreated with **11a** (0.l, 1, and 10 μM) and then stimulated with TSLP (20 ng/mL) for 2 h. The levels of pSTAT5 were analyzed by western blotting (upper panel). The relative densities were quantified by densitometry means pSTAT5/STAT5×100 (lower panel). Data represent the mean ± S.E.M. of three independent experiments. ^#^P < 0.05; significantly different from unstimulated cells’ value. *P < 0.05; significantly different from TSLP’ value.

Biphenyl-derived flavanone compounds were designed and expected to be more soluble in PBS than the corresponding flavones as observed with compounds **7a**–**7b**. Catalytic hydrogenation of the C ring under traditional reduction condition (Pd/C, H_2_) did not alter the 6,7-dihydroxylated biphenyl flavones (**10e–10i**) as expected. Only **10a** was reduced to provide the flavanone analog **11a** among 5,7-dihydroxylated analogs (**10a–10d**). The increased hydrophilicity of **11a** compared with the flavone **10a** was observed in reversed-phase HPLC experiments (16.15 min and 18.21 min for **11a** and **10a**, respectively Fig. 13A). In addition, the kinetic aqueous solubility of **11a** (66 μM) was comparable to that of **1** (62 μM). The IC_50_ value of the flavanone **11a** was 370 μM in the ELISA (Fig. 13B), showing that it was slightly more potent than **1** (IC_50_ = 460 μM). Furthermore, compound **11a** strongly inhibited STAT5 phosphorylation even at 0.01 μM at the western blot experiment (Fig. 13C). Therefore, the biphenyl-derived flavanone **11a** which possesses moderate hTSLP-inhibition and good water solubility could be a prototype molecule for further structural modification in the development of novel hTSLP inhibitors.

## Conclusion

Recent studies of TSLP/TSLPR signaling pathways have demonstrated the key role of hTSLP in allergic immune responses. In this work, we identified compound **1**, a flavonoid from *S. baicalensis*, as the first small molecule inhibitor of the hTSLP signaling pathway. *In vitro* studies including ELISA, flow cytometry, and Western-blot analysis showed that **1** blocked the interaction between hTSLP and hTSLPR in a dose-dependent manner. The HDX-MS experiment also confirmed that **1** binds to the hTSLPR-binding interface of hTSLP. *In vivo* studies in HDM-challenged mice showed that **1** substantially reduced eosinophilic inflammation. Furthermore, a single treatment with **1** effectively reduced eosinophil-rich pulmonary inflammation in the OVA-induced animal model. According to the SAR studies of compound **1**, at least two OH groups in the A ring are required for hTSLP inhibition. Introduction of a hydrophobic biphenyl moiety in the B ring increased hTSLP-binding affinity. In addition, conversion of flavone into a flavanone structure in the C ring improved water solubility. Compound **11a** was identified to be the most advanced hTSLP inhibitor in this series with moderate hTSLP-inhibition as well as good water solubility. Blocking hTSLP by using small molecule inhibitors may provide a new strategy to treat allergic diseases.

## Experimental Procedures

### General

All the chemicals and solvents used in the reaction were purchased from Sigma-Aldrich, TCI, or Alfa Aesar, and were used without further purification. Reactions were monitored by TLC on 0.25 mm Merck precoated silica gel plates (60-F_254_). Reaction progress was monitored by TLC analysis using a UV lamp and/or KMnO_4_ staining for detection purposes. Column chromatography was performed on silica gel (230–400 mesh, Merck, Darmstadt, Germany). NMR spectra were recorded at room temperature on either Bruker BioSpin Avance 300 MHz NMR or Bruker Ultrashield 600 MHz Plus spectrometer. Chemical shifts are reported in parts per million (ppm, *δ*) with TMS as an internal standard. Coupling constants are given in Hertz (Hz). Splitting patterns are indicated as s, singlet; d, doublet; t, triplet; q, quartet; m, multiplet; br, broad for ^1^H NMR data. Mass spectra were obtained on an Agilent 6530 Accurate mass Q-TOF LC/MS spectrometer or an electrospray ionization PE Biosystems Sciex Api 150 EX mass spectrometer single quadruple equipped with a turbo ion spray interface. The purity of all final compounds was measured by analytical reversed-phase (RP) HPLC on an Agilent 1260 Infinity (Agilent) with a C18 column (Gemini-NX, 150 mm × 4.6 mm, 3 μm, 110 Å). RP-HPLC was performed using a linear gradient elution 50 to 100% of solvent B over 20 min (A = 0.1% TFA in water and B = 0.1% TFA in methanol). All compounds were eluted with a flow rate 0.5 mL/min and monitored at UV detector: 254 nm. Purity of final compounds was >95%.

### HPLC Q-TOF/MS analysis

HPLC analysis was performed on a Shiseido CapCell PAK C18 column (5 µm, 4.6 mm I.D. × 150 mm) with a C18 guard column (4.00 × 3.00 mm; Phenomenex, Torrance, CA, USA). The flow rate was 0.6 mL/min, and injection volume was 10 µL. The mobile phase consisted of water containing 0.1% formic acid (A) and acetonitrile containing 0.1% formic acid (B). Gradient elution was as follows: 0–5 min, 10% B, 5–30 min, and 10–90% B. After UV detection, the eluted fractions were used for mass chromatography analysis. The elution fractions were ionized by ESI in positive mode, and MS and MS/MS spectrometric data were acquired in parallel alternating scan mode. The mass parameters were as follows: nebulizer pressure, 40 psi; capillary voltage, 4000 V; fragmentor voltage, 175 V; skimmer voltage, 65 V; drying gas temperature, 325 °C; flow rate of drying gas, 12.0 L/min; collision energy, 10, 20, and 30 V; mass scan range, *m/z* 50–1700. All acquisition parameters were adjusted using MassHunter Workstation software LC/MS Data Acquisition for 6530 series Q-TOF (version B.05.00).

### Synthesis

#### General procedure for chalcone synthesis

To a solution of 2′-hydroxy-4′,5′-dimethoxyacetophenone or 2′-hydroxy-4′,6′-dimethoxyacetophenone in THF was added sodium methoxide (1.1 eq) in methanol solution at 0 °C and stirred for 20 min. Appropriate benzaldehyde (1.2 eq) was added to the reaction mixture at the same temperature. The mixture was stirred at room temperature for 8 h. The reaction mixture was partitioned and diluted with ethyl acetate (40 mL) and saturated ammonium chloride solution (40 mL). The organic layer was collected, dried over magnesium sulfate, and concentrated under reduced pressure. The residue was purified using flash column chromatography or recrystallization with hexane-ethyl acetate to give the corresponding chalcones (**4a–4d**).

*(E)-1-(2-Hydroxy-4,6-dimethoxyphenyl)-3-phenylprop-2-en-1-one* (**4a**)

2′-Hydroxy-4′,6′-dimethoxyacetophenone and benzaldehyde were used as starting materials. Purification by flash column chromatography (eluting with Toluene-E.A 50:1, v/v) provided **4a** as a yellow solid (yield = 91%). R_f_ = 0.33 (hexane‒E.A = 6:1, v/v). ^1^H NMR (300 MHz, CDCl_3_) *δ* 14.18 (s, 1H), 8.85 (d, *J* = 1.5 Hz, 1H), 8.60 (dd, *J* = 1.3 and 4.7 Hz, 1H), 7.97 (d, *J* = 15.7 Hz, 1H), 7.88 (d, *J* = 8.0 Hz, 1H), 7.74 (d, *J* = 15.7 Hz, 1H), 7.35 (dd, *J* = 4.9 and 7.8 Hz, 1H), 6.12 (d, *J* = 2.3 Hz, 1H), 5.98 (d, *J* = 2.3 Hz, 1H), 3.93 (s, 3H), 3.85 (s, 3H). HRMS (ESI) *m/z* calculated for C_17_H_16_O_4_ [M + H]^+^: 285.1121; found: 285.1134. ^1^H NMR and HRMS data were in complete agreement with that previously reported^36^.

*(E)-1-(2-Hydroxy-4,6-dimethoxyphenyl)-3-(4-methoxyphenyl)prop-2-en-1-one* (**4b**)

2′-Hydroxy-4′,6′-dimethoxyacetophenone and 4-methoxybenzaldehyde were used as starting materials. Purification by flash column chromatography (eluting with hexane-E.A, 5:1, v/v) provided **4b** in yellow solid (yield = 78%). R_f_ = 0.53 (hexane-E.A = 2:1, v/v). ^1^H NMR (300 MHz, CDCl_3_) *δ* 14.45 (s, 1H), 7.81 (s, 2H), 7.57 (d, *J* = 7.8 Hz, 2H), 7.94 (d, *J* = 8.4 Hz, 1H), 6.11 (s, 1H), 5.97 (s, 1H), 3.93 (s, 3H), 3.86 (s, 3H), 3.84 (s, 3H). HRMS (ESI) *m/z* calculated for C_18_H_18_O_5_ [M + H]^+^: 315.1227; found: 313.1141. ^1^H NMR and HRMS data were in complete agreement with that previously reported^37^.

*(E)-1-(2-Hydroxy-4,5-dimethoxyphenyl)-3-phenylprop-2-en-1-one* (**4c**)

2′-Hydroxy-4′,5′-dimethoxyacetophenone and benzaldehyde were used as starting materials. Purification by flash column chromatography (eluting with Toluene-E.A 30:1, v/v) provided **4c** in yellow solid (yield = 87%). R_f_ = 0.20 (hexane-E.A = 6:1, v/v). ^1^H NMR (300 MHz, CDCl_3_) *δ* 14.18 (s, 1H), 8.85 (d, *J* = 1.5 Hz, 1H), 8.60 (dd, *J* = 1.3 and 4.7 Hz, 1H), 7.97 (d, *J* = 15.7 Hz, 1H), 7.88 (d, *J* = 8.0 Hz, 1H), 7.74 (d, *J* = 15.7 Hz, 1H), 7.35 (dd, *J* = 4.9 and 7.8 Hz, 1H), 6.12 (d, *J* = 2.3 Hz, 1H), 5.98 (d, *J* = 2.3 Hz, 1H), 3.93 (s, 3H), 3.85 (s, 3H). HRMS (ESI) *m/z* calculated for C_17_H_16_O_4_ [M + H]^+^: 285.1121; found: 285.1134. ^1^H NMR and HRMS data were in complete agreement with that previously reported^38^.

*(E)-1-(2-Hydroxy-4,5-dimethoxyphenyl)-3-(4-methoxyphenyl)prop-2-en-1-one* (**4d**)

2′-Hydroxy-4′,5′-dimethoxyacetophenone and 4-methoxybenzaldehyde were used as starting materials. Purification by flash column chromatography (eluting with hexane-E.A, 5:1 to 3:1, v/v) provided **4d** in yellow solid (yield = 40%). R_f_ = 0.41 (hexane-E.A = 2:1, v/v). ^1^H NMR (300 MHz, CDCl_3_) *δ* 13.48 (s, 1H), 7.88 (d, *J* = 15.3 Hz, 1H), 7.64 (d, *J* = 8.7 Hz, 1H), 7.63 (dd, *J* = 4.5 and 9.6 Hz, 1H), 7.41 (d, *J* = 15.3 Hz, 1H), 6.97 (d, *J* = 8.7 Hz, 1H), 6.96 (dd, *J* = 4.5 and 9.6 Hz, 1H), 6.51 (s, 1H), 3.94 (s, 3H), 3.92 (s, 3H), 3.87 (s, 3H). HRMS (ESI) *m/z* calculated for C_18_H_18_O_5_ [M + H]^+^: 315.1227; found: 315.1242. ^1^H NMR and HRMS data were in complete agreement with that previously reported^39^.

#### General procedure for flavone synthesis

To a solution of chalcone in anhydrous DMSO was added iodine powder (1.1 eq) and stirred at 130 °C for 3 h. After being cooled to room temperature, the reaction mixture was diluted with ethyl acetate. The organic layer was washed with an aqueous solution of 0.1 M sodium thiosulfate, followed by the addition of brine. The organic layer was collected, dried over magnesium sulfate, and concentrated under reduced pressure. The residue was purified by flash column chromatography to give flavones (**5a–5d**).

*5,7-Dimethoxy-2-phenyl-4H-chromen-4-one* (**5a)**

*(E)*-1-(2-Hydroxy-4,6-dimethoxyphenyl)-3-phenylprop-2-en-1-one (**4a)** was used as the starting material. Purified by flash column chromatography (eluting with Toluene-E.A, 5:1 to 1:1, v/v) provided **5a** in white solid (yield = 43%). R_f_ = 0.32 (DCM-E.A = 4:1, v/v). ^1^H NMR (300 MHz, CDCl_3_) *δ* 7.87 (dd, *J* = 1.8 and 5.4 Hz, 2H), 7.50**–**7.51 (m, 3H), 6.69 (s, 1H), 6.57 (d, *J* = 2.4 Hz, 1H), 6.37 (d, *J* = 2.1 Hz, 1H), 3.96 (s, 3H), 3.92 (s, 3H). HRMS (ESI) *m/z* calculated for C_17_H_14_O_4_ [M + H]^+^: 283.0965; found: 283.0977. ^1^H NMR and HRMS data were in complete agreement with that previously reported^36^.

*5,7-Dimethoxy-2-(4-methoxyphenyl)-4H-chromen-4-one* (**5b**)

*(E)*-1-(2-Hydroxy-4,6-dimethoxyphenyl)-3-(4-methoxyphenyl)prop-2-en-1-one (**4b**) was used as the starting material. Purification by flash column chromatography (eluting with Toluene-E.A, 10:1 to 3:1, v/v) provided **5b** in white solid (yield = 72%). R_f_ = 0.55 (DCM-E.A = 2:1, v/v). ^1^H NMR (300 MHz, CDCl_3_) *δ* 7.83 (d, *J* = 8.1 Hz, 2H), 7.01 (d, *J* = 8.1 Hz, 2H), 6.61 (s, 1H), 6.57 (s, 1H), 6.38 (s, 1H), 3.96 (s, 3H), 3.92 (s, 3H), 3.89 (s, 3H). HRMS (ESI) *m/z* calculated for C_18_H_16_O_5_ [M + H]^+^: 313.1071; found: 313.1083. ^1^H NMR and HRMS data were in complete agreement with that previously reported^40^.

*6,7-Dimethoxy-2-phenyl-4H-chromen-4-one* (**5c**)

*(E)*-1-(2-Hydroxy-4,5-dimethoxyphenyl)-3-phenylprop-2-en-1-one (**4c)** was used as the starting material. Purification by flash column chromatography (eluting with DCM-E.A, 7:1 to 3:1, v/v) provided **5c** in white solid (yield = 38%). R_f_ = 0.38 (DCM-E.A = 4:1, v/v). ^1^H NMR (300 MHz, CDCl_3_) *δ* 7.92 (s, 1H), 7.57 (s, 1H), 7.54 (s, 3H), 7.02 (s, 1H), 6.81 (s, 1H), 4.03 (s, 3H), 4.00 (s, 3H). HRMS (ESI) *m/z* calculated for C_17_H_14_O_4_ [M + H]^+^: 283.0965; found: 283.0979. ^1^H NMR and HRMS data were in complete agreement with that previously reported^41^.

*6,7-Dimethoxy-2-(4-methoxyphenyl)-4H-chromen-4-one* (**5d**)

*(E)*-1-(2-Hydroxy-4,5-dimethoxyphenyl)-3-(4-methoxyphenyl)prop-2-en-1-one (**4d**) was used as the starting material. Purification by flash column chromatography (eluting with Toluene-E.A, 10:1 to 4:1, v/v) provided **5d** in white solid (yield = 78%). R_f_ = 0.33 (DCM-E.A = 5:1, v/v). ^1^H NMR (300 MHz, CDCl_3_) *δ* 7.87 (d, *J* = 9.0 Hz, 2H), 7.57 (d, *J* = 9.0 Hz, 2H), 7.00 (s, 1H), 6.72 (s, 1H), 4.02 (s, 3H), 4.00 (s, 3H), 3.90 (s, 3H). HRMS (ESI) *m/z* calculated for C_18_H_16_O_5_ [M + H]^+^: 313.1071; found: 313.1086. ^1^H NMR and HRMS data were in complete agreement with that previously reported^42^.

#### General procedure for demethylation (6a-6d)

To a solution of flavones (**5a–5b**) in anhydrous dichloromethane was added boron tribromide (5 eq) per methoxy functional group at 0 °C under argon atmosphere. The reaction mixture was stirred under reflux for 12 h. After being cooled to room temperature, the reaction mixture was quenched with iced water and concentrated under reduced pressure. The residue was partitioned between ethyl acetate and water. The water layer was adjusted to pH 7 and extracted with ethyl acetate. The organic layer was collected, dried over magnesium sulfate, and concentrated under reduced pressure. The residue was purified by flash column chromatography to give demethylated compounds (**6a–6d**).

*5,7-Dihydroxy-2-phenyl-4H-chromen-4-one* (**6a**)

5,7-Dimethoxy-2-phenyl-*4H*-chromen-4-one (**5a**) was used as the starting material. Purification by flash column chromatography (eluting with DCM-MeOH, 30:1 to 10:1, v/v) provided **6a** in white solid (yield = 82%). R_f_ = 0.72 (DCM-MeOH = 10:1, v/v). ^1^H NMR (300 MHz, CD_3_OD) *δ* 7.94 (d, *J* = 8.7 Hz, 2H), 7.52 (s, 3H), 6.69 (s, 1H), 6.43 (s, 1H), 6.17 (s, 1H). HRMS (ESI) *m/z* calculated for C_15_H_10_O_4_ [M − H]^−^: 253.0506; found: 253.0518. ^1^H NMR and HRMS data were in complete agreement with that previously reported^36^.

*5,7-Dihydroxy-2-(4-hydroxyphenyl)-4H-chromen-4-one* (**6b**)

5,7-Dimethoxy-2-(4-methoxyphenyl)-*4H*-chromen-4-one (**5b**) was used as the starting material. Purification by flash column chromatography (eluting with DCM-E.A, 10:1 to 3:1, v/v) provided **6b** in white solid (yield = 58%). R_f_ = 0.38 (DCM-E.A = 4:1, v/v). ^1^H NMR (300 MHz, CD_3_OD) *δ* 7.86 (d, *J* = 8.7 Hz, 2H), 6.94 (d, *J* = 8.7 Hz, 2H), 6.60 (s, 1H), 6.46 (s, 1H), 6.21 (s, 1H). HRMS (ESI) *m/z* calculated for C_15_H_10_O_5_ [M − H]^−^: 269.0455; found: 269.0452. ^1^H NMR and HRMS data were in complete agreement with that previously reported^43^.

*6,7-Dihydroxy-2-phenyl-4H-chromen-4-one* (**6c**)

6,7-Dimethoxy-2-phenyl-*4H*-chromen-4-one (**5c**) was used as the starting material. Purification by flash column chromatography (eluting with DCM-MeOH 10:1, v/v) provided **6c** in white solid (yield = 75%). R_f_ = 0.55 (DCM-MeOH = 10:1, v/v). ^1^H NMR (300 MHz, CD_3_OD) *δ* 7.85 (d, *J* = 4.8 Hz, 2H), 7.44 (s, 3H), 7.30 (s, 1H), 6.92 (s, 1H), 6.66 (s, 1H). HRMS (ESI) *m/z* calculated for C_15_H_10_O_4_ [M − H]^−^: 253.0506; found: 253.0518. ^1^H NMR and HRMS data were in complete agreement with that previously reported^44^.

*6,7-Dihydroxy-2-(4-hydroxyphenyl)-4H-chromen-4-one* (**6d**)

6,7-Dimethoxy-2-(4-methoxyphenyl)-*4H*-chromen-4-one(**5d**) was used as the starting material. Purification by flash column chromatography (eluting with DCM-MeOH = 30:1 to 10:1, v/v) provided **6d** in white solid (yield = 48%). R_f_ = 0.18 (DCM-MeOH = 10:1, v/v). ^1^H NMR (300 MHz, CD_3_OD) *δ* 7.87 (d, *J* = 7.8 Hz, 2H), 7.39 (s, 1H), 7.02 (s, 1H), 6.94 (d, *J* = 7.8 Hz, 2H), 6.67 (s, 1H). HRMS (ESI) *m/z* calculated for C_15_H_10_O_5_ [M − H]^−^: 269.0455; found: 269.0449. ^1^H NMR and HRMS data were in complete agreement with that previously reported^45^.

#### General procedure for the reduction of flavones to flavanones (7a-7b)

To a solution of flavones (**6a–6b**) in a mixture of 1,4-dioxane and methanol (4:1) was added palladium on carbon (10%). The reaction mixture was purged with hydrogen gas and stirred at room temperature for 24 h. The reaction mixture was filtered through Celite pad and concentrated under reduced pressure. The residue was purified by flash column chromatography provided flavanone compounds (**7a–7b**).

*5,7-Dihydroxy-2-phenylchroman-4-one* (**7a**)

5,7-Dihydroxy-2-phenyl-*4H*-chromen-4-one (**6a**) was used as the starting material. Purification by flash column chromatography (eluting with DCM-E.A 5:1, v/v) to give **7a** in white solid (yield = 76%). R_f_ = 0.53 (DCM-E.A = 3:1, v/v). ^1^H NMR (300 MHz, (CD_3_)_2_CO) *δ* 12.17 (s, 1H), 9.69 (brs, 1H), 7.57 (dd, *J* = 1.2 and 8.1 Hz, 2H), 7.37–7.49 (m, 3H), 5.98 (dd, *J* = 2.1 and 10.2 Hz, 2H), 5.58 (dd, *J* = 3.0 and 12.9 Hz, 1H), 3.18 (dd, *J* = 12.9 and 17.1 Hz, 1H), 2.81 (dd, *J* = 3.0 and 17.1 Hz, 1H). HRMS (ESI) *m/z* calculated for C_15_H_12_O_4_ [M − H]^−^: 255.0663; found: 255.0672. ^1^H NMR and HRMS data were in complete agreement with that previously reported^46^.

*5,7-Dihydroxy-2-(4-hydroxyphenyl)chroman-4-one* (**7b**)

5,7-Dihydroxy-2-(4-hydroxyphenyl)-*4H*-chromen-4-one (**6b**) was used as the starting material. Purification by flash column chromatography (eluting with DCM-E.A 5:1, v/v) provided **7b** in white solid (yield = 56%). R_f_ = 0.30 (DCM-E.A = 3:1, v/v). ^1^H NMR (300 MHz, (CD_3_)_2_CO) *δ* 12.19 (s, 1H), 9.52 (brs, 1H), 8.66 (brs, 1H), 7.40 (d, *J* = 8.4 Hz, 2H), 6.90 (d, *J* = 8.4 Hz, 2H), 5.95 (s, 2H), 5.45 (dd, *J* = 3.0 and 12.9 Hz, 1H), 3.19 (dd, *J* = 12.9 and 17.1 Hz, 1H), 2.73 (dd, *J* = 3.0 and 17.1 Hz, 1H). HRMS (ESI) *m/z* calculated for C_15_H_12_O_5_ [M − H]^−^: 271.0612; found: 271.0613. ^1^H NMR and HRMS data were in complete agreement with that previously reported^35^.

Brominated flavones (**8a–8c**) were synthesized by applying procedures for the synthesis of compound **5a** from appropriate brominated chalcones.

*2-(3-Bromophenyl)-5,7-dimethoxy-4H-chromen-4-one* (**8a**)

*(E)*-3-(3-Bromophenyl)-1-(2-hydroxy-4,6-dimethoxyphenyl)prop-2-en-1-one was used as the starting material. Purification by flash column chromatography (eluting with Toluene-E.A, 5:1 to 1:1, v/v) provided **8a** in white solid (yield = 94%). R_f_ = 0.28 (Toluene-E.A = 1:1, v/v). ^1^H NMR (300 MHz, CDCl_3_) *δ* 8.04 (t, *J* = 2.4 Hz, 1H), 7.75 (d, *J* = 7.8 Hz, 1H), 7.64 (dd, *J* = 1.5 and 7.2 Hz, 1H), 7.38 (t, *J* = 1.5, 1H), 6.66 (s, 1H), 6.59 (d, *J* = 2.1, 1H), 6.39 (d, *J* = 2.1, 1H), 3.97 (s, 3H), 3.93 (s, 3H). HRMS (ESI) *m/z* calculated for C_17_H_13_BrO_4_ [M + H]^+^: 361.0070; found: 361.0076. ^1^H NMR and HRMS data were in complete agreement with that previously reported^47^.

*2-(3-Bromophenyl)-6,7-dimethoxy-4H-chromen-4-one* (**8b**)

*(E)*-3-(3-Bromophenyl)-1-(2-hydroxy-4,5-dimethoxyphenyl)prop-2-en-1-one was used as the starting material. Purification by flash column chromatography (eluting with DCM-E.A, 10:1 to 4:1, v/v) provided **8b** in white solid (yield = 78%). R_f_ = 0.45 (DCM-E.A = 4:1, v/v). ^1^H NMR (300 MHz, CDCl_3_) *δ* 8.08 (s, 1H), 7.81 (d, *J* = 7.5 Hz, 1H), 7.66 (d, *J* = 8.7 Hz, 1H), 7.56 (s, 1H), 7.40 (t, *J* = 7.8 Hz, 1H), 7.03 (s, 1H), 6.78 (s, 1H), 4.04 (s, 3H), 4.00 (s, 3H); ^13^C NMR (75 MHz, CDCl_3_) *δ* 177.2, 160.7, 154.5, 152.1, 147.7, 134.1, 133.8, 130.4, 128.9, 124.5, 123.2, 117.2, 107.5, 104.1, 99.7, 56.5, 56.3. HRMS (ESI) *m/z* calculated for C_17_H_13_BrO_4_ [M + H]^+^: 361.0070; found: 361.0085.

*2-(4-Bromophenyl)-6,7-dimethoxy-4H-chromen-4-one* (**8c**)

*(E)*-3-(4-Bromophenyl)-1-(2-hydroxy-4,5-dimethoxyphenyl)prop-2-en-1-one was used as the starting material. Purification by flash column chromatography (eluting with CH_2_Cl_2_) provided **8c** in white solid (yield = 52%). R_f_ = 0.50 (DCM-E.A = 10:1, v/v). ^1^H NMR (300 MHz, CDCl_3_) *δ* 7.76 (d, *J* = 8.4 Hz, 2H), 7.65 (d, *J* = 8.4 Hz, 2H), 7.55 (s, 1H), 6.99 (s, 1H), 6.76 (s, 1H), 4.02 (s, 3H), 3.99 (s, 3H); ^13^C NMR (75 MHz, CDCl_3_) *δ* 177.4, 161.5, 154.5, 152.1, 147.7, 132.2, 130.8, 127.4, 125.9, 117.2, 107.1, 104.2, 99.7, 56.5, 56.3. HRMS (ESI) *m/z* calculated for C_17_H_13_BrO_4_ [M + H]^+^: 361.0070; found: 361.0085.

#### General procedure for the synthesis of biphenyl flavones (9a–9i)

To a solution of bromoflavones (**8a–8c**) in toluene was added appropriate benzeneboronic acid (1.2 eq) and tetrakis(triphenylphosphine)palladium (0.1 eq) under argon atmosphere. To the reaction mixture was added cesium carbonate (2 M solution, 10%). The reaction mixture was stirred at 90 °C for 5 h. After being cooled at room temperature, the reaction mixture was partitioned between ethyl acetate and water. The combined organic layer was collected, dried over magnesium sulfate, and concentrated under reduced pressure. The residue was purified by flash column chromatography to give biphenyl flavones (**9a–g**).

*5,7-Dimethoxy-2-(4*′*-methoxy-[1,1*′*-biphenyl]-3-yl)-4H-chromen-4-one* (**9a**)

2-(3-Bromophenyl)-5,7-dimethoxy-*4H*-chromen-4-one (**8a**) and 4-methoxybenzeneboronic acid were used as starting materials. Purification by flash column chromatography (eluting with hexane-acetone, 5:1 to 1:1, v/v) provided **9a** in white solid (yield = 50%). R_f_ = 0.21 (hexane-acetone = 2:1, v/v). ^1^H NMR (300 MHz, CDCl_3_) *δ* 8.02 (s, 1H), 7.79 (d, *J* = 7.8 Hz, 1H), 7.67 (d, *J* = 7.8 Hz, 1H), 7.50–7.59 (m, 3H), 7.02 (d, *J* = 8.7 Hz, 2H), 6.74 (s, 1H), 6.59 (d, *J* = 2.4 Hz, 1H), 6.38 (d, *J* = 2.4 Hz, 1H), 3.96 (s, 3H), 3.92 (s, 3H), 3.87 (s, 3H). ^13^C NMR (75 MHz, CDCl_3_) *δ *177.6, 162.6, 159.9, 154.5, 152.3, 147.7, 143.7, 132.2, 132.0, 131.9, 130.0, 128.6, 128.4, 128.2, 127.0, 126.5, 117.4, 114.4, 106.7, 104.4, 99.8, 56.4, 56.4, 55.4. HRMS (ESI) *m/z* calculated for C_24_H_20_O_5_ [M + H]^+^: 389.1384; found: 389.1419.

*5,7-Dimethoxy-2-(4*′*-nitro-[1,1*′*-biphenyl]-3-yl)-4H-chromen-4-one* (**9b**)

2-(3-Bromophenyl)-5,7-dimethoxy-*4H*-chromen-4-one (**8a**) and 4-nitrobenzeneboronic acid were used as starting materials. Purification by flash column chromatography (eluting with hexane-acetone, 5:1 to 1:1, v/v) provided **9b** in white solid (yield = 23%). R_f_ = 0.17 (hexane-acetone = 2:1, v/v). ^1^H NMR (300 MHz, CDCl_3_) *δ* 8.36 (d, *J* = 8.7 Hz, 2H), 8.10 (s, 1H), 7.94 (d, *J* = 8.1 Hz, 1H), 7.75–7.82 (m, 3H), 7.64 (t, *J* = 7.8 Hz, 1H), 6.76 (s, 1H), 6.61 (d, *J* = 2.4 Hz, 1H), 6.14 (d, *J* = 2.4 Hz, 1H), 3.98 (s, 3H), 3.93 (s, 3H).

*2-([1,1*′*-Biphenyl]-3-yl)-5,7-dimethoxy-4H-chromen-4-one* (**9c**)

2-(3-Bromophenyl)-5,7-dimethoxy-*4H*-chromen-4-one (**8a**) and phenylboronic acid were used as starting materials. Purification by flash column chromatography (eluting with petroleum ether -acetone, 4:1 to 1:1, v/v) provided **9c** in white solid (yield = 56%). R_f_ = 0.37 (petroleum ether-acetone = 4:1, v/v). ^1^H NMR (300 MHz, CDCl_3_) *δ* 8.05 (s, 1H), 7.81 (d, *J* = 7.8 Hz, 1H), 7.70 (d, *J* = 7.8 Hz, 1H), 7.62 (d, *J* = 7.2 Hz, 1H), 7.55 (d, *J* = 7.8 Hz, 1H), 7.53–7.44 (m, 2H), 7.43–7.35 (m, 1H), 6.73 (s, 1H), 6.57 (d, *J* = 2.1 Hz, 1H), 6.36 (d, *J* = 2.1 Hz, 1H), 3.94 (s, 3H), 3.90 (s, 3H); ^13^C NMR (75 MHz, CDCl_3_) *δ* 177.7, 164.2, 161.1, 160.7, 160.1, 142.2, 140.4, 132.2, 130.1, 129.5, 129.1, 128.0, 127.3, 124.9, 124.8, 109.4, 96.4, 93.0, 56.5, 55.9. HRMS (ESI) *m/z* calculated for C_23_H_18_O_4_ [M + H]^+^: 359.1278; HRMS (ESI) *m/z* calculated for C_23_H_18_O_4_[M + Na]^+^: 381.1097; found: 381.1095.

*2-(4*′*-Fluoro-[1,1*′*-biphenyl]-3-yl)-5,7-dimethoxy-4H-chromen-4-one* (**9d**)

2-(3-Bromophenyl)-5,7-dimethoxy-*4H*-chromen-4-one (**8a**) and 4-fluorobenzeneboronic acid were used as starting materials. Purification by flash column chromatography (eluting with petroleum ether -acetone, 4:1 to 1:1, v/v) provided **9d** in white solid (yield = 91%). R_f_ = 0.30 (petroleum ether-acetone = 3:1, v/v). ^1^H NMR (300 MHz, CDCl_3_) *δ* 7.99 (s, 1H), 7.81 (d, *J* = 7.8 Hz, 1H), 7.65 (d, *J* = 7.8 Hz, 1H), 7.63–7.50 (m, 3H), 7.17 (t, *J* = 8.4 Hz, 2H), 6.72 (s, 1H), 6.58 (d, *J* = 2.4 Hz, 1H), 6.37 (d, *J* = 2.4 Hz, 1H), 3.95 (s, 3H), 3.91 (s, 3H); ^13^C NMR (75 MHz, CDCl_3_) *δ* 177.6, 164.3, 164.2, 161.1, 161.0, 160.5, 160.0, 141.2, 136.5, 136.4,132.3, 129.8, 129.6, 129.0, 128.9, 124.9, 124.6, 116.1, 115.8, 109.4, 96.37, 93.0, 56.5, 55.9. HRMS (ESI) *m/z* calculated for C_23_H_17_FO_4 _[M + Na]^+^: 399.1003; found: 399.1001.

*2-([1,1*′*-Biphenyl]-3-yl)-6,7-dimethoxy-4H-chromen-4-one* (**9e**)

2-(3-Bromophenyl)-6,7-dimethoxy-*4H*-chromen-4-one (**8b**) and benzeneboronic acid were used as starting materials. Purification by flash column chromatography (eluting with petroleum ether-acetone, 10:1 to 3:1, v/v) provided **9e** in white solid (yield = 63%). R_f_ = 0.38 (petroleum ether-acetone = 4:1, v/v). ^1^H NMR (300 MHz, CDCl_3_) *δ* 8.12 (s, 1H), 7.89 (d, *J* = 7.2 Hz, 1H), 7.75 (d, *J* = 7.8 Hz, 1H), 7.59–7.67 (m, 4H), 7.51 (t, *J* = 6.9, 2H), 7.43 (d, *J* = 7.5 Hz, 1H), 7.03 (s, 1H), 6.87 (s, 1H), 4.04 (s, 3H), 4.01 (s, 3H); ^13^C NMR (75 MHz, CDCl_3_) *δ* 177.5, 162.5, 154.5, 152.2, 147.7, 142.1, 140.1, 132.4, 132.2, 132.0, 131.9, 131.9, 130.0, 129.4, 129.0, 128.6, 128.4, 127.9, 127.2, 124.8, 124.7, 117.3, 107.2, 104.3, 99.9, 56.9, 55.9. HRMS (ESI) *m/z* calculated for C_23_H_18_O_4_ [M + H]^+^: 359.1278; found: 359.1294.

*2-(4*′*-Fluoro-[1,1*′*-biphenyl]-3-yl)-6,7-dimethoxy-4H-chromen-4-one* (**9f**)

2-(3-Bromophenyl)-6,7-dimethoxy-*4H*-chromen-4-one (**8b**) and 4-fluorobenzene boronic acid were used as starting materials. Purification by flash column chromatography (eluting with petroleum ether-acetone, 10:1 to 3:1, v/v) provided **9f** in white solid (yield = 55%). R_f_ = 0.31 (petroleum ether-Acetone = 3:1, v/v). ^1^H NMR (300 MHz, CDCl_3_) *δ* 8.06 (s, 1H), 7.87 (d, *J* = 7.8 Hz, 1H), 7.70 (d, *J* = 7.8 Hz, 1H), 7.59–7.63 (m, 4H), 7.19 (t, *J* = 8.7, 2H), 7.03 (s, 1H), 6.86 (s, 1H), 4.04 (s, 3H), 4.00 (s, 3H); ^13^C NMR (75 MHz, CDCl_3_) *δ* 177.6, 162.6, 154.6, 152.4, 147.8, 141.2, 132.6, 132.3, 132.1, 132.1, 132.0, 129.9, 129.6. 129.0, 128.9, 128.7, 128.5, 125.0, 124.7, 117.5, 116.1, 115.8, 107.4, 104.5, 99.9, 56.9, 55.9. HRMS (ESI) *m/z* calculated for C_23_H_17_FO_4_ [M + H]^+^: 377.1184; found: 377.1196.

*6,7-Dimethoxy-2-(4*′*-methoxy-[1,1*′*-biphenyl]-3-yl)-4H-chromen-4-one* (**9g**)

2-(3-Bromophenyl)-6,7-dimethoxy-*4H*-chromen-4-one (**8b**) and 4-methoxybenzeneboronic acid were used as starting materials. Purification by flash column chromatography (eluting with petroleum ether-acetone, 10:1 to 3:1, v/v) provided **9g** in white solid (yield = 78%). R_f_ = 0.35 (petroleum ether-acetone = 3:1, v/v). ^1^H NMR (300 MHz, CDCl_3_) *δ* 8.08 (s, 1H), 7.83 (d, *J* = 7.5 Hz, 1H), 7.71 (d, *J* = 7.8 Hz, 1H), 7.54–7.61 (m, 4H), 7.05 (s, 1H), 7.03 (s, 2H), 6.86 (s, 1H), 4.04 (s, 3H), 4.01 (s, 3H), 3.88 (s, 3H); ^13^C NMR (75 MHz, CDCl_3_) *δ* 177.7, 162.9, 159.8, 154.7, 152.5, 147.9, 141.9, 132.8, 132.6, 132.3, 132.2, 132.0, 129.8, 129.5, 128.7, 128.5, 128.4, 124.5, 124.4, 117.5, 114.6, 56.6, 56.5, 55.5. HRMS (ESI) *m/z* calculated for C_24_H_20_O_5_ [M + H]^+^: 389.1384; found: 389.1407.

*6,7-Dimethoxy-2-(4*′*-nitro-[1,1*′*-biphenyl]-3-yl)-4H-chromen-4-one* (**9h**)

2-(3-Bromophenyl)-6,7-dimethoxy-*4H*-chromen-4-one (**8b**) and 4-nitrobenzeneboronic acid were used as starting materials. Purification by flash column chromatography (eluting with Petroleum ether-Acetone, 5:1 to 1:1, v/v) provided **9h** in white solid (yield = 24%). R_f_ = 0.25 (Petroleum ether-Acetone = 3:1, v/v). ^1^H NMR (300 MHz, CDCl_3_) *δ* 8.37 (d, *J* = 8.4 Hz, 2H), 8.14 (s, 1H), 7.98 (d, *J* = 7.8 Hz, 1H), 7.77–7.83 (m, 3H), 7.67 (t, *J* = 7.5 Hz, 1H), 7.59 (s, 1H), 7.04 (s, 1H), 6.88 (s, 1H), 4.04 (s, 3H), 4.01 (s, 3H).

*6,7-Dimethoxy-2-(4*′*-methoxy-[1,1*′*-biphenyl]-4-yl)-4H-chromen-4-one* (**9i**)

2-(3-Bromophenyl)-5,7-dimethoxy-*4H*-chromen-4-one (**8c**) and 4-methoxybenzeneboronic acid were used as starting materials. Purification by flash column chromatography (eluting with Toluene-E.A, 10:1 to 3:1, v/v) provided **9i** in white solid (yield = 63%). R_f_ = 0.20 (Hexane-E.A = 3:1, v/v). ^1^H NMR (300 MHz, CDCl_3_) *δ* 7.96 (d, *J* = 8.4 Hz, 2H), 7.71 (d, *J* = 8.4 Hz, 2H), 7.62 (s, 1H), 7.59 (d, *J* = 2.4 Hz, 2H), 7.01–7.04 (m, 3H), 6.83 (s, 1H), 4.04 (s, 3H), 4.01 (s, 3H), 3.88 (s, 3H). HRMS (ESI) *m/z* calculated for C_24_H_20_O_5_ [M + H]^+^: 389.1384; found: 389.1408.

#### General procedure for the synthesis of compounds (10a–10i)

The ring-extended flavone compounds were dissolved in anhydrous dichloromethane and was added boron tribromide (1 M solution, 5 eq per methoxy functional group) at 0 °C under argon atmosphere. The reaction mixture was stirred under reflux for 12 h. After being cooled to room temperature, the reaction mixture was concentrated under reduced pressure and was diluted with ethyl acetate and water. The combined water layer was adjusted to pH 7, and re-partitioned with ethyl acetate. The organic layer was collected, dried over magnesium sulfate, and concentrated under reduced pressure. The residue was purified by flash column chromatography to give final compounds (**10a–10i**).

*5,7-Dihydroxy-2-(4*′*-hydroxy-[1,1*′*-biphenyl]-3-yl)-4H-chromen-4-one* (**10a**)

5,7-Dimethoxy-2-(4′-methoxy-[1,1′-biphenyl]-3-yl)-*4H*-chromen-4-one (**9a**) was used as the starting material. Purification by flash column chromatography (eluting with DCM-MeOH, 10:1 to 3:1, v/v) provided **10a** in white solid (yield = 38%). R_f_ = 0.40 (DCM-MeOH = 10:1, v/v). ^1^H NMR (300 MHz, tetrahydrofuran-*d*_8_) *δ* 12.86 (brs, 1H), 8.15 (s, 1H), 7.87 (d, *J* = 7.5 Hz, 1H), 7.73 (d, *J* = 7.8 Hz, 1H), 7.55 (d, *J* = 7.8 Hz, 2H), 7.52 (d, *J* = 8.1 Hz, 2H), 6.85 (d, *J* = 7.8 Hz, 3H), 6.43 (s, 1H), 6.17 (s, 1H); ^13^C NMR (75 MHz, tetrahydrofuran-*d*_8_) *δ* 182.8,165.3, 164.4, 163.5, 158.8, 158.8, 142.9, 132.9, 131.7, 130.1, 129.9, 128.7, 124.7, 116.4, 106.3, 105.4, 99.6, 94.4. HRMS (ESI) *m/z* calculated for C_21_H_14_O_4_ [M − H]^−^: 345.0768; found: 345.0769.

*5,7-Dihydroxy-2-(4*′*-nitro-[1,1*′*-biphenyl]-3-yl)-4H-chromen-4-one* (**10b**)

5,7-Dimethoxy-2-(4′-nitro-[1,1′-biphenyl]-3-yl)-*4H*-chromen-4-one (**9b**) was used as the starting material. Purification by flash column chromatography (eluting with DCM-MeOH, 30:1 to 10:1, v/v) provided **10b** in white solid (yield = 13%). R_f_ = 0.60 (DCM-MeOH = 10:1, v/v). ^1^H NMR (300 MHz, tetrahydrofuran-*d*_8_) *δ* 12.81 (s, 1H), 9.64 (brs, 1H), 8.35 (d, *J* = 8.4 Hz, 1H), 8.09 (d, *J* = 8.1 Hz, 1H), 8.01 (d, *J* = 8.7 Hz, 2H), 7.94 (d, *J* = 8.4 Hz, 1H), 7.69 (t, *J* = 7.8 Hz, 1H), 6.93 (s, 1H), 6.45 (d, *J* = 2.1 Hz, 1H), 6.19 (d, *J* = 2.1 Hz, 1H); ^13^C NMR (75 MHz, tetrahydrofuran-*d*_8_) *δ* 182.9, 165.6, 163.8, 163.7, 159.0, 148.7, 147.1, 140.7, 133.6, 131.3, 130.7, 129.0, 127.4, 126.1, 124.8, 106.9, 105.6, 99.9, 94.6, 30.6. HRMS (ESI) *m/z* calculated for C_21_H_13_NO_6_ [M − H]^−^: 374.0670; found: 374.0685.

*2-([1,1*′*-Biphenyl]-3-yl)-5,7-dihydroxy-4H-chromen-4-one* (**10c**)

2-([1,1′-Biphenyl]-3-yl)-5,7-dimethoxy-*4H*-chromen-4-one (**9c**) was used as the starting material. Purification by flash column chromatography (eluting with DCM-MeOH, 50:1 to 10:1, v/v) provided **10c** in white solid (yield = 54%). R_f_ = 0.62 (DCM-MeOH = 10:1, v/v). ^1^H NMR (300 MHz, tetrahydrofuran-*d*_8_) *δ* 12.85 (brs, 1H), 8.24 (s, 1H), 7.97 (d, *J* = 7.5 Hz, 1H), 7.81 (d, *J* = 7.5 Hz, 1H), 7.61 (d, *J* = 6.9 Hz, 2H), 7.60 (t, *J* = 7.8 Hz, 1H), 7.46 (t, *J* = 6.9 Hz, 2H), 7.36 (t, *J* = 7.2 Hz, 1H), 6.87 (s, 1H), 6.45 (s, 1H), 6.19 (s, 1H); ^13^C NMR (75 MHz, tetrahydrofuran-*d*_8_) *δ* 182.8, 165.4, 164.1, 163.5, 158.8, 142.9, 140.9, 133.1, 130.8, 130.1, 129.5, 128.5, 127.8, 125.8, 125.5, 106.5, 105.4, 99.7, 94.4. HRMS (ESI) *m/z* calculated for C_21_H_14_O_4 _[M − H]^−^: 329.0819; found: 329.0819.

*2-(4*′*-Fluoro-[1,1*′*-biphenyl]-3-yl)-5,7-dihydroxy-4H-chromen-4-one* (**10d**)

2-(4′-Fluoro-[1,1′-biphenyl]-3-yl)-5,7-dimethoxy-*4H*-chromen-4-one (**9d**) was used as the starting material. Purification by flash column chromatography (DCM-MeOH, 50:1 to 10:1, v/v) provided **10d** in white solid (yield = 51%). R_f_ = 0.59 (DCM-MeOH = 10:1, v/v). ^1^H NMR (300 MHz, tetrahydrofuran-*d*_8_) *δ* 12.82 (s, 1H), 9.80 (brs, 1H), 8.20 (s, 1H), 7.96 (d, *J* = 7.0 Hz, 1H), 7.78–7.71 (m, 3H), 7.62–7.55 (m, 3H), 7.24–7.17 (m, 2H), 6.86 (s, 1H), 6.44 (s, 1H), 6.19 (s, 1H); ^13^C NMR (75 MHz, tetrahydrofuran-*d*_8_) *δ* 183.0, 165.6, 164.2, 163.7, 159.0, 142.0, 137.3, 137.3, 133.3, 130.8, 130.4, 129.9, 129.8, 125.9, 125.5, 116.6, 116.3, 106.7, 105.6, 99.9, 94.6. HRMS (ESI) *m/z* calculated for C_21_H_13_FO_4_ [M − H]^−^: 347.0725; found: 347.0729.

*2-([1,1*′*-Biphenyl]-3-yl)-6,7-dihydroxy-4H-chromen-4-one* (**10e**)

2-([1,1′-Biphenyl]-3-yl)-6,7-dimethoxy-*4H*-chromen-4-one (**9e**) was used as the starting material. Purification by flash column chromatography (DCM-MeOH, 30:1 to 10:1, v/v) provided **10e** in white solid (yield = 46%). R_f_ = 0.21 (DCM-MeOH = 10:1, v/v). ^1^H NMR (300 MHz, tetrahydrofuran-*d*_8_) *δ* 8.26 (brs, 1H), 9.12 (brs, 1H), 8.22 (s, 1H), 7.94 (d, *J* = 7.8 Hz, 1H), 7.76 (d, *J* = 7.8 Hz, 1H), 7.71 (d, *J* = 7.2 Hz, 2H), 7.57 (t, *J* = 7.8, 1H), 7.48–7.41 (m, 3H),7.35 (t, *J* = 7.2 Hz, 1H), 7.00 (s, 1H), 6.82 (s, 1H); ^13^C NMR (75 MHz, tetrahydrofuran-*d*_8_) *δ* 176.9, 162.4, 153.1, 152.4, 145.0, 142.6, 141.0, 133.9, 130.0, 129.9, 129.4, 128.2, 127.7, 125.4, 125.1, 117.8, 108.5, 107.2, 103.5. HRMS (ESI) *m/z* calculated for C_21_H_14_O_4_ [M − H]^−^: 329.0819; found: 329.0829.

*2-(4*′*-Fluoro-[1,1*′*-biphenyl]-3-yl)-6,7-dihydroxy-4H-chromen-4-one* (**10f**)

2-(4′-Fluoro-[1,1′-biphenyl]-3-yl)-6,7-dimethoxy-*4H*-chromen-4-one (**9f**) was used as the starting material. Purification by flash column chromatography (eluting with DCM-MeOH, 30:1 to 10:1, v/v) provided **10f** in white solid (yield = 33%). R_f_ = 0.38 (DCM-MeOH = 10:1, v/v). ^1^H NMR (300 MHz, tetrahydrofuran-*d*_8_) *δ* 9.13 (brs, 1H), 8.98 (brs, 1H), 8.02 (s, 1H), 7.95 (d, *J* = 7.8 Hz, 1H), 7.77 (d, *J* = 8.4 Hz, 1H), 7.75 (d, *J* = 8.7 Hz, 1H), 7.58 (t, *J* = 7.8, 1H), 7.39 (s, 1H), 7.21 (t, *J* = 8.7, 2H), 6.97 (s, 1H), 6.80 (s, 1H); ^13^C NMR (75 MHz, tetrahydrofuran-*d*_8_) *δ* 176.6, 162.2, 153.0, 152.4, 144.9, 141.6, 137.4, 134.1, 130.0, 129.9, 129.7, 129.6, 125.5, 125.1, 118.0, 116.4, 116.1, 108.6, 107.4, 103.6, 30.5. HRMS (ESI) *m/z* calculated for C_21_H_13_FO_4_ [M − H]^−^: 347.0725; found: 347.0741.

*6,7-Dihydroxy-2-(4*′*-hydroxy-[1,1*′*-biphenyl]-3-yl)-4H-chromen-4-one* (**10g**)

6,7-Dimethoxy-2-(4′-methoxy-[1,1′-biphenyl]-3-yl)-*4H*-chromen-4-one (**9g**) was used as the starting material. Purification by flash column chromatography (eluting with DCM-MeOH, 30:1 to 10:1, v/v) provided **10g** in white solid (yield = 25%). R_f_ = 0.25 (DCM-MeOH = 10:1, v/v). ^1^H NMR (300 MHz, CD_3_OD) *δ* 8.13 (s, 1H), 7.90 (d, *J* = 7.8 Hz, 1H), 7.76 (d, *J* = 6.6 Hz, 1H), 7.55–7.61 (m, 3H), 7.43 (s, 1H), 7.08 (s, 1H), 6.91 (d, *J* = 8.4, 2H), 6.86 (s, 1H). HRMS (ESI) *m/z* calculated for C_21_H_14_O_4_ [M − H]^−^: 345.0768; found: 345.0757.

*6,7-Dihydroxy-2-(4*′*-nitro-[1,1*′*-biphenyl]-3-yl)-4H-chromen-4-one* (**10h**)

6,7-Dimethoxy-2-(4′-nitro-[1,1′-biphenyl]-3-yl)-*4H*-chromen-4-one (**9h**) was used as the starting material. Purification by flash column chromatography (eluting with DCM-MeOH, 30:1 to 10:1, v/v) provided **10h** in white solid (yield = 30%). R_f_ = 0.50 (DCM-MeOH = 10:1, v/v). ^1^H NMR (300 MHz, CD_3_OD) *δ* 8.46 (s, 1H), 8.40 (d, *J* = 8.7 Hz, 2H), 8.13–8.16 (m, 3H), 8.01 (d, *J* = 8.4 Hz, 1H), 7.77 (t, *J* = 7.8 Hz, 1H), 7.51 (s, 1H), 7.20 (s, 1H), 6.90 (s, 1H). HRMS (ESI) *m/z* calculated for C_21_H_13_NO_6_ [M − H]^−^: 374.0670; found: 374.0686.

*6,7-Dihydroxy-2-(4*′*-hydroxy-[1,1*′*-biphenyl]-4-yl)-4H-chromen-4-one* (**10i**)

6,7-Dimethoxy-2-(4′-methoxy-[1,1′-biphenyl]-4-yl)-*4H*-chromen-4-one (**9i**) was used as the starting material. Purification by flash column chromatography (eluting with DCM-MeOH, 30:1 to 10:1, v/v) provided **10i** in white solid (yield = 33%). R_f_ = 0.50 (DCM-MeOH = 10:1, v/v). ^1^H NMR (300 MHz, CD_3_OD) *δ* 8.09 (d, *J* = 8.4 Hz, 2H), 7.80 (d, *J* = 8.4 Hz, 2H), 7.64 (d, *J* = 8.7 Hz, 2H), 7.49 (s, 1H), 7.16 (s, 1H), 6.98 (d, *J* = 8.7 Hz, 2H), 6.75 (s, 1H). HRMS (ESI) *m/z* calculated for C_21_H_14_O_4_ [M − H]^−^: 345.0768; found: 345.0765.

*5,7-Dihydroxy-2-(4*′*-hydroxy-[1,1*′*-biphenyl]-3-yl)chroman-4-one* (**11a**)

A mixture of compound **10a** (43 mg, 0.12 mmol) and 10% Pd/C (30 mg) in 5 mL of 1,4-dioxane-EtOH (1/4, v/v) was hydrogenated on a Parr shaker apparatus under 50 psi H_2_ for 23 h. After filtration, the solvent was evaporated under reduced pressure, and the residue was purified by silica gel column chromatography (eluting with DCM-MeOH, 50:1 to 10:1, v/v) to give **11a** (68 mg, 68%) as colorless oil. R_f_ = 0.71 (DCM-MeOH = 10:1, v/v). ^1^H NMR (300 MHz, CD_3_OD) *δ* 7.47 (s, 1H), 7.53 (d, *J* = 7.5 Hz, 1H), 7.44 (d, *J* = 8.7 Hz, 2H), 7.40 (d, *J* = 7.8 Hz, 1H), 7.35 (t, *J* = 6.0 Hz, 1H), 6.86 (d, *J* = 8.7 Hz, 2H), 5.93 (dd, *J* = 2.2 and 14.4 Hz, 2H), 5.46 (dd, *J* = 3.0 and 12.3 Hz, 1H), 3.11 (dd, *J* = 12.8 and 17.1 Hz, 1H), 2.78 (dd, *J* = 3.1 and 17.1 Hz, 1H); ^13^C NMR (75 MHz, CD_3_OD) *δ* 197.3, 168.4, 165.4, 164.6, 158.4, 142.8, 140.8, 133.3, 130.1, 129.1, 128.6, 127.6, 125.4, 125.3, 116.7, 116.1, 103.4, 97.2, 96.3, 80.5, 44.2. HRMS (ESI) *m/z* calculated for C_21_H_16_O_5_ [M − H]^−^: 347.0925; found: 347.0927.

### Measurement of kinetic aqueous solubility

Measurement of kinetic aqueous solubility was conducted using syringeless filter device (Whatman UniPrep Syringeless Filter Device, 1 mL, PTFE membrane, 0.45 μm pore size, Cat. No. US113UORG) and high performance liquid chromatography (Thermo UltiMate 3000 HPLC system equipped with on-line degasser, quaternary pump, thermostatted auto-sampler, column compartment and diode array detector) device and Agilent Eclipse plus C18, 4.6 × 100 mm, 3.5 μm column. In brief, 10 mM stock solution of tested compounds was prepared in DMSO. 25 μL of the stock solution was added to a 1-mL uniprep vial containing 475 μL of 0.1 M potassium phosphate buffer (pH 7.4), and was shaken at 800 rpm for 90 min at room temperature. Following shaking, the mixture was filtered by slowly pressing the upper tube of the 1 mL Mini-Uniprep^TM^ filter (PTFE membrane, 0.45 μm). 250 μL of the filtrate solution was then transferred to a HPLC vial containing equal volume of acetonitrile. Calibration samples for HPLC analysis were prepared using 10 mM DMSO stock solutions in 50 v/v% acetonitrile in deionized water or phosphate buffer (nominal concentrations: 500, 100, 20, 5, 1 and 0 μM. Diluted filtrate solutions and calibration samples were analyzed by HPLC. The solubility of the test samples was calculated by multiplying the measured concentration with the dilution factor.

### Protein preparation using baculovirus expression vector system

The genes endoding FLAG (a peptide DYKDDDDK sequence motif)-tagged human TSLP_28–159_ with thrombin cleavage site at C-terminus (FLAG-hTSLP-thrombin) and human TSLPR_26–217_ with C-terminal octa-histidine tag (hTSLPR-His), were cloned into the pAB-bee-8 × His vector (AB vector, USA) to construct the plasmid transfer vectors, and the vectors were amplified in *E. coli* DH5α. The recombinant baculoviruses were generated by co-transfection of the plasmid transfer vectors and the linearized baculovirus genomic vector ProFold-ER1 (AB vector, USA) into the *Spodoptera frugiperda* (Sf-9) cells, (Invitrogen, USA). The recombinant baculoviruses were amplified in Sf-9 cells cultured in Sf-900 II SFM medium (Thermo Fisher Scientific Inc, USA). Thereafter, *Trichoplusiani* (High-Five cells) (Invitrogen, USA), cultured in ESF921 medium (Expression Systems, LLC, USA), were infected with the recombinant baculoviruses harboring hTSLP or hTSLPR gene to produce hTSLP or hTSLPR protein, respectively, which was secreted into the medium. The resulting proteins were loaded into a Ni-NTA HisTrap column (Qiagen, Germany) pre-equilibrated with buffer A (20 mM Tris-Cl, 200 mM NaCl at pH 8.0) and eluted with buffer B (20 mM Tris-Cl, 200 mM NaCl, 1 M Imidazole at pH 8.0). The octa-histidine tag was cleaved during dialysis by overnight incubation with thrombin in buffer A at 4 °C. After cleavage, proteins were concentrated by ultrafiltration (10,000 MWCO; Merck Millipore, Germany) and loaded onto a Superdex S75 gel-filtration column (16 mm/60 cm; GE healthcare, UK) pre-equilibrated with 20 mM Tris-Cl, 200 mM NaCl, and 1 mM dithiothreitol at pH 8.0.

### Protein Preparation for 2D NMR studies

$TSL{P}_{(29-159{\rm{\Delta }}127-131)}$ was cloned in an expression vector, pET28a (Novagen), as an N-terminal His-tag fusion protein in *E. coli* BL21(DE3). To obtain a uniform labeled ^15^N $TSL{P}_{(29-159{\rm{\Delta }}127-131)}$, bacterial cells were grown in M9 minimal medium containing ^15^N NH_4_Cl. Cells were further grown for 4 h at 37 °C after induction by 1M IPTG (isopropyl-β-D-thiogalactoside) when cell density (OD_600_) reached 0.6. After harvested, cells were resuspended in lysis buffer (0.1M Tris pH 7.4, 0.3M sodium chloride, 1 mM β-mercaptoethanol, 0.1% TritonX100, and 0.1 mM phenylmethylsulfonyl fluoride). Cells were then disrupted by sonication in an ice bath, and centrifuged at 32,500 × *g* for 60 min at 4 °C. The pellet, containing the inclusion bodies, was re-suspended in 8 M urea, 50 mM sodium phosphate pH 6.8, 5 mM β-mercaptoethanol and sonicated as above. Insoluble material was removed by high speed centrifugation at 32,500 × *g* for 60 min at 4 °C. The supernatant was loaded into 5 mL of pre-equilibrated Ni-NTA (HisTrap; GE Healthcare) column with buffer A (8 M urea, 50 mM sodium phosphate pH 6.8, and 5 mM β-mercaptoethanol). $TSL{P}_{(29-159{\rm{\Delta }}127-131)}$ was eluted out of the column by using buffer A containing 500 mM Imidazole at pH 4.5. Eluted inclusion bodies solution was added drop-wise into refolding buffer (100 mM Tris pH 7.4, 1M L-arginine, 5 mM reduced glutathione, 0.5 mM oxidized glutathione, and 0.2 mM phenylmethylsulfonyl fluoride) at 4 °C with rapid stirring. The final concentration of protein was less than 0.1 mg/mL. The hexa-histidine tag was cleaved by thrombin protease at 4 °C in dialysis buffer (20 mM Tris pH 7.4, 300 mM sodium chloride) for overnight. Refolded proteins were further purified by size-exclusion chromatography using a Superdex 75 column with 20 mM sodium phosphate pH 6.8, 50 mM sodium chloride, and 2.5 mM ethylenediaminetetraacetic acid).

### NMR binding study

All NMR measurements were performed using an Avance 600 MHz NMR spectrometer equipped with a triple-resonance, pulsed field gradient probe (Bruker, Germany). All spectra were measured at 283, 291, and 298 K. Data processing and analysis were conducted using TopSpin 3.1 program (Bruker, Germany). One-dimensional (1D) relaxation-edited experiments were performed using the method previously described by Hadjuk *et al*.^23^. We used CPMG pulse train with a pre-acquisition delay of 1.8 s, a 2 ms delay for dephasing and rephrasing in spin echo, and a total spin-lock time of 400 ms. The data were collected using a spectral width of 9,615 Hz and 128 scans. We monitored the aromatic signals of **1** (100 µM) in the absence or presence of 1.25, 2.5, 5, or 10 μM hTSLP, 2.5 μM hTSLPR or 2.5 μM carboxy anhydrase in a buffer containing 20 mM sodium phosphate buffer (pH 7.4), 50 mM NaCl, and 1% deuterated DMSO at 298 K.

In order to investigate the ligand-binding site on hTSLP, sequence-specific assignments for hTSLP were completed through 2D ^1^H-^15^N HSQC, 3D HNCA, 3D HNCACB, 3D CBCA(CO)NH, 3D ^15^N-NOESY HSQC, and 3D ^13^C-NOESY HSQC experiments in a buffer containing 20 mM sodium phosphate (pH 5.5), 50 mM sodium chloride, 2.5 mM ethylenediaminetetraacetic acid. Then, series of 2D ^1^H-^15^N HSQC spectra of $TSL{P}_{29-159{\rm{\Delta }}127-131}$ were measured in the absence and presence of **1** in same molar ratio in a buffer containing 20 mM sodium phosphate (pH 6.8), 50 mM sodium chloride, 2.5 mM ethylenediaminetetraacetic acid, and 1.25% dimethyl sulfoxide. Data were processed with NMRpipe^27^ and analyzed with CCPN2.1.5^28^. Chemical shift perturbations of ^15^N and ^1^H nuclei were analyzed by overlaying the ^15^N-labelled free protein with **1** in different molar ratio. The magnitude of the combined ^1^H-^15^N chemical shift differences (Δδ, ppm) were calculated using the equation Δδ = [(δH^2^) + 0.2 × (δN^2^)]^1/2^, where δH and δN are changes to the proton (^1^H) and nitrogen (^15^N) chemical shifts, respectively^29^.

To estimate the equilibrium dissociation constant (*K*_*d*_) value, a series of ^1^H NMR experiments were conducted as described previously^22^. The linewidths of the H3 signals at various concentrations of **1** (50, 70, 100, 130, 170, 230, and 300 μM) in the presence of 5 µM hTSLP were monitored in a buffer containing 20 mM Tris-Cl (pH 8.0), 50 mM NaCl, and 1% deuterated DMSO at 298 K. The data were collected using a spectral width of 9,615 Hz and 384 scans. The equation, $1/({T}_{obs}-{T}_{free})$, *versus* concentration of **1** was plotted and the *K*_*d*_ value was calculated from the slope and Y intercept.

### Hydrogen/deuterium exchange mass spectrometry (HDX-MS)

hTSLP was de-glycosylated with PNGaseF prepared at a concentration of 100 µM in 20 mM Tris and 150 mM NaCl (pH 8.0). Ligand-binding was performed by incubating compound **1** (500 µM) with 100 µM hTSLP for 30 min at room temperature. Hydrogen/deuterium exchange was initiated by mixing 4 µL of hTSLP with 26 µL of D_2_O buffer (20 mM Tris at pH 8.0, 150 mM NaCl in D_2_O, and 50 µM of compound **1**) and incubated for 10, 100, 1,000, or 10,000 s on ice. The mixtures were quenched at the indicated time points by adding 30 µL of ice-cold quench buffer (100 mM NaH_2_PO_4_ at pH 2.01). For non-deuterated samples (ND), 4 µL of purified protein was mixed with 26 µL of H_2_O buffer (20 mM Tris at pH 8.0 and 150 mM NaCl in H_2_O) and quenched with 30 µL of ice-cold quench buffer. The quenched samples were digested online by passing through an immobilized pepsin column, and the peptide masses were analyzed as described previously^48^. Peptides in ND samples were identified with ProteinLynx Global Server (PLGS) 2.4 (Waters, Milford, MA, USA). The following parameters were applied: monoisotopic mass, non-specific for the enzyme while allowing up to one missed cleavage, MS/MS ion searches, automatic fragment mass tolerance, and automatic peptide mass tolerance. The variable modification used in all searches was methionine oxidation, and the peptides were filtered based on a peptide score of six. To process HDX-MS data, the amount of deuterium in each peptide was determined by measuring the centroid of the isotopic distribution using DynamX 2.0 (Waters, Milford, MA, USA). The average back-exchange level in our system was about 30%. However, back-exchange corrections were not made because analyses were performed by comparing **1**-bound with unbound hTSLP. All data were derived from three independent experiments, and Student’s *t*-test was employed for statistical analyses.

### Docking simulations

Human TSLP structure (PDB ID: 5J11) was prepared using Gasteiger charge, protein structure was kept rigid in docking, and binding site of compound **1** was defined from CSP-mapped residues obtained from NMR binding experiments. Grid dimensions with 60 × 48 × 60 points and 0.375 Å grid spacing were used for sampling of ligand conformations in the CSP based binding site. Compound **1** was modeled with SYBYL-X 2.0 molecular modeling package (http://tripos.com), and energy minimized with Gasteiger-Hückel charge set in vacuum dielectric environment, using Powell algorithm and Tripos force field for 5000 iterations subject to a termination gradient of 0.05 kcal/(mol·Å). Energy minimized **1** and its derivatives were prepared for Autodock, Gasteiger charges were assigned to chemicals. Autodock4.2 (http://autodock.scripps.edu/)^31^ was used to sample 200 docking poses. Ligand conformations were sampled by Lamarckian genetic algorithm, parameters were set as 200 independent runs, an initial population of 150 randomly placed individuals, with 2.5 × 10^6^ energy evaluations, a maximum number of 27000 iterations, a mutation rate of 0.02, a crossover rate of 0.80, and an elitism value of 1. Docking poses from the most populated cluster that are the low energy poses were selected for analysis. Pymol (http://www.pymol.org) was used for manual inspection of distances.

### ELISA assay

ELISA was performed using Ni-NTA HisSorb plates (Qiagen, Germany). In brief, 100 µL of a solution containing hTSLPR with C-terminal octa-histidine tag (TSLPR-His) was added to each well and incubated for 2 h at room temperature. After incubation, the plate was washed twice with 200 µL of PBS with 0.05% Tween-20 to remove unbound TSLPR-His, and candidate inhibitors as well as TSLP with N-terminal FLAG tag (FLAG-hTSLP) were added at 100 µL each. After overnight (18 h) incubation at 4 °C, the plate was washed twice and blocked with 100 µL of blocking buffer (PBS with 0.05% Tween 20 and 1% nonfat dry milk). The plate was washed twice to remove unbound FLAG-hTSLP and then coated with 100 µL of monoclonal anti-FLAG antibody conjugated to HRP (Sigma-Aldrich Co., USA) for 2 h at room temperature. Following incubation, the plate was washed five times and further treated with 200 µL of *o*-phenylenediamine dihydrochloride (Sigma-Aldrich Co., USA) solution and incubated for 30 min. After incubation, 1N HCl was added to stop the reaction. Optical densities (ODs) were measured at 450 nm using a microplate spectrophotometer. The TSLP-inhibitory effect was calculated using the following formula:

$${\rm{Inhibitory}}\,{\rm{effect}}\,( \% )=(1-{\rm{OD}}\,{\rm{of}}\,\mathrm{sample}/\mathrm{OD}\,{\rm{of}}\,{\rm{control}})\times 100$$

### STAT5 assay

#### Cell culture

HMC-1 cells were obtained from the Department of Food Technology and Inflammatory Disease Research Center (Hoseo University, Asan, Chungnam, Korea). Cells were cultured in Iscove’s modified Dulbecco’s medium (IMDM; Hyclone Laboratories Inc, USA) supplemented with 10% FBS and 1% Penicillin-streptomycin (PS; Gibco Industries Inc, USA) and incubated at 37 °C under humidified atmosphere of 95% air and 5% CO_2_. Recombinant hTSLP was purchased from R&D Systems (Minneapolis, USA).

#### Flow cytometry

Intracellular phospho-STAT5 (pSTAT5) staining method was based on the protocol obtained from the laboratory of Susan Kaech, Department of Immunobiology, Yale University School of Medicine (New Haven, Connecticut, USA). Briefly, HMC-1 cells were seeded in 96-well U-bottom plate at a density of 1 × 10^7^ cells/mL and stimulated with 100 ng/mL recombinant hTSLP alone or with **1** for 30 min. After stimulation, cells were fixed with BD Cytofix/Cytoperm (BD Biosciences, USA) solution for 10 min. Fixed cells were then permeabilized with ice-cold methanol for 30 min, and intracellular pSTAT5 was stained with Alexa Fluor 647 mouse anti-STAT5 (pY694; BD Biosciences, USA) (1:10 dilution in FACS buffer) for 30 min. Stained cells were further fixed with BD Cytofix/Cytoperm solution for 10 min and re-suspended in FACS buffer for flow cytometry analysis. Cells were analyzed by a BD LSR Fortessa Flow cytometry (BD Biosciences, USA). FACS data were analyzed using FlowJo software Ver 9. 7. 6 (Tree Star Inc., USA).

#### Western blot analysis

HMC-1 cells were pretreated in the absence or presence of compounds (0.1, 1, 3, 10 μM) and then stimulated with TSLP (20 ng/ml) for 2 h. Activated cells were lysed and separated by 10% sodium dodecyl sulfate-polyacrylamide gel electrophoresis (SDS-PAGE). After electrophoresis, proteins were transferred to nitrocellulose membranes. The membranes were blocked, incubated with primary antibodies (1:700 dilution in PBS with 0.05% Tween-20), and developed using peroxidase-conjugated secondary antibodies (1:3000 dilution in PBS with 0.05% Tween-20). Proteins were visualized by enhanced chemiluminescence (Amersham Bioseciences, Piscataway, NJ, USA) according to the manufacturer’s instructions.

### Animal experiments

#### Mice

DO11.10 mice were obtained from KAIST (Daejeon, Korea) and inbred with BALB/c mice. Female BALB/c mice (6 weeks old, 18–22 g) were obtained from Orientbio (Daejeon, Korea). Mice were housed in a facility maintained at a temperature of 20 to 22 °C with 12 h light/dark cycle and a relative humidity of 45 to 55%. Food and water were given *ad libitum*. All animal experiments were approved by the Institutional Animal Care and Use Committee of Korea University. We carried out all experiments in accordance with the approved guidelines. All efforts were made to minimize suffering during the experiments.

#### HDM-induced mouse model of allergic airway inflammation

Mice and treatment of compound **1**: DO11.10 mice were sacrificed and splenocytes were isolated. Approximately 1 × 10^6^ CD4 T cells from DO11.10 mice were transferred to naïve BALB/c mice intravenously. The following day, mice were challenged with a mixture of HDM (50 μg of *Dermatophagoides farinae* and *Dermatophagoides pteronyssinus* each; Greer Laboratories, Inc., USA) and 100 μg of OVA (Sigma-Aldrich Co., USA) for three days via the intranasal route. Mice were treated with **1** (200 μg) or PBS intraperitoneally three times every other day starting from day 2 post-challenge based on previous report^49^. Allergen-challenged mice were sacrificed on day 11 post-challenge.

Bronchoalveolar Lavage (BAL): Mice were sacrificed 3 days after the last treatment of compound **1**. Lungs were lavaged four times with 800 μL of 0.5% FBS each using tracheal capillary tube. BAL fluids were centrifuged at 1,500 rpm for 5 min and leukocytes were re-suspended with 1% RPMI. The total cell number was counted using a hemocytometer. Leukocytes were transferred to glass slides by Cytospin (Thermo Scientific, USA) at 2,800 rpm for 5 min, and stained with Diff-Quick staining (Sysmex Corporation, Japan).

Flow cytometry analysis: BAL cells were centrifuged at 1,650 rpm for 2 min in round bottom 96-well plate. Cells were stained with anti-CD4 FITC (RM4-5, Biolegend Inc., USA), anti-CD127 PE/Cy7 (A7R34, Biolegend Inc., USA), anti-CD62L APC/Cy7 (MEL14, Biolegend Inc., USA), anti-CD27 PE (LG.7F9, eBioscience Inc., USA), and anti-DO11.10APC (KJ1-26, eBioscience Inc., USA) antibodies for 30 min. Stained cells were examined by flow cytometry using LSR Fortessa ^TM^. Data were analyzed using FlowJo software Ver 9.7.6.

#### OVA-induced mouse model of allergic airway inflammation

Sensitization and airway challenge: For initial sensitization, BALB/c mice were administered intraperitoneally on day 0 with 20 μg of OVA emulsified in 200 μL of sterile PBS containing 1 mg of aluminum hydroxide adjuvant (Imject® Alum, Thermofisher, Korea). On day 14, the mice were boosted with the same allergen by IP injection. On day 21, mice were anesthetized with isoflurane (Hana Pharma, Seoul, Korea) and challenged intranasally with 40 μL of 1% ovalbumin in PBS, while the control group received PBS only. The mice were sacrificed at the indicated time points and BAL was performed.

Administration of compound **1**: Compound **1** was dissolved in dimethylacetamide: Tween 80: saline (10:10:80, v-v:v) to a concentration of 5 mg/mL, and administered intraperitoneally at either 10, 50, or 100 mg/kg, 1 h prior to intranasal OVA challenge. The control group received vehicle only.

BAL: Immediately after sacrifice, lungs were lavaged as described above. Total leukocyte numbers were counted. Differential cell counts were performed by counting at least 200 cells on cytocentrifuged samples stained with Diff-Quick solution.

### Statistical analysis

Statistical analysis was performed using GraphPad Prism 5 (GraphPad Software, USA). Two-tailed analysis of variance (ANOVA) and Student’s *t*-test were performed to calculate statistical significance which was set at * for *P* < 0.05.

## Supplementary information

Supporting information for Structure-Activity Relationships of Baicalein and Its Analogs as Novel TSLP Inhibitors
