# Author Correction: Structure-Activity Relationships of Baicalein and its Analogs as Novel TSLP Inhibitors

**DOI:** 10.1038/s41598-020-58615-1

**Published:** 2020-01-28

**Authors:** Bernie Byunghoon Park, Jae Wan Choi, Dawon Park, Doyoung Choi, Jiwon Paek, Hyun Jung Kim, Se-Young Son, Ameeq Ul Mushtaq, Hyeji Shin, Sang Hoon Kim, Yuanyuan Zhou, Taehyeong Lim, Ji Young Park, Ji-Young Baek, Kyul Kim, Hongmok Kwon, Sang-Hyun Son, Ka Young Chung, Hyun-Ja Jeong, Hyung-Min Kim, Yong Woo Jung, Kiho Lee, Ki Yong Lee, Youngjoo Byun, Young Ho Jeon

**Affiliations:** 1 College of Pharmacy, Korea University, 2511 Sejong-ro, Sejong, 30019 Republic of Korea; 2 Korea Brain Research Institute (KBRI), 61 Cheomdan-ro, Dong-gu, Daegu 41062 Republic of Korea; 3 School of Pharmacy, Sungkyunkwan University, 2066 Seoburo, Jangan-gu, Suwon 16419 Republic of Korea; 4 Department of Food Science & Technology, Hoseo University, 20 Hoseo-ro 79 beon-gil, Baebang-eup, Asan, Chungcheongnam-do 31499 Republic of Korea; 5 Department of Pharmacology, College of Korean Medicine, Kyung Hee University, 26 Kyungheedae-ro, Dongdaemun-gu, Seoul 02447 Republic of Korea; 6 Biomedical Research Center, Korea University Guro Hospital, 148 Gurodong-ro, Guro-gu, Seoul 08308 Republic of Korea

Correction to: *Scientific Reports* 10.1038/s41598-019-44853-5, published online 19 June 2019

The authors of this Article neglected to cite a paper which reported the crystal structure of hTLSP, which should be cited as below:

In the Results and Discussion section, under subheading ‘Binding of compound 1 to hTLSP’,

“Docking studies of **1** with hTSLP suggested that **1** bound to the hTSLPR-binding interface of hTSLP.”

should read:

“Docking studies of **1** with hTSLP suggested that **1** bound to the hTSLPR-binding interface of hTSLP^1^.”

In the Experimental Procedures section, under subheading ‘Docking simulations’,

“Human TSLP structure (PDB ID: 5J11) was prepared using Gasteiger charge, protein structure was kept rigid in docking, and binding site of compound **1** was defined from CSP-mapped residues obtained from NMR binding experiments.”

should read:

“Human TSLP structure (PDB ID: 5J11) was prepared using Gasteiger charge, protein structure was kept rigid in docking, and binding site of compound **1** was defined from CSP-mapped residues obtained from NMR binding experiments^1^.”
