# Supplementary material for: Structure-Activity Relationships of Baicalein and its Analogs as Novel TSLP Inhibitors
Source: Sci Rep. 2019 Jun 19;9:8762. doi: 10.1038/s41598-019-44853-5 (PMC6584507; doi:10.1038/s41598-019-44853-5)
Supplement: Supplementary file 1 — Supporting information for Structure-Activity Relationships of Baicalein and Its Analogs as Novel TSLP Inhibitors [file 41598_2019_44853_MOESM1_ESM.docx]

***Supporting Information for***

**Structure-Activity Relationships of Baicalein and Its Analogs as Novel TSLP Inhibitors**

Bernie Byunghoon Park^1,#^, Jae Wan Choi^1,#^, Dawon Park^1,#^, Doyoung Choi^1^,Jiwon Paek^1^, Hyun Jung Kim^2^, Se-Young Son^1^, Ameeq Ul Mushtaq^1^, Hyeji Shin^1^, Sang Hoon Kim^1^, Yuanyuan Zhou^1^, Taehyeong Lim^1^, Ji Young Park^3^, Ji-Young Baek^1^, Kyul Kim^1^, Hongmok Kwon^1^, Sang-Hyun Son^1^, Ka Young Chung^3^,Hyun-Ja Jeong^4^, Hyung-Min Kim^5^, Yong Woo Jung^1^, Kiho Lee^1^, Ki Yong Lee^1,*^,Youngjoo Byun^1,6,*^,Young Ho Jeon^1,*^

^1^College of Pharmacy, Korea University, 2511 Sejong-ro, Sejong 30019, Republic of Korea

^2^Korea Brain Research Institute (KBRI), 61 Cheomdan-ro, Dong-gu, Daegu 41062, Republic of Korea

^3^School of Pharmacy, Sungkyunkwan University, 2066 Seoburo, Jangan-gu, Suwon 16419, Republic of Korea

^4^Department of Food Science & Technology, Hoseo University, 20Hoseo-ro 79beon-gil, Baebang-eup, Asan, Chungcheongnam-do 31499, Republic of Korea

^5^Department of Pharmacology, College of Korean Medicine, Kyung Hee University, 26 Kyungheedae-ro, Dongdaemun-gu, Seoul, 02447, Republic of Korea

^6^Biomedical Research Center, Korea University Guro Hospital, 148 Gurodong-ro, Guro-gu, Seoul 08308, Republic of Korea

**^#^** Equal contribution

^*^ Corresponding authors:

Ki Yong Lee ([kylee11@korea.ac.kr](mailto:kylee11@korea.ac.kr)), College of Pharmacy, Korea University, Sejong 30019, Korea

Youngjoo Byun ([yjbyun1@korea.ac.kr](mailto:yjbyun1@korea.ac.kr)), College of Pharmacy, Korea University, Sejong 30019, Korea

Young Ho Jeon ([yhjeon@korea.ac.kr](mailto:yhjeon@korea.ac.kr)), College of Pharmacy, Korea University, Sejong 30019, Korea

**Table of contents**

**Content** **page**

^1^H NMR and HRMS spectra of compound **4a**  S1 and S2

^1^H NMR and HRMS spectra of compound **4b**  S3 and S4

^1^H NMR and HRMS spectra of compound **4c**  S5 and S6

^1^H NMR and HRMS spectra of compound **4d**  S7 and S8

^1^H NMR andHRMS spectra of compound **5a** S9 and S10

^1^H NMR and HRMS spectra of compound **5b**  S11 and S12

^1^H NMR and HRMS spectra of compound **5c**  S13 and S14

^1^H NMR and HRMS spectra of compound **5d**  S15 and S16

^1^H NMR and HRMS spectra of compound **6a**  S17 and S18

^1^H NMR and HRMS spectra of compound **6b**  S19 and S20

^1^H NMR and HRMS spectra of compound **6c**  S21 and S22

^1^H NMR and HRMS spectra of compound **6d**  S23 and S24

^1^H NMR and HRMS spectra of compound **7a**  S25 and S26

^1^H NMR and HRMS spectra of compound **7b**  S27 and S28

^1^H NMR and HRMS spectra of compound **8a**  S29 and S30

^1^H NMR, ^13^C NMR, and HRMS spectra of compound **8b**  S31, S32 and S33

^1^H NMR, ^13^C NMR, and HRMS spectra of compound **8c**  S34, S35, and S36

**Table of contents**

**Content** **page**

^1^H NMR, ^13^C NMR, and HRMS spectra of compound **9a**  S37, S38, and S39

^1^H NMR spectra of compound **9b**  S40

^1^H NMR, ^13^C NMR, and HRMS spectra of compound **9c**  S41, S42, and S43

^1^H NMR, ^13^C NMR, and HRMS spectra of compound **9d**  S44, S45, and S46

^1^H NMR, ^13^C NMR, and HRMS spectra of compound **9e**  S47, S48, and S49

^1^H NMR, ^13^C NMR, and HRMS spectra of compound **9f**  S50, S51, and S52

^1^H NMR, ^13^C NMR, and HRMS spectra of compound **9g**  S53, S54, and S55

^1^H NMR spectra of compound **9h**  S56

^1^H NMR and HRMS spectra of compound **9i**  S57 and S58

^1^H NMR, ^13^C NMR, and HRMS spectra of compound **10a**  S59, S60, and S61

^1^H NMR ^13^C NMR, and HRMS spectra of compound **10b**  S62, S63 and S64

^1^H NMR, ^13^C NMR, and HRMS spectra of compound **10c**  S65, S66, and S67

^1^H NMR, ^13^C NMR, and HRMS spectra of compound **10d**  S68, S69, and S70

^1^H NMR, ^13^C NMR, and HRMS spectra of compound **10e**  S71, S72, and S73

^1^H NMR,^13^C NMR, and HRMS spectra of compound **10f**  S74, S75,and S76

^1^H NMR and HRMS spectra of compound **10g**  S77and S78

^1^H NMR and HRMS spectra of compound **10h**  S79 and S80

**Table of contents**

**Content** **page**

^1^H NMR and HRMS spectra of compound **10i**  S81 and S82

^1^H NMR, ^13^C NMR, and HRMS spectra of compound **11a**  S83, S84, and S85

**Figure S1**. Carbocation resonance structure of compound **11a** S86

**Figure S2.** Western blot (TSLP, upper) and SDS-PAGE (TSLPR, lower) results
from the pull-down assay S88

**Figure S3.** Plot of the bound fraction of compound **1**
in the titration of the TSLP solution S88

**Figure S4.** Western blot of compounds **10e-10g** compared with compound **1** S89

**Table 1.** STAT5 phosphorylation % of in-house natural products S90 and S91

**Table 2.** TSLP-inhibitory activities of in-house natural products by ELISA S92 and S93

^1^H NMR spectra of compound **4a** measuredin CDCl_3_ at 300 MHz


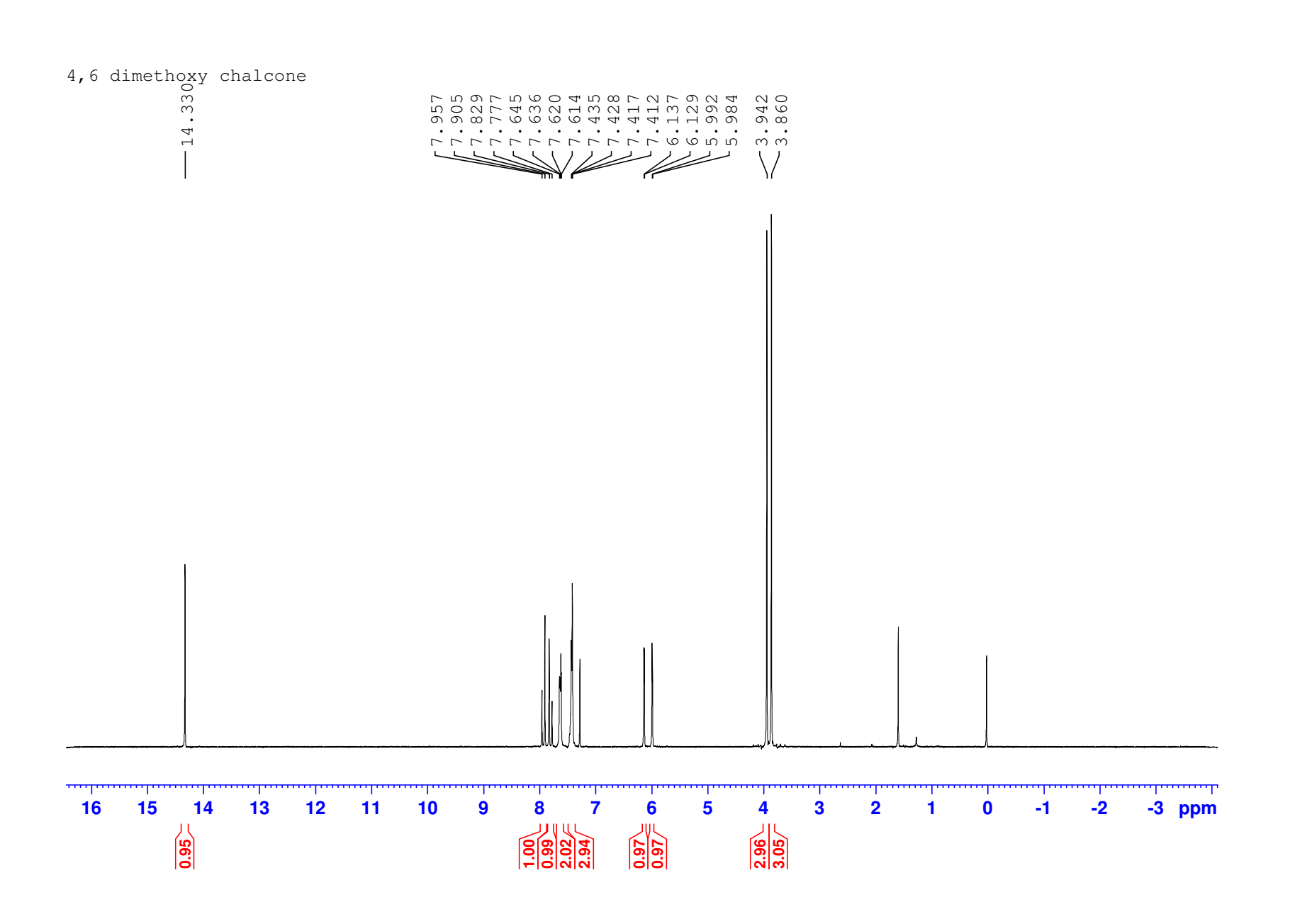


HRMS spectra of compound**4a**

^1^H NMR spectra of compound **4b** measured in CDCl_3_ at 300 MHz

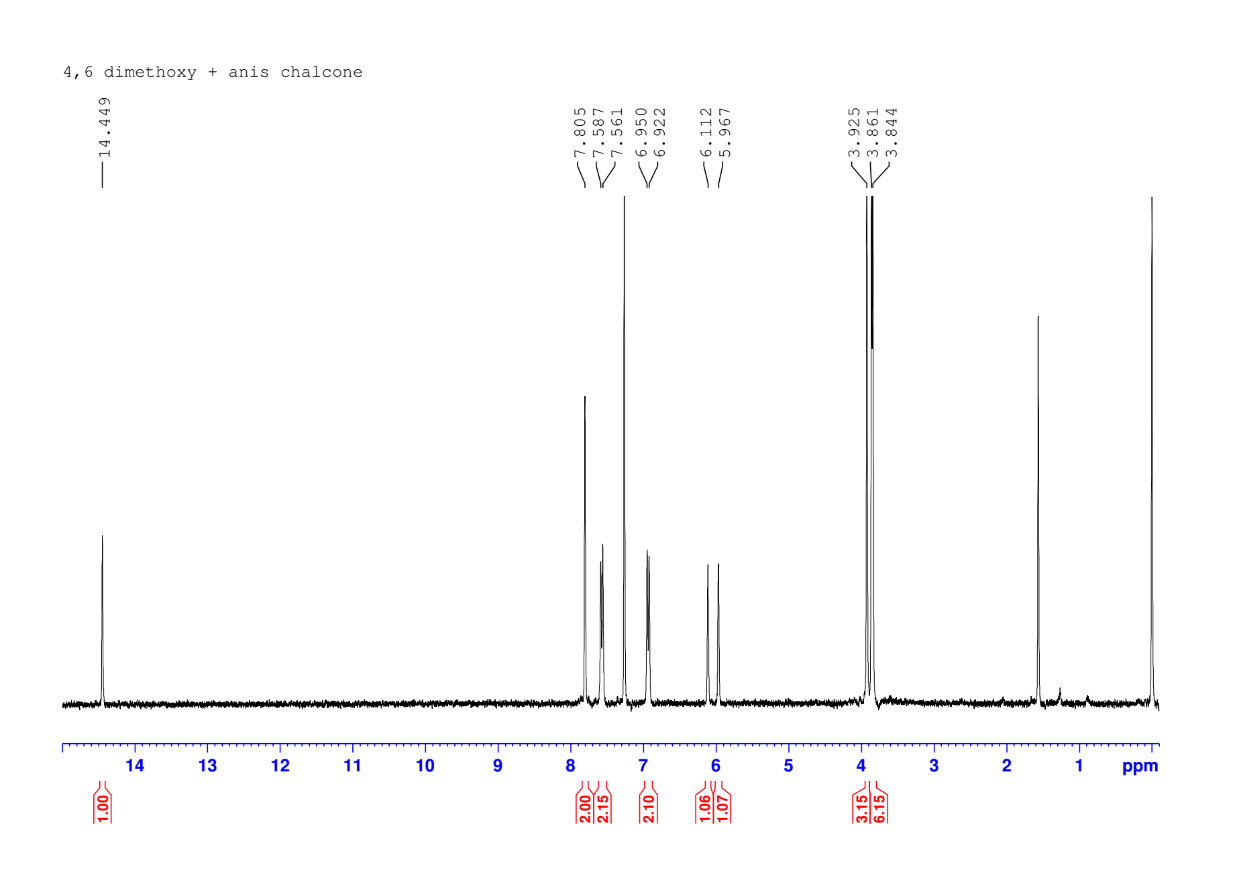


HRMS spectra of compound **4b**

^1^H NMR spectra of compound **4c** measured in CDCl_3_ at 300 MHz

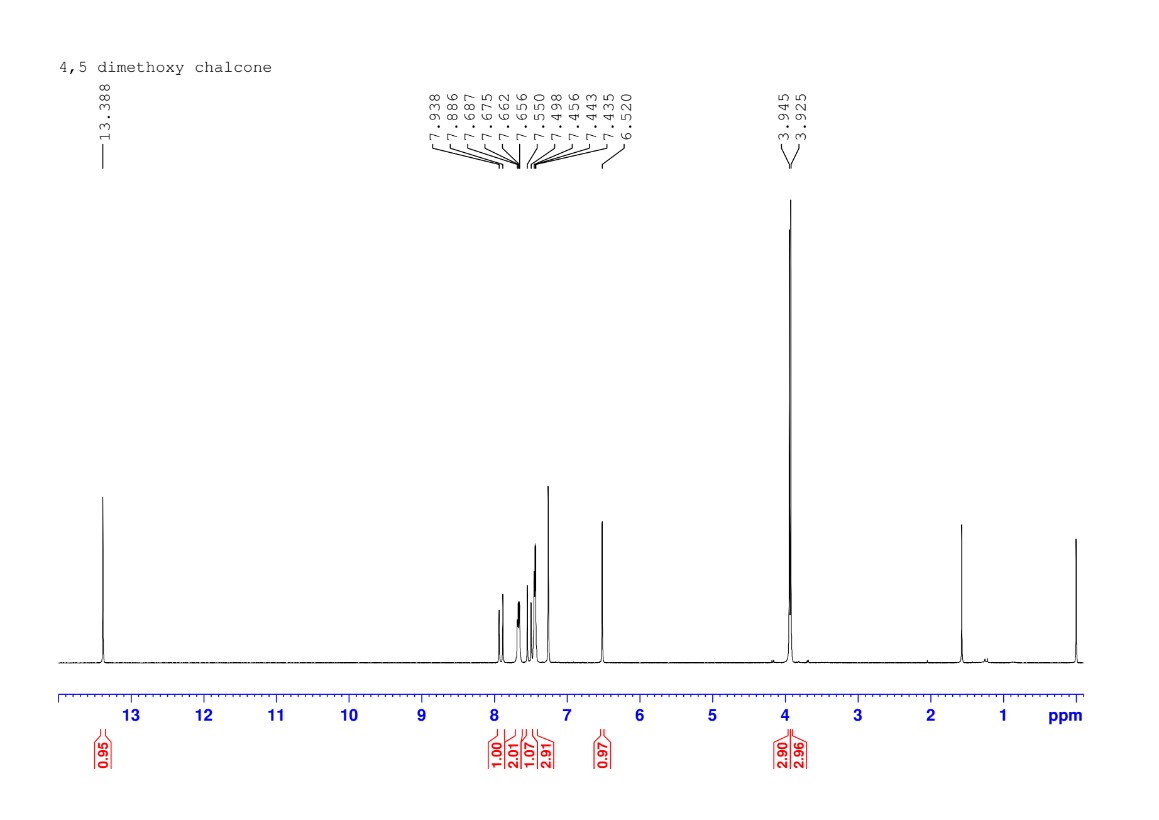


HRMS spectra of compound **4c**

^1^H NMR spectra of compound **4d** measured in CDCl_3_ at 300 MHz

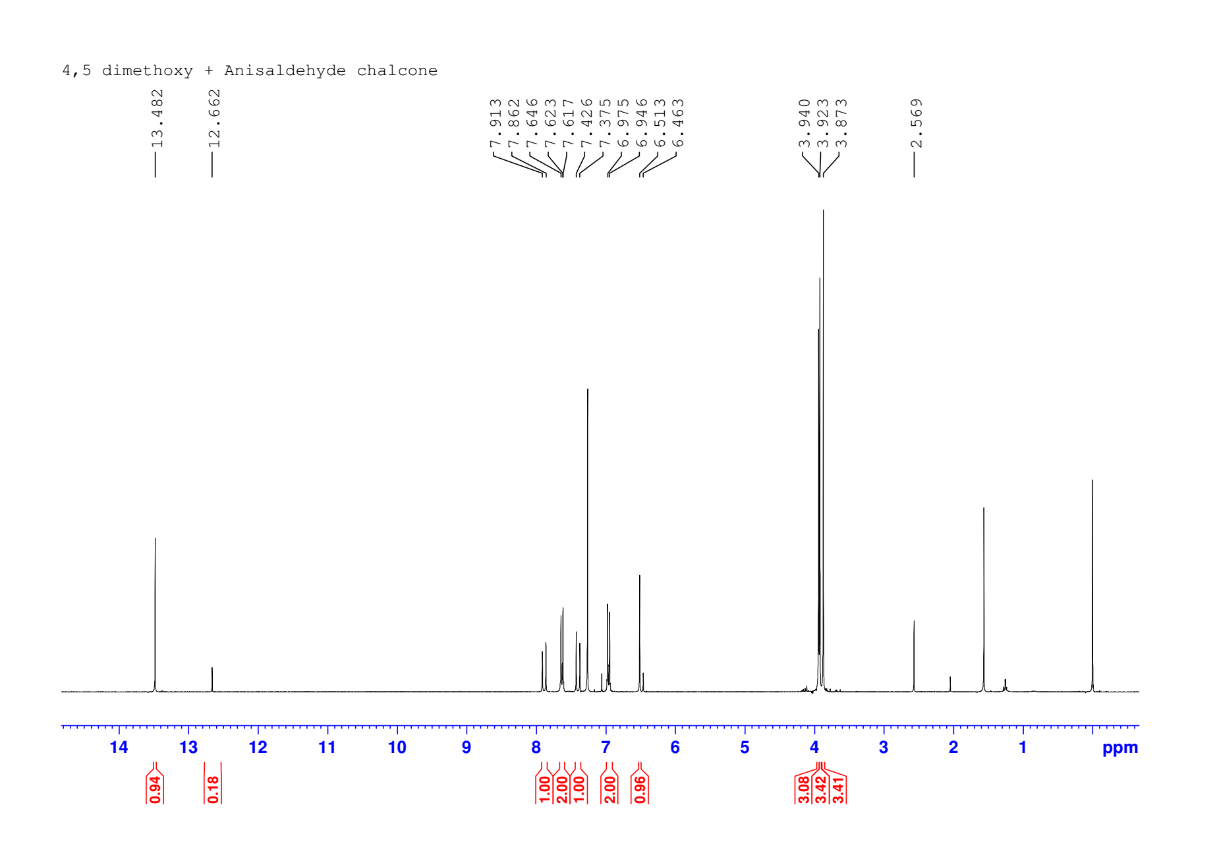


HRMS spectra of compound **4d**

^1^H NMR spectra of compound **5a** measured in CDCl_3_ at 300 MHz

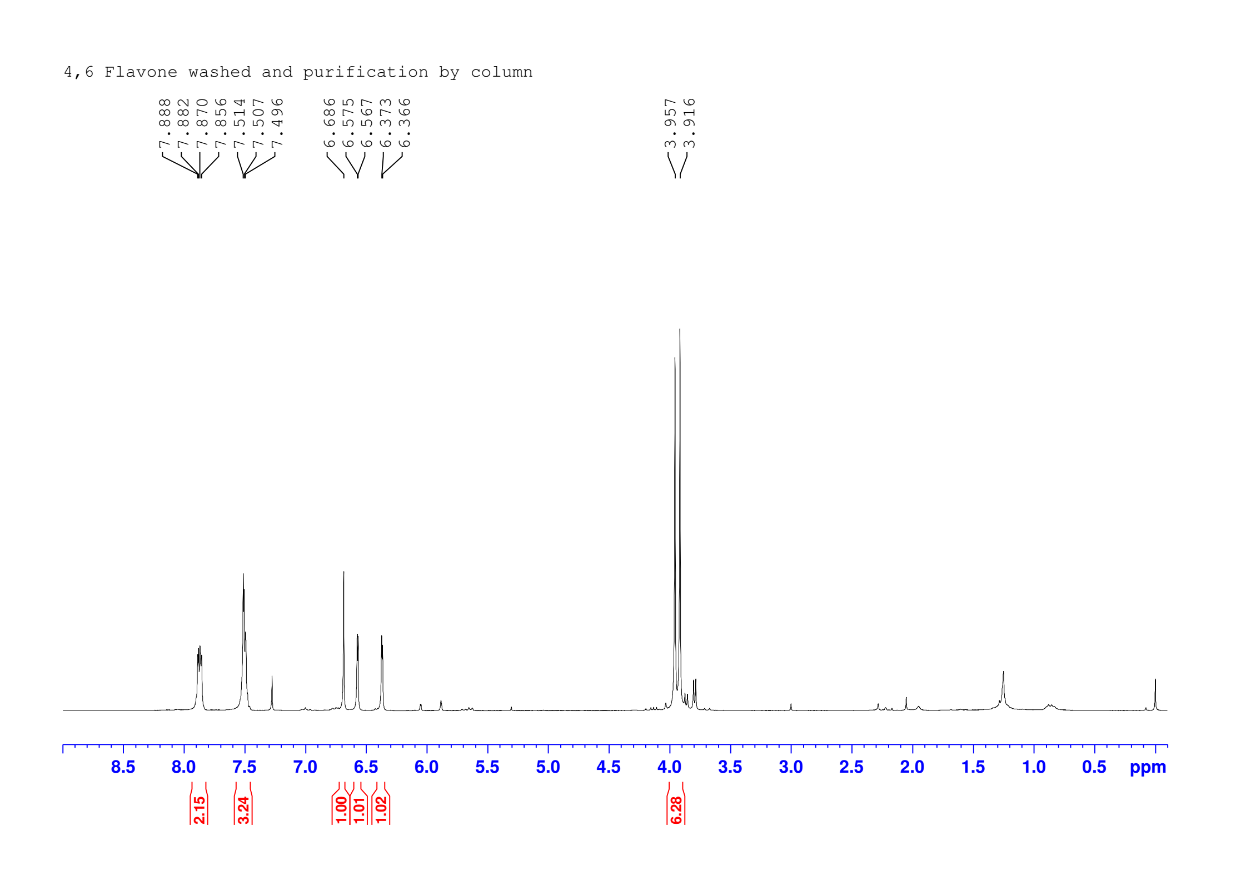


HRMS spectra of compound **5a**

^1^H NMR spectra of compound **5b** measured in CDCl_3_ at 300 MHz

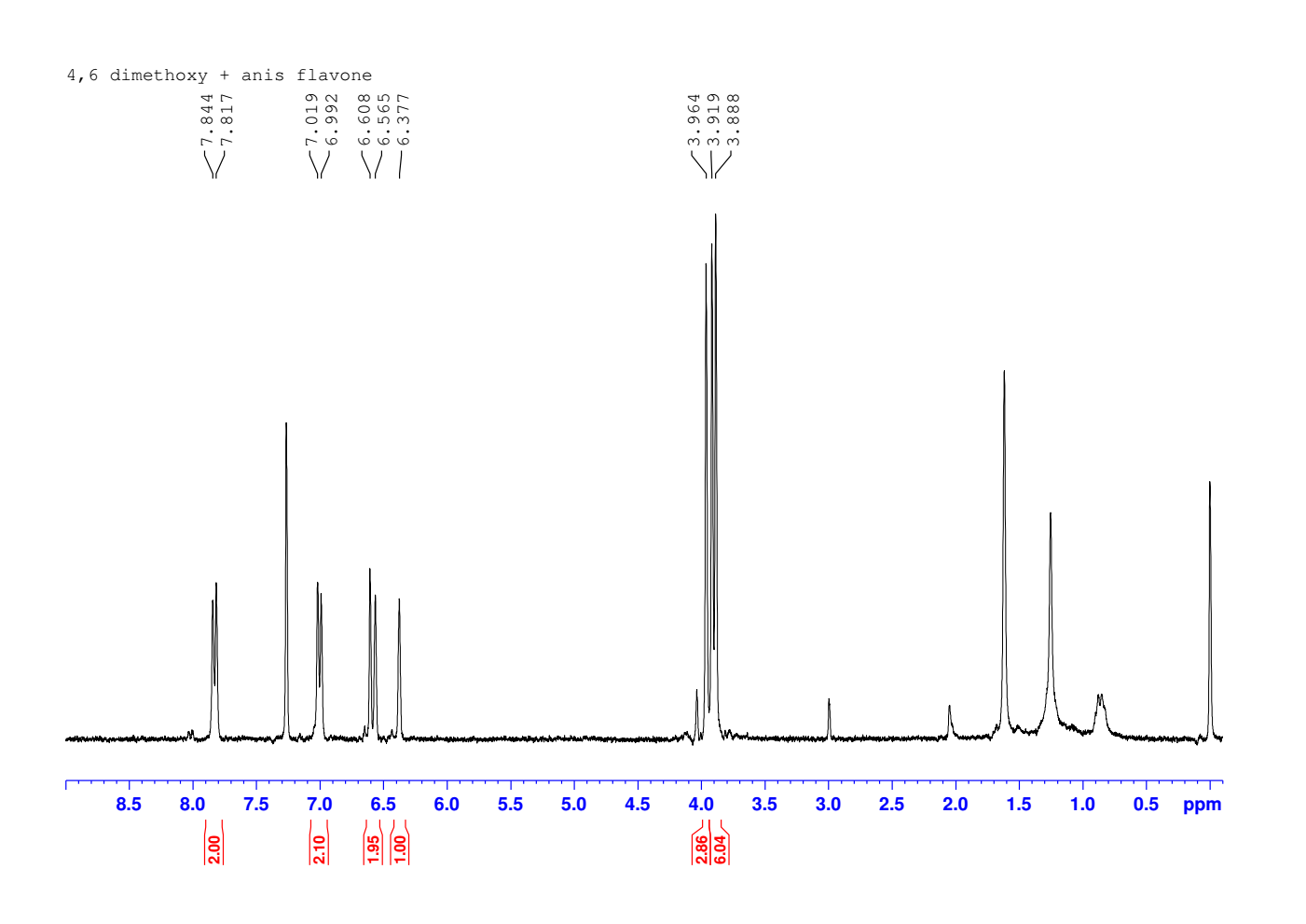


HRMS spectra of compound **5b**

^1^H NMR spectra of compound **5c** measured in CDCl_3_ at 300 MHz

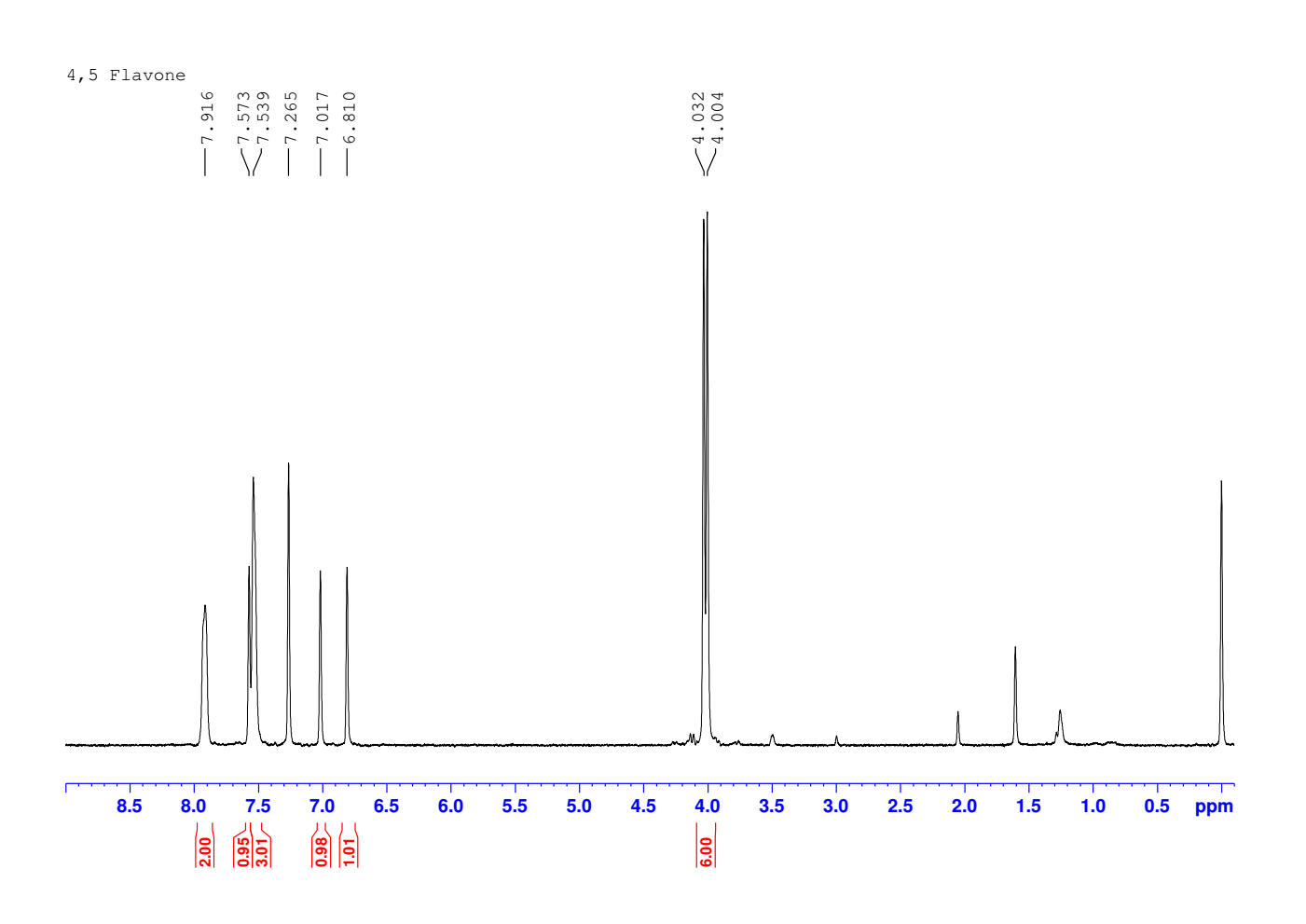


HRMS spectra of compound **5c**

^1^H NMR spectra of compound **5d** measured in CDCl_3_ at 300 MHz

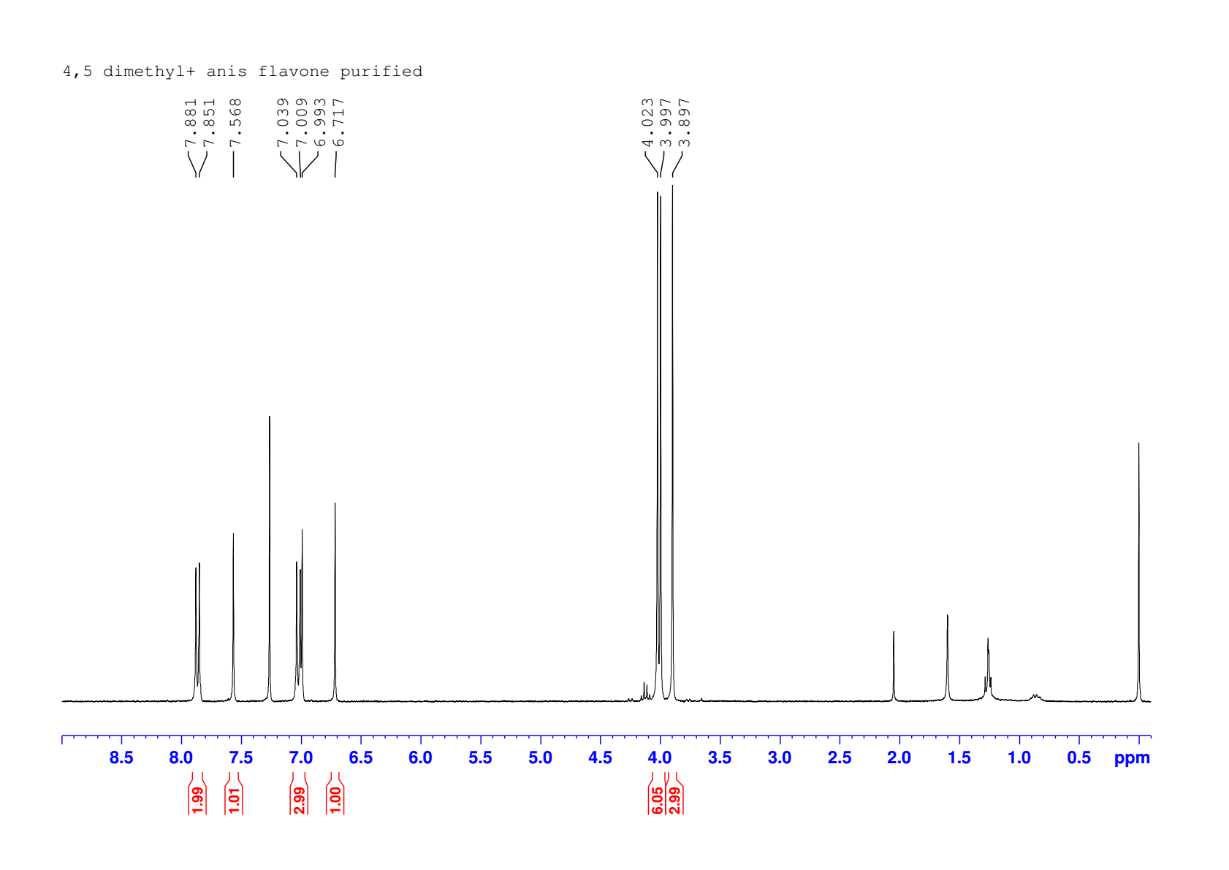


HRMS spectra of compound **5d**

^1^H NMR spectra of compound **6a** measured in CDCl_3_ at 300 MHz

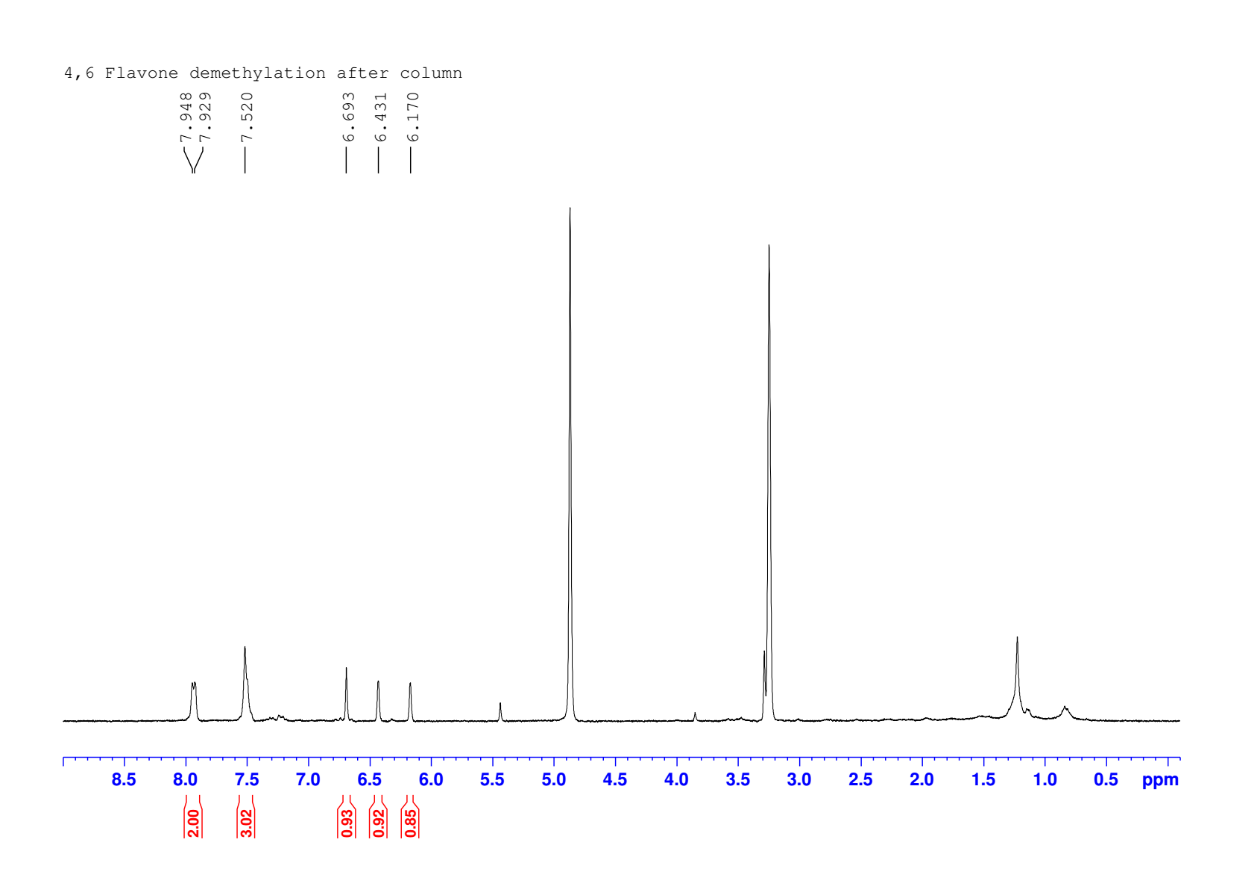


HRMS spectra of compound **6a**

^1^H NMR spectra of compound **6b** measured in CDCl_3_ at 300 MHz

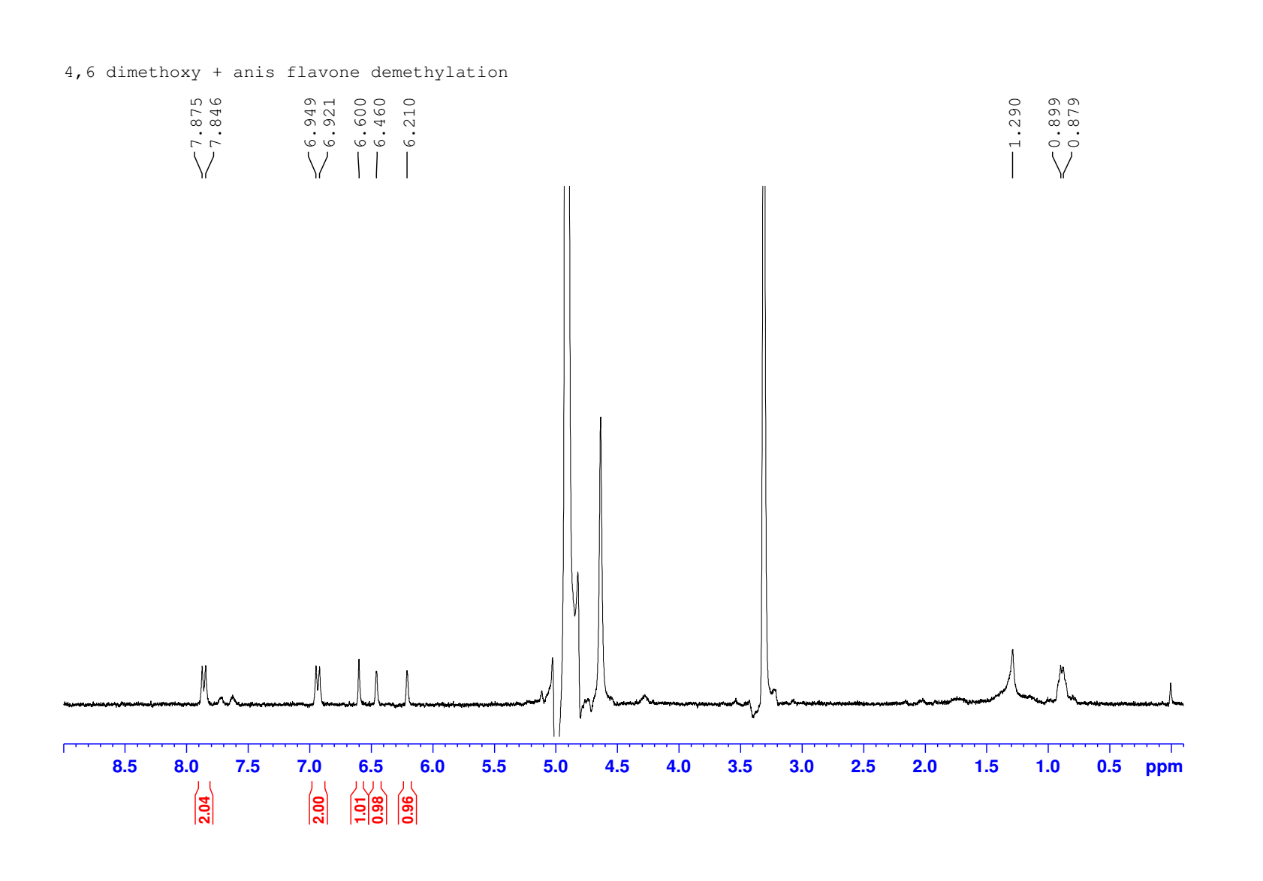


HRMS spectra of compound **6b**

^1^H NMR spectra of compound **6c** measured in CD_3_OD at 300 MHz

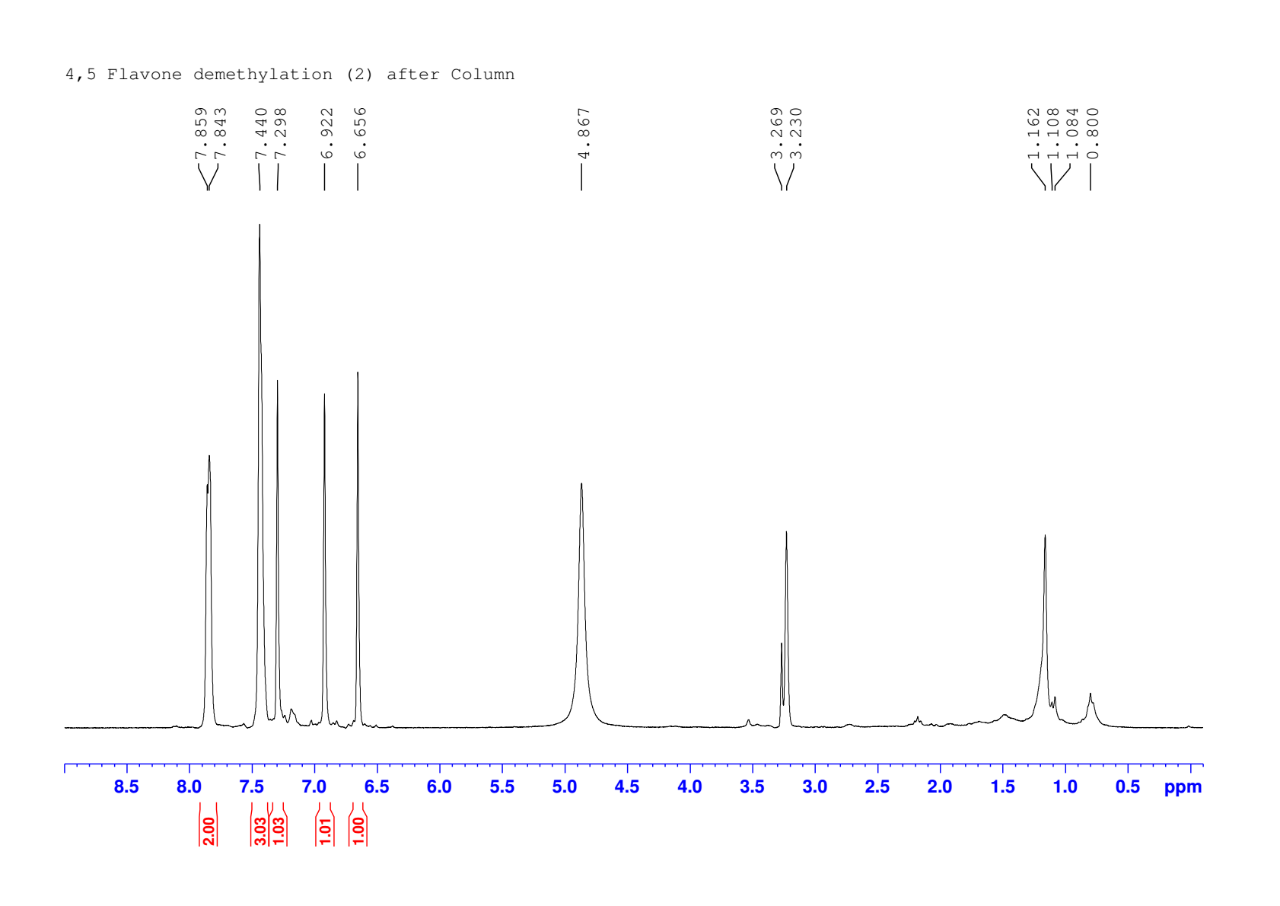


HRMS spectra of compound **6c**

^1^H NMR spectra of compound **6d** measured in CD_3_OD at 300 MHz

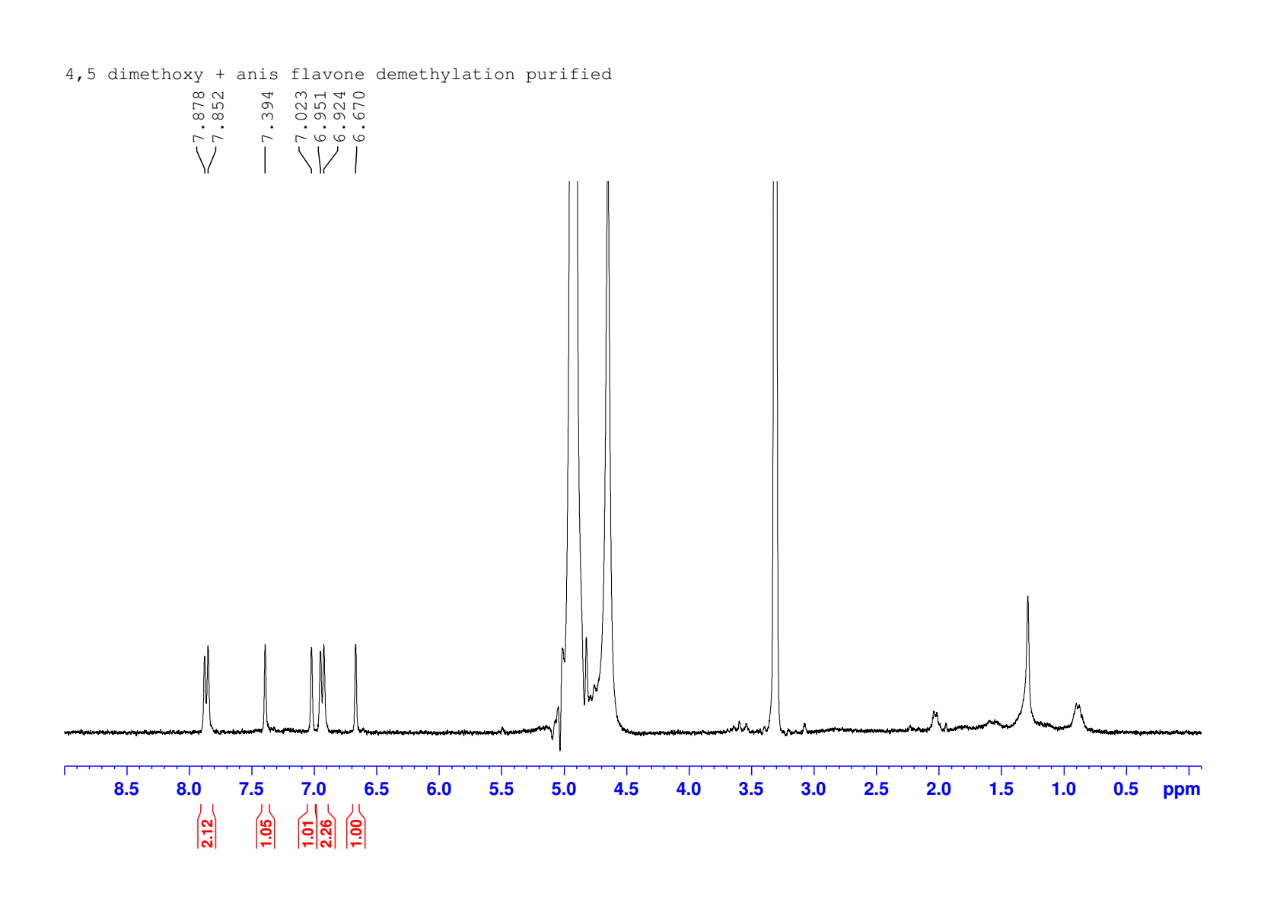


HRMS spectra of compound **6d**

^1^H NMR spectra of compound **7a** measured in (CD_3_)_2_CO at 300 MHz

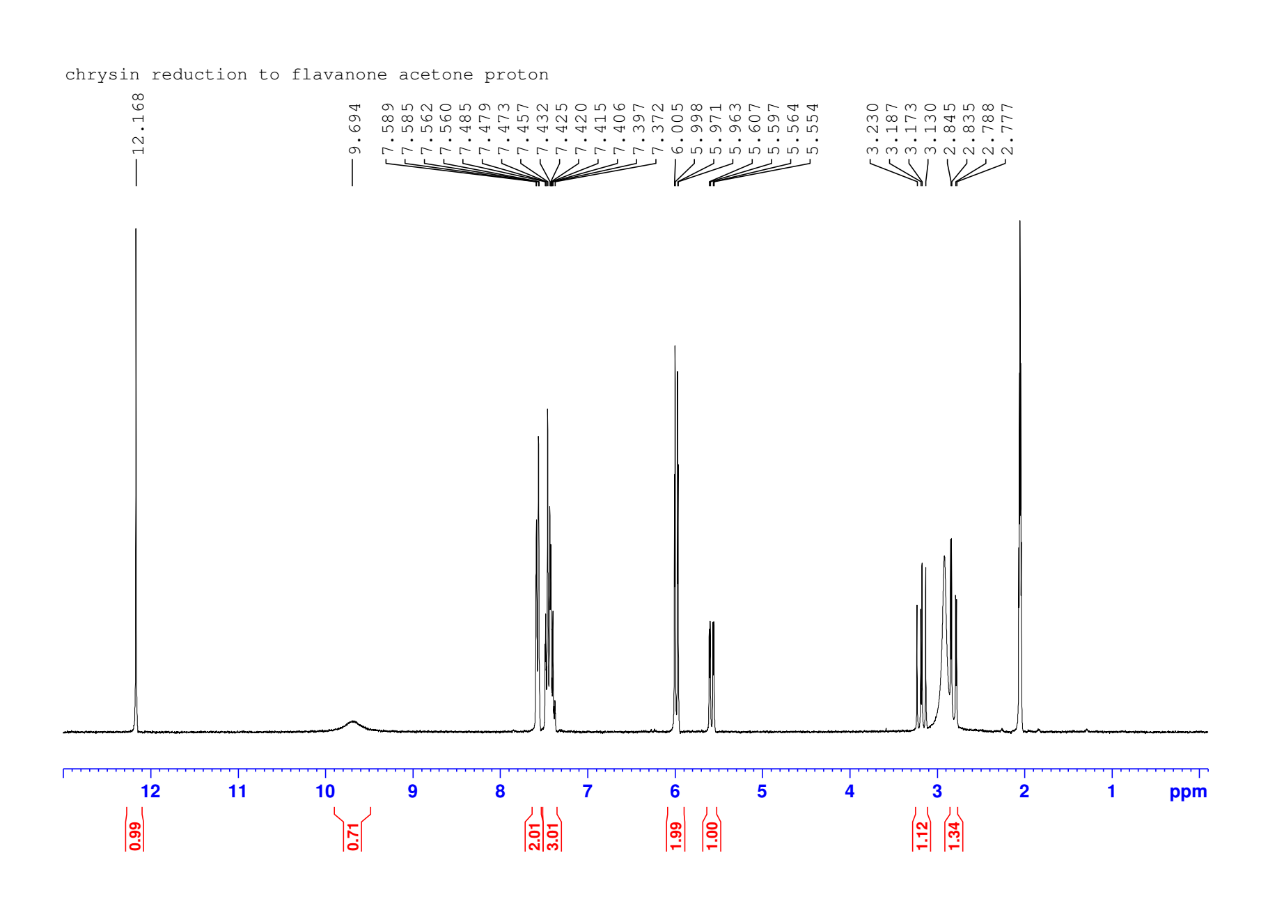


HRMS spectra of compound **7a**

^1^H NMR spectra of compound **7b** measured in (CD_3_)_2_CO at 300 MHz

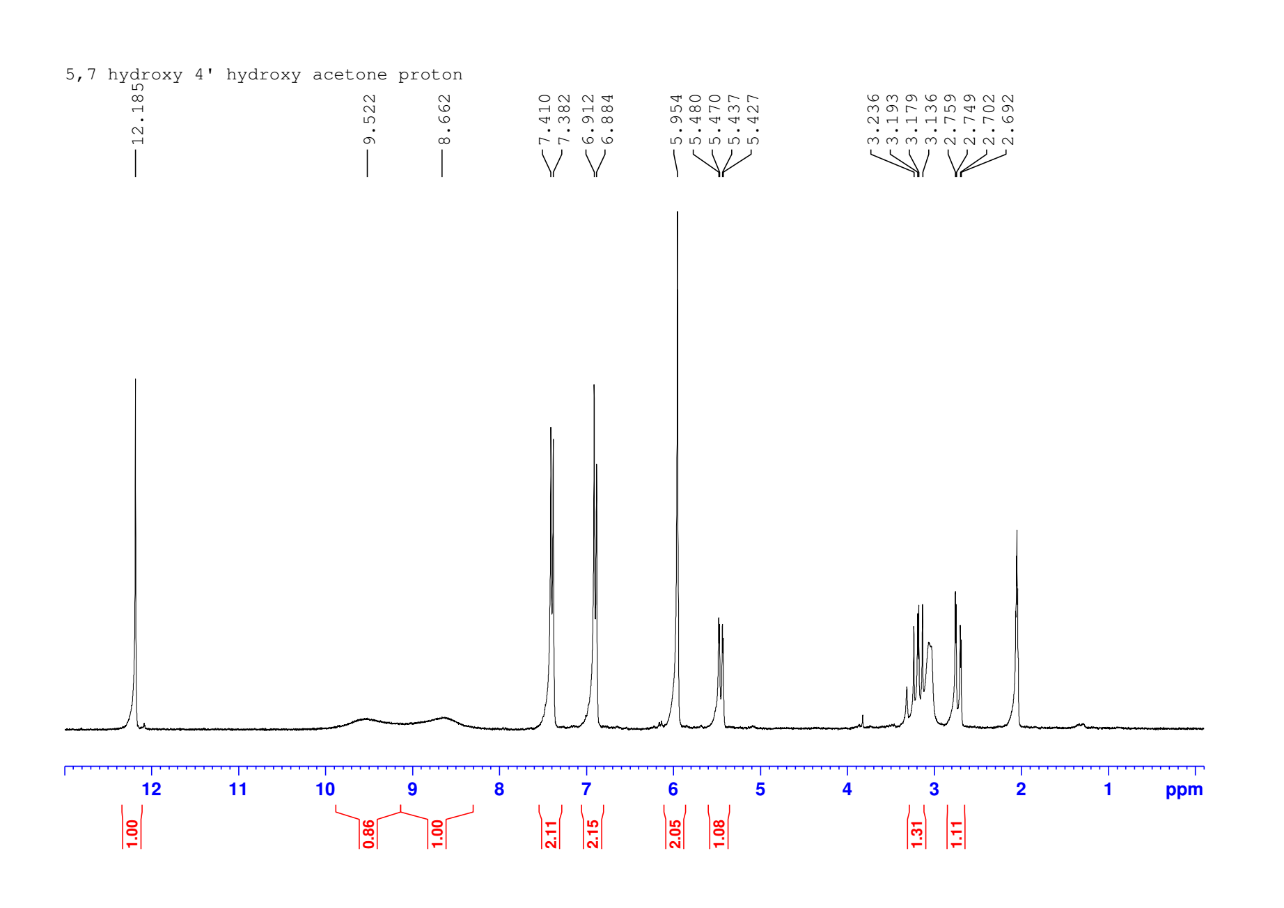


HRMS spectra of compound **7b**

^1^H NMR spectra of compound **8a** measured in CDCl_3_ at 300 MHz

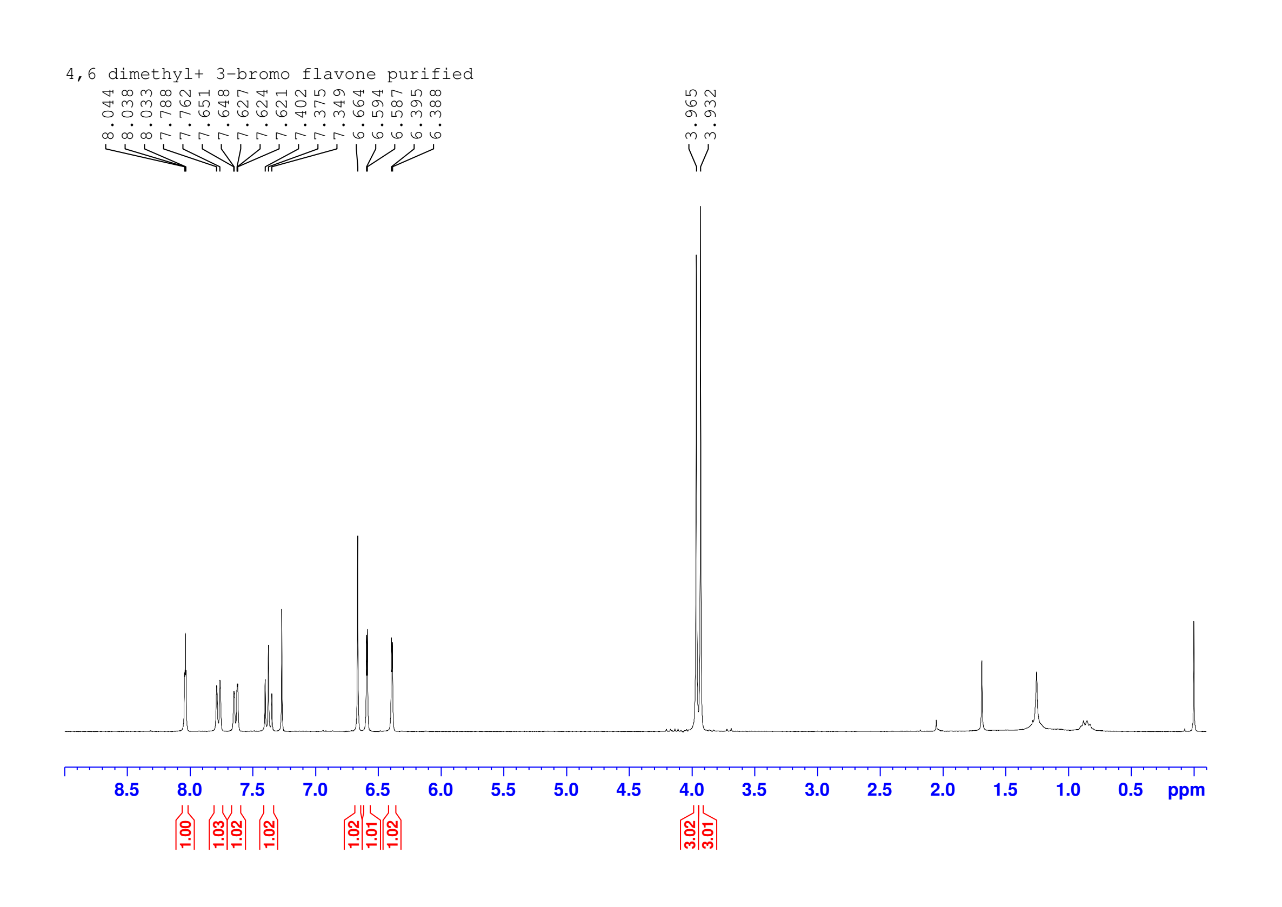


HRMS spectra of compound **8a**

^1^H NMR spectra of compound **8b** measured in CDCl_3_ at 300 MHz

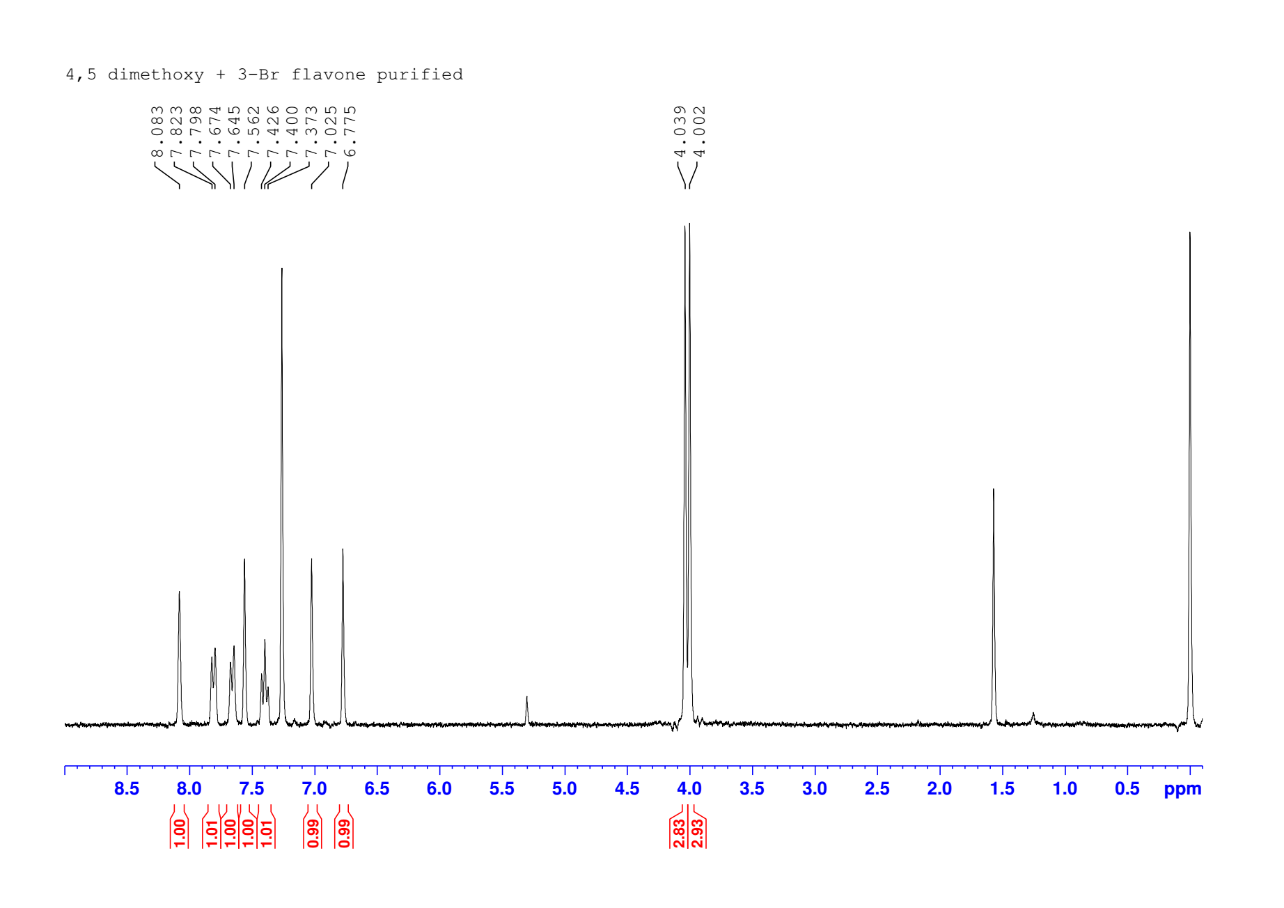


^13^C NMR spectra of compound **8b** measured in CDCl_3_ at 75 MHz

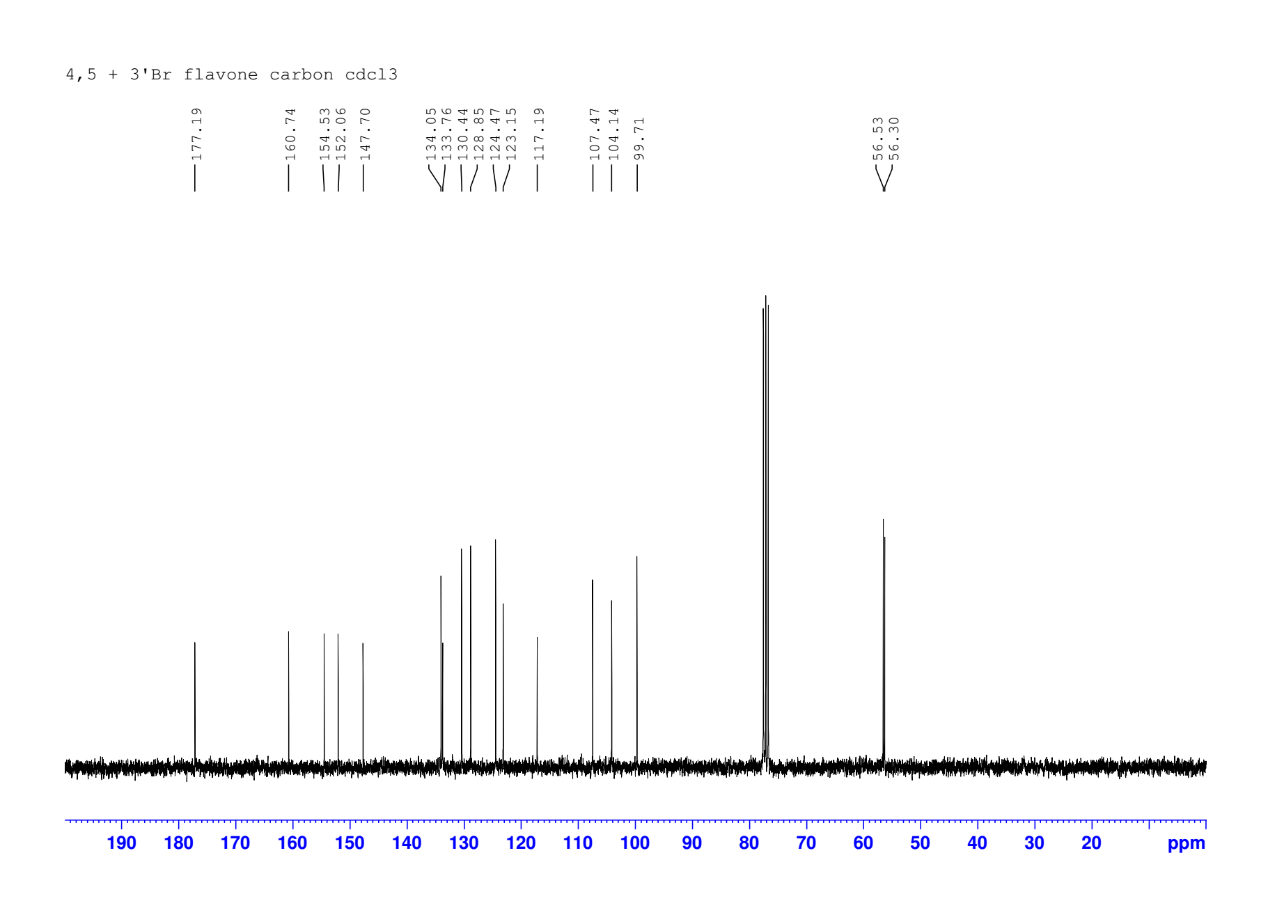


HRMS spectra of compound **8b**

^1^H NMR spectra of compound **8c** measured in CDCl_3_ at 300 MHz

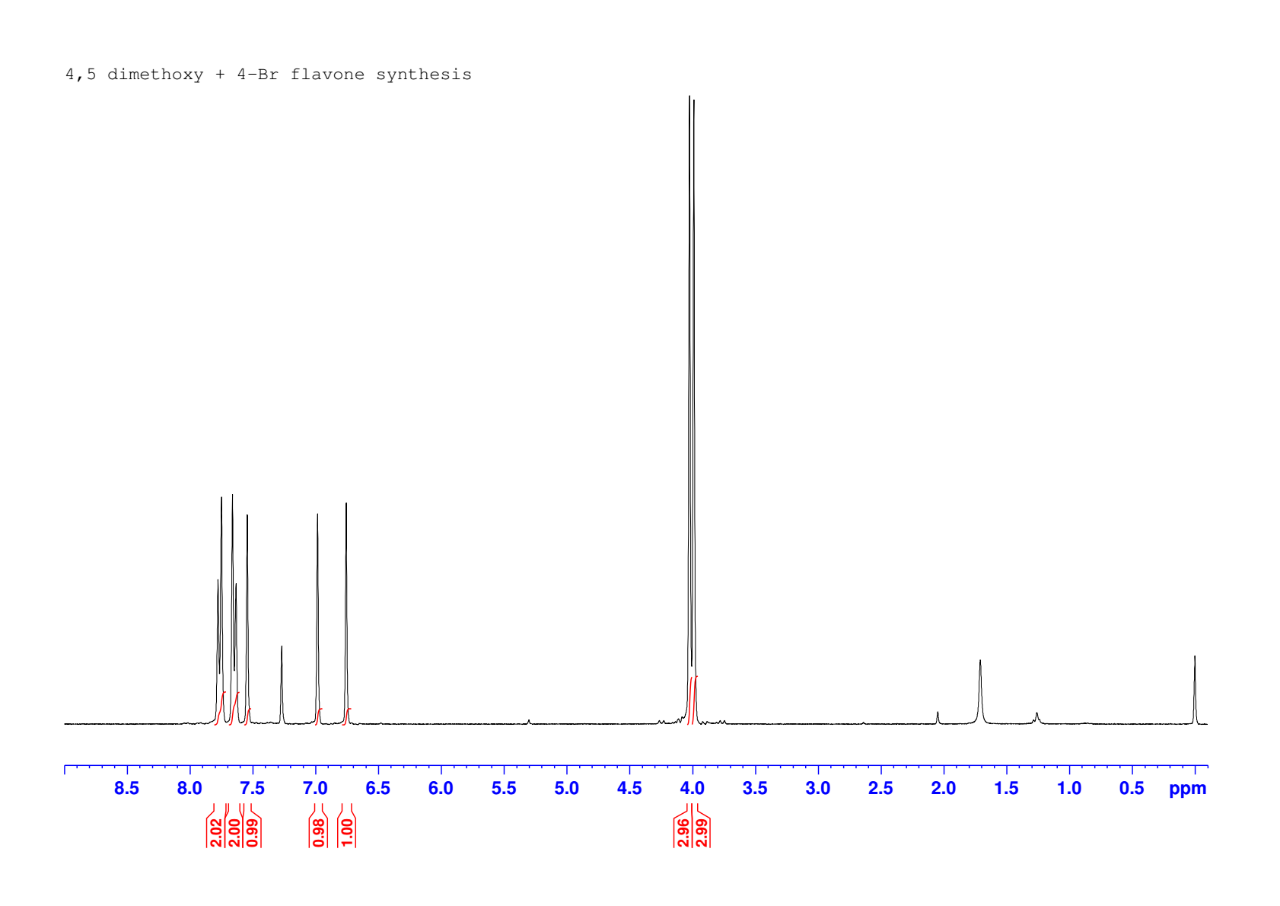


^13^C NMR spectra of compound **8c** measured in CDCl_3_ at 75 MHz

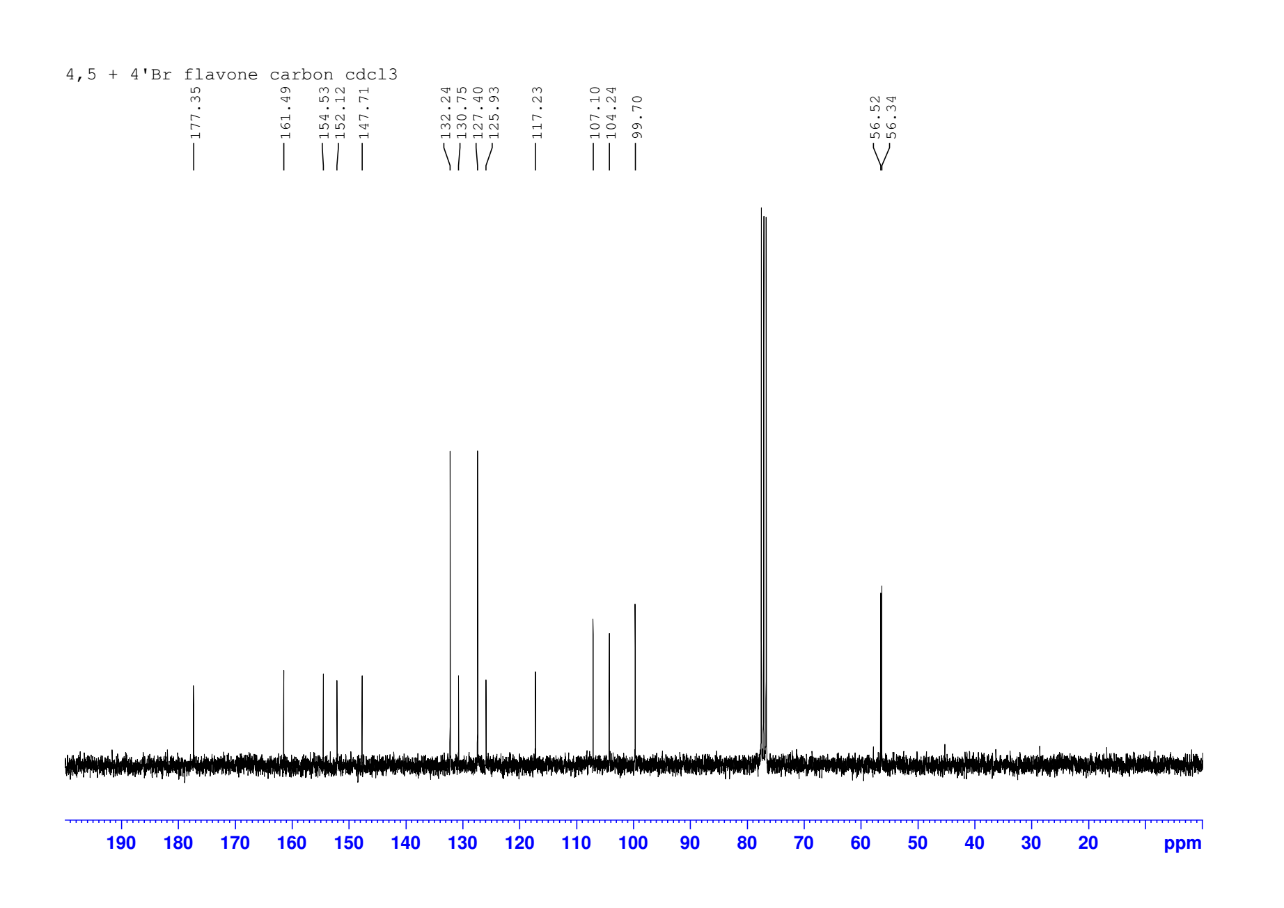


HRMS spectra of compound **8c**

^1^H NMR spectra of compound **9a** measured in CDCl_3_ at 300 MHz

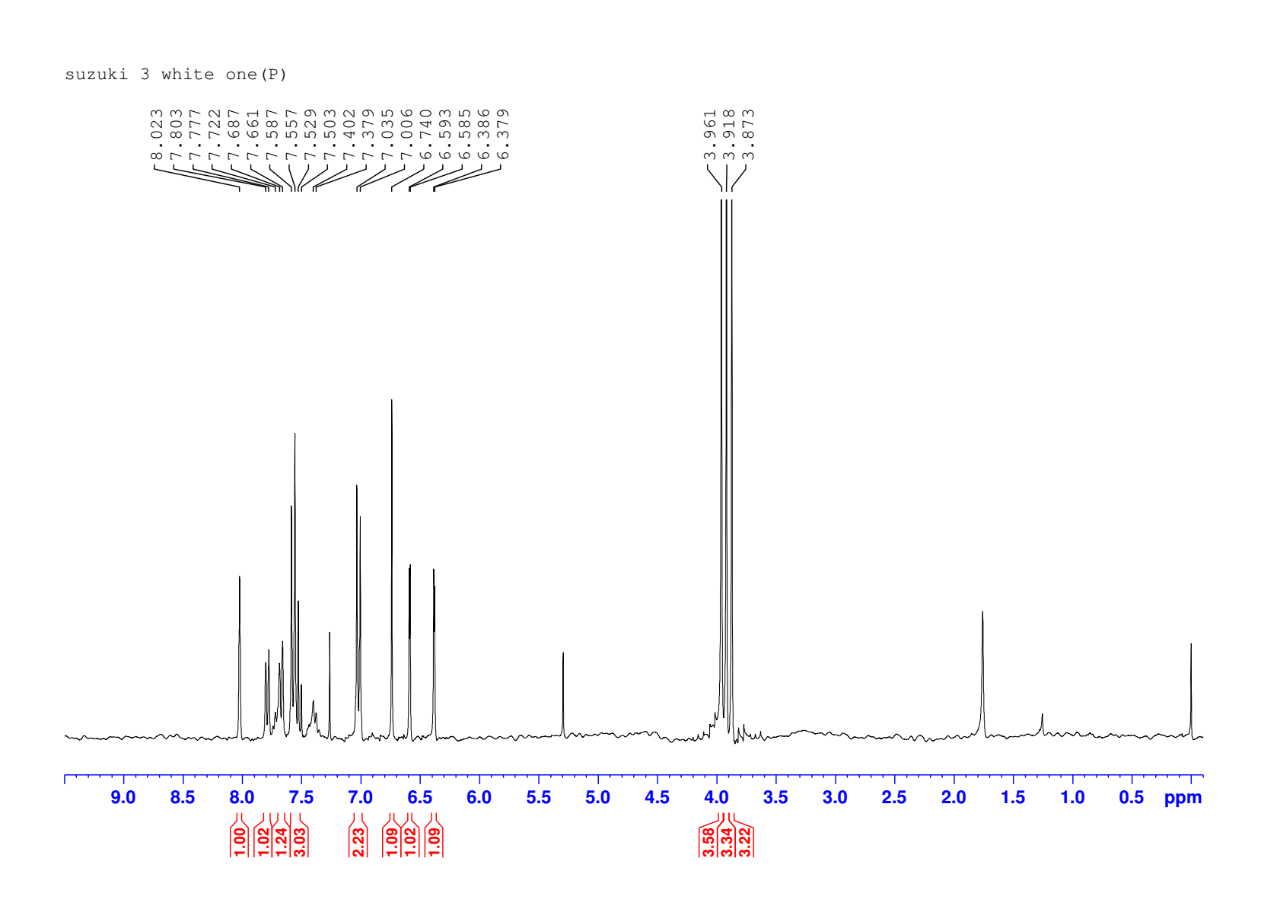


^13^C NMR spectra of compound **9a** measured in CDCl_3_ at 75 MHz

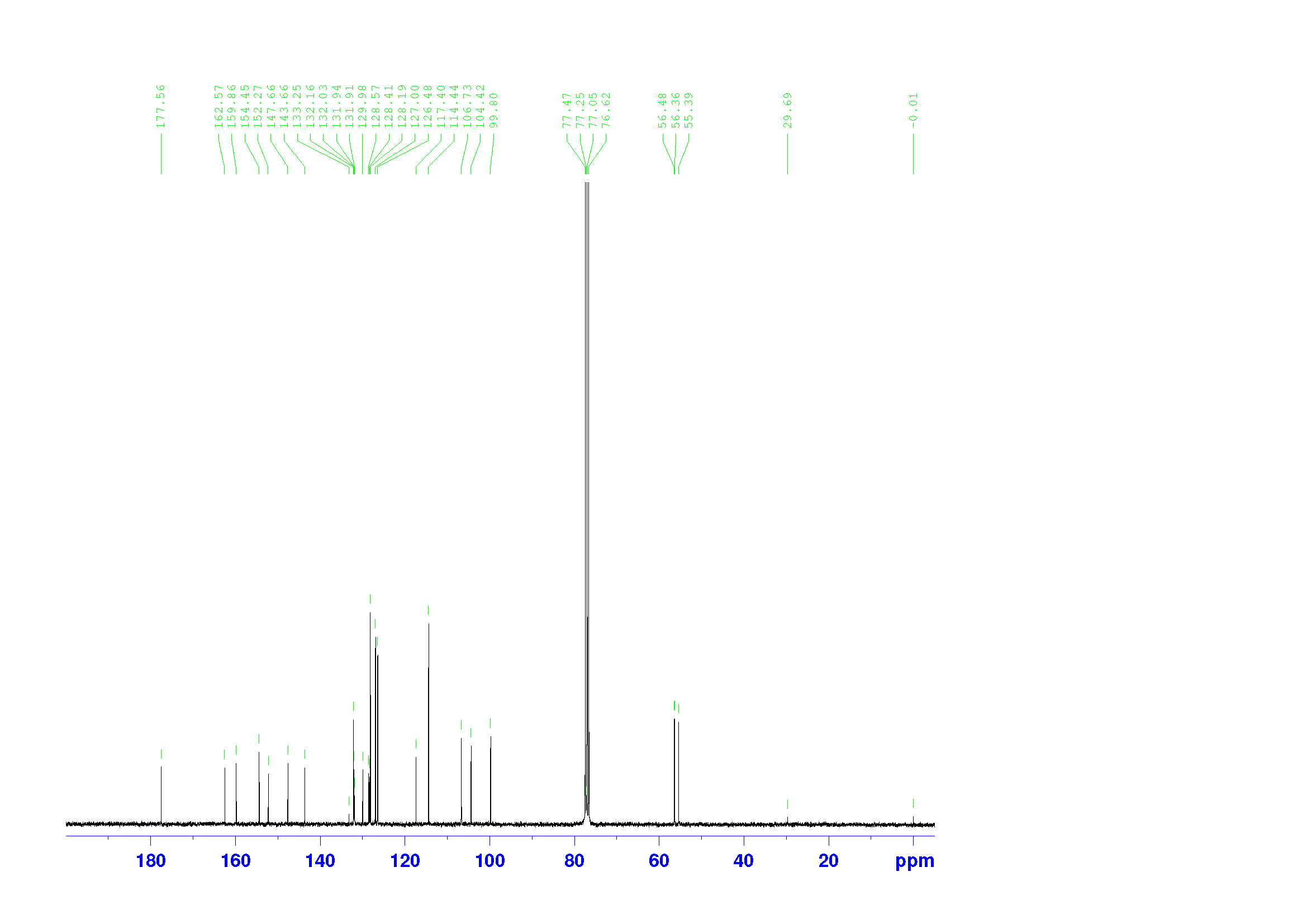


HRMS spectra of compound **9a**

^1^H NMR spectra of compound **9b** measured in CDCl_3_ at 300 MHz

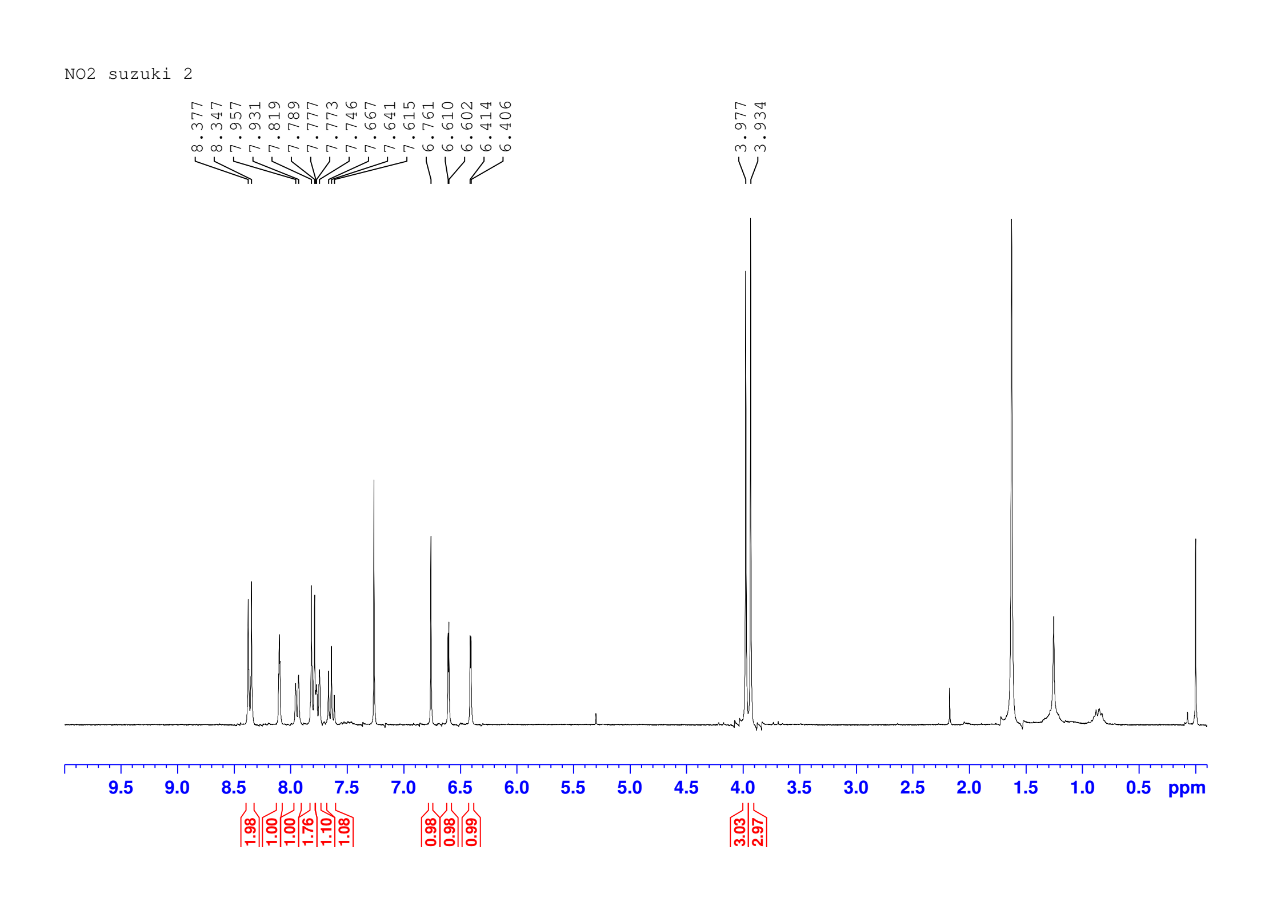


^1^H NMR spectra of compound **9c** measured in CDCl_3_ at 300 MHz

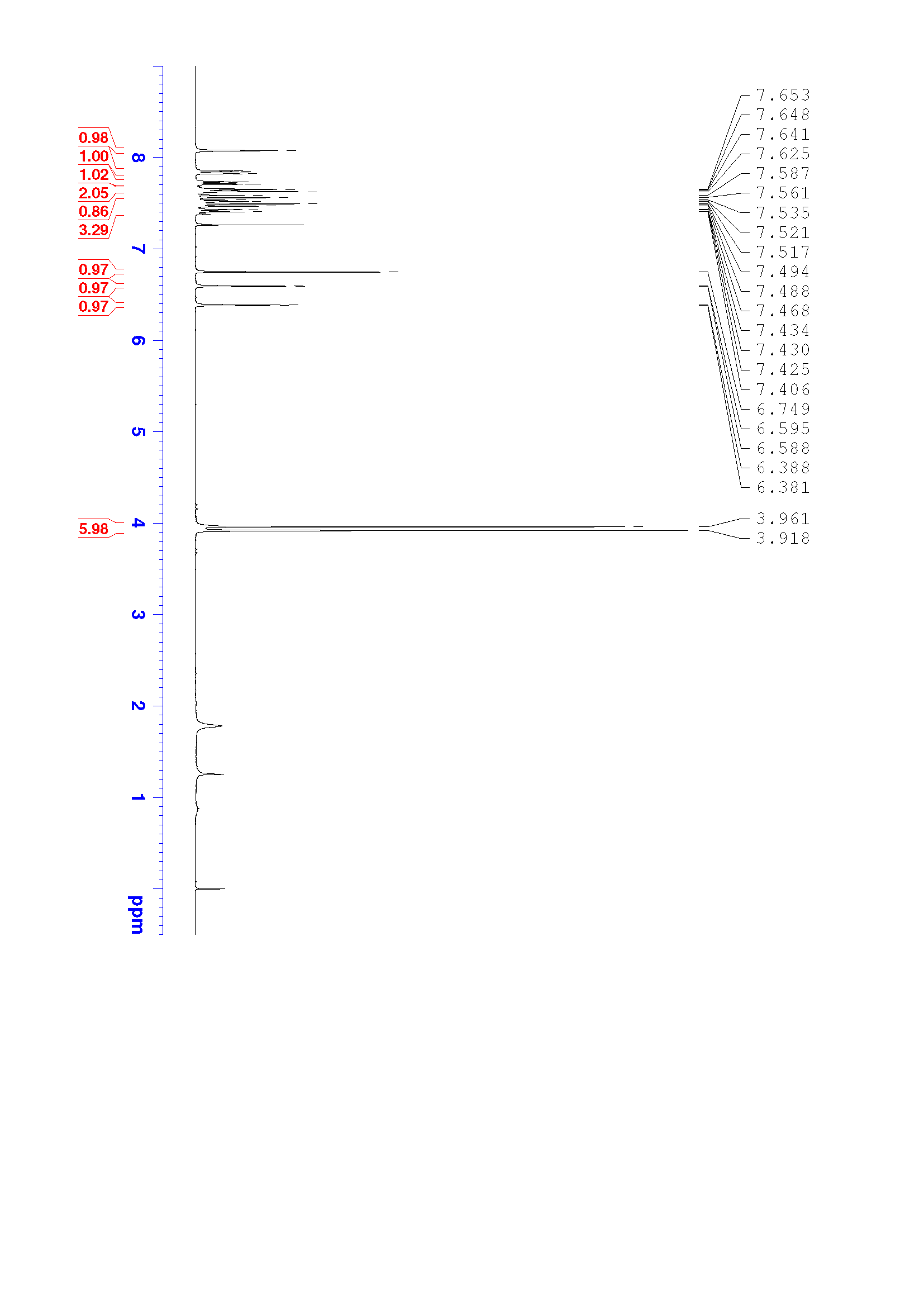


^13^C NMR spectra of compound **9c** measured in CDCl_3_ at 75 MHz

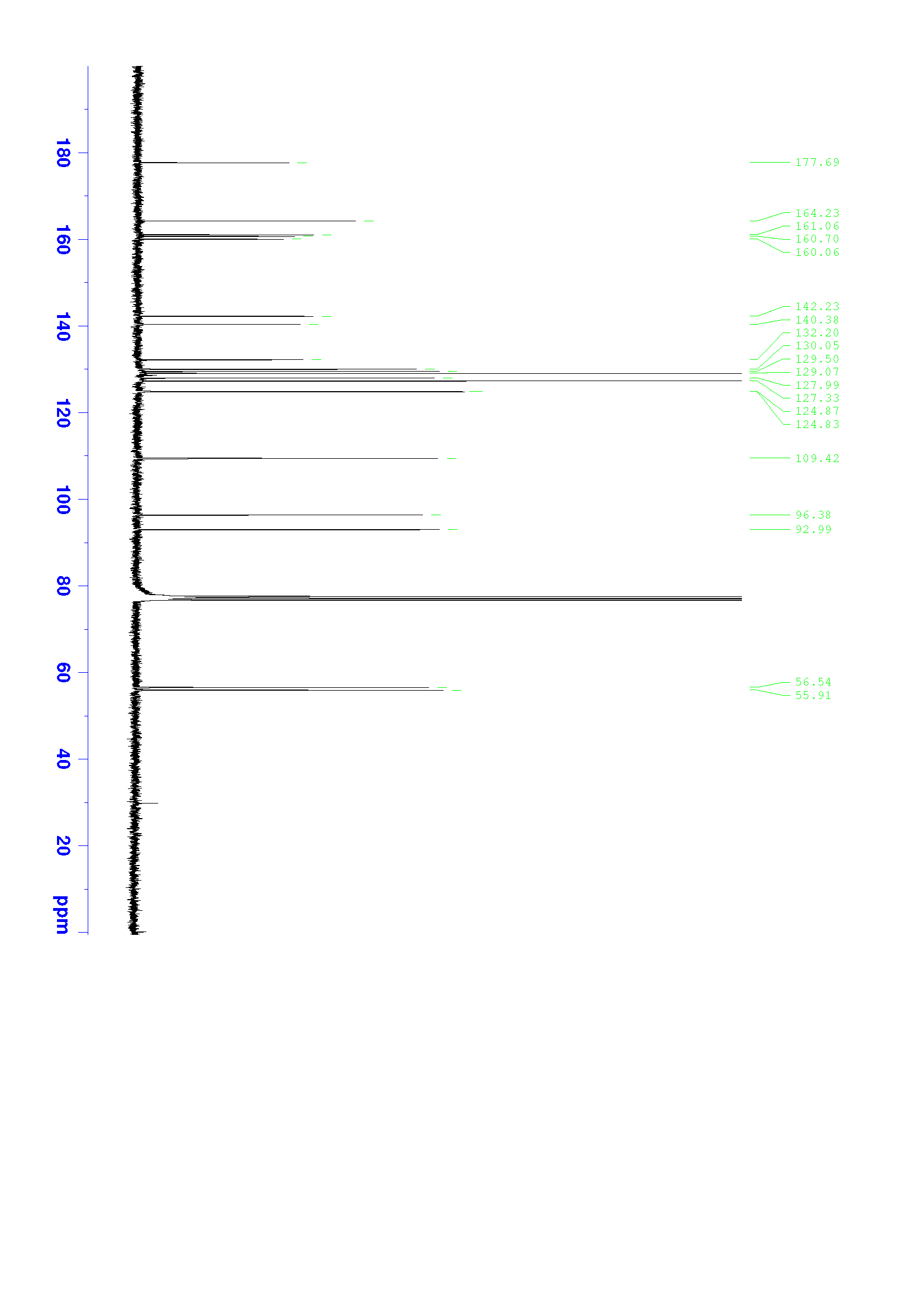


HRMS spectra of compound **9c**

^1^H NMR spectra of compound **9d** measured in CDCl_3_ at 300 MHz

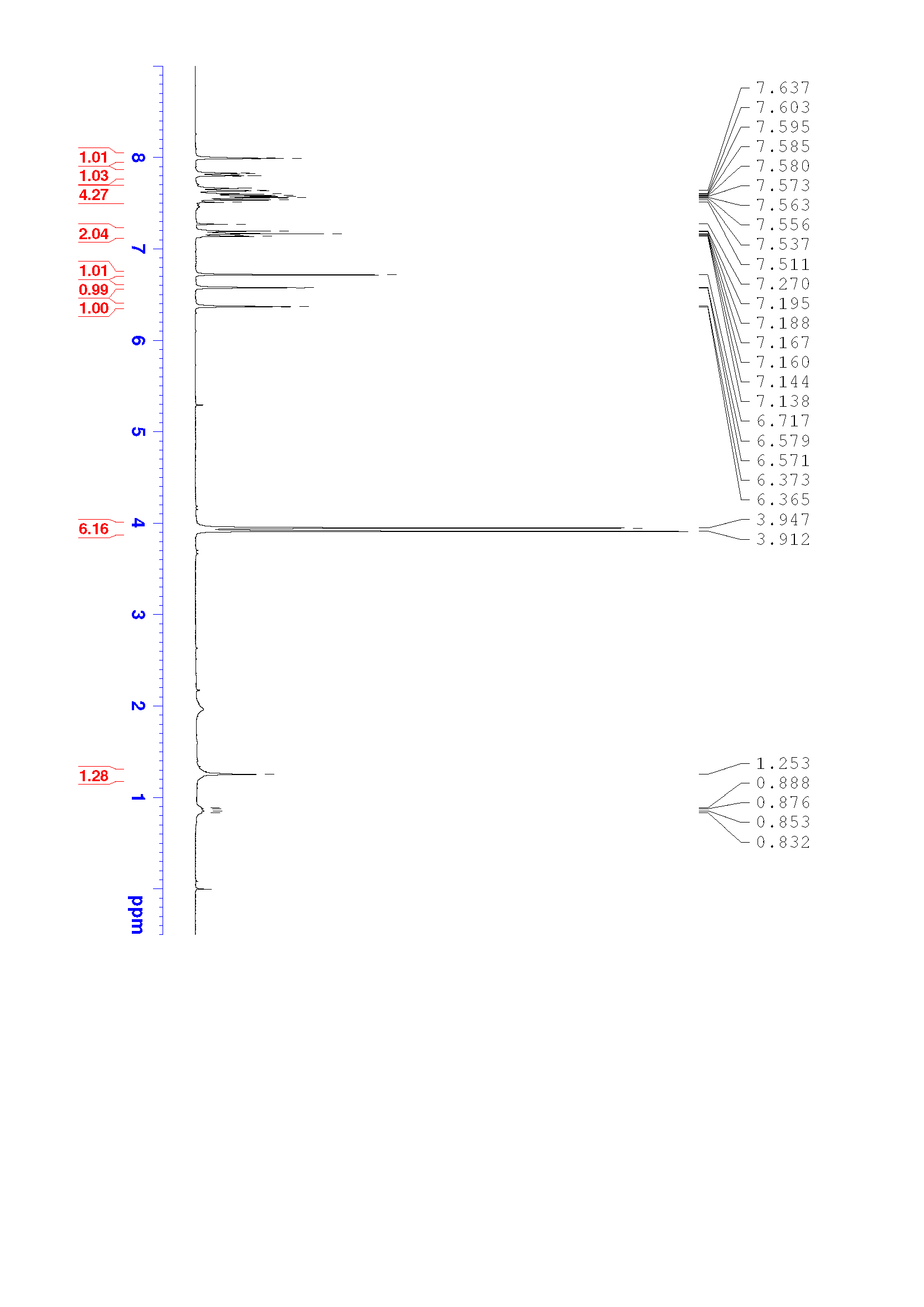


^13^C NMR spectra of compound **9d** measured in CDCl_3_ at 75 MHz

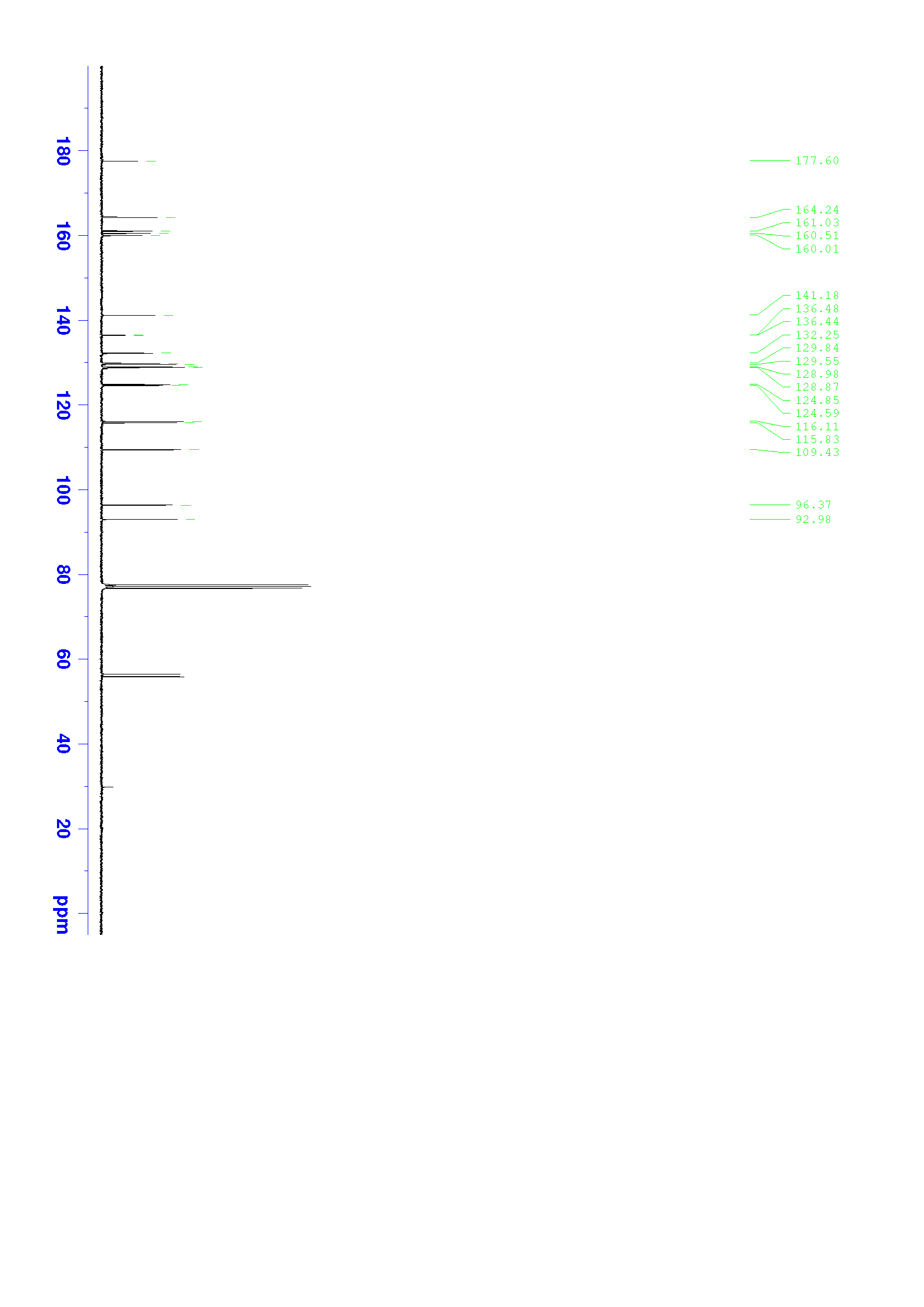


HRMS spectra of compound **9d**

^1^H NMR spectra of compound **9e** measured in CDCl_3_ at 300 MHz

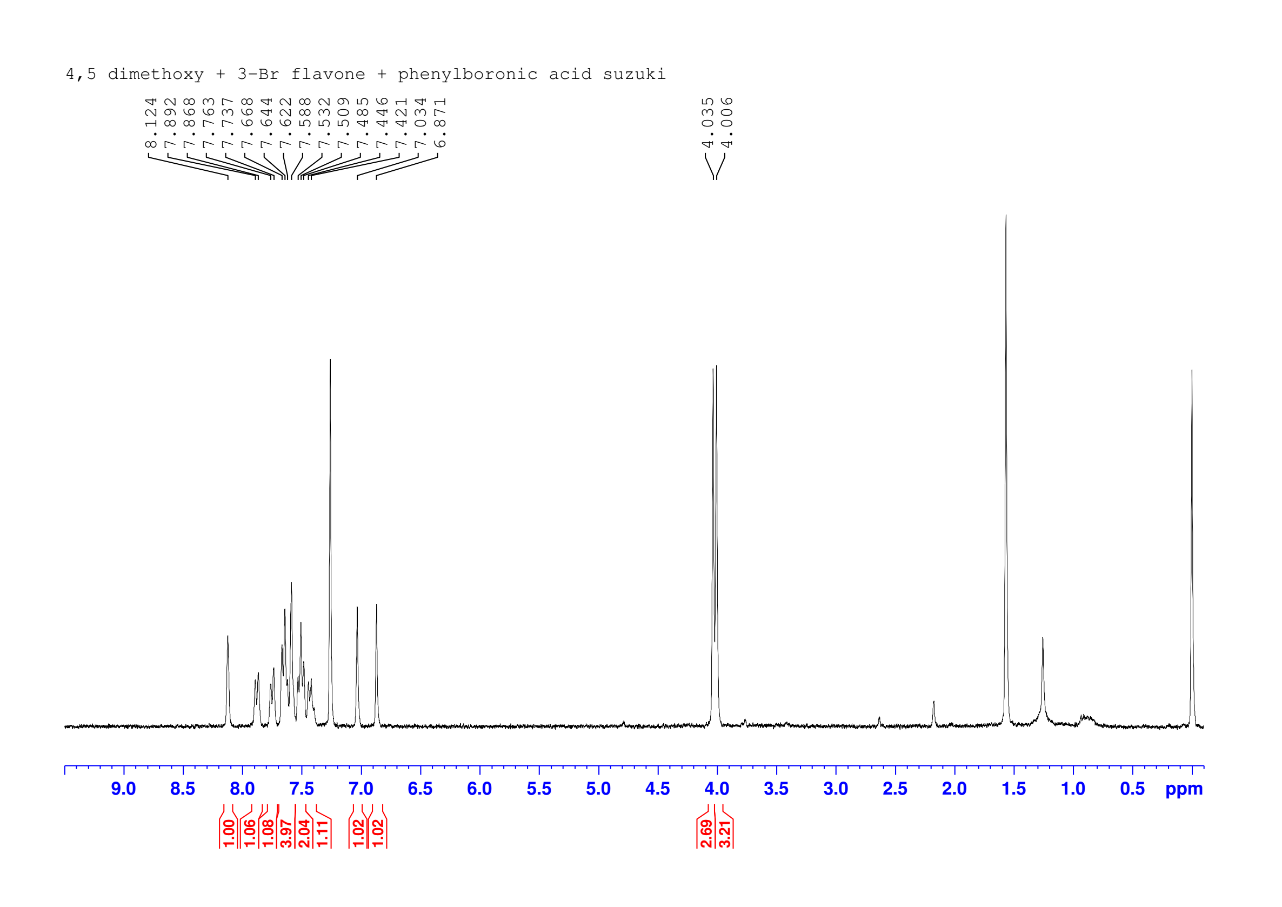


^13^C NMR spectra of compound **9e** measured in CDCl_3_ at 75 MHz

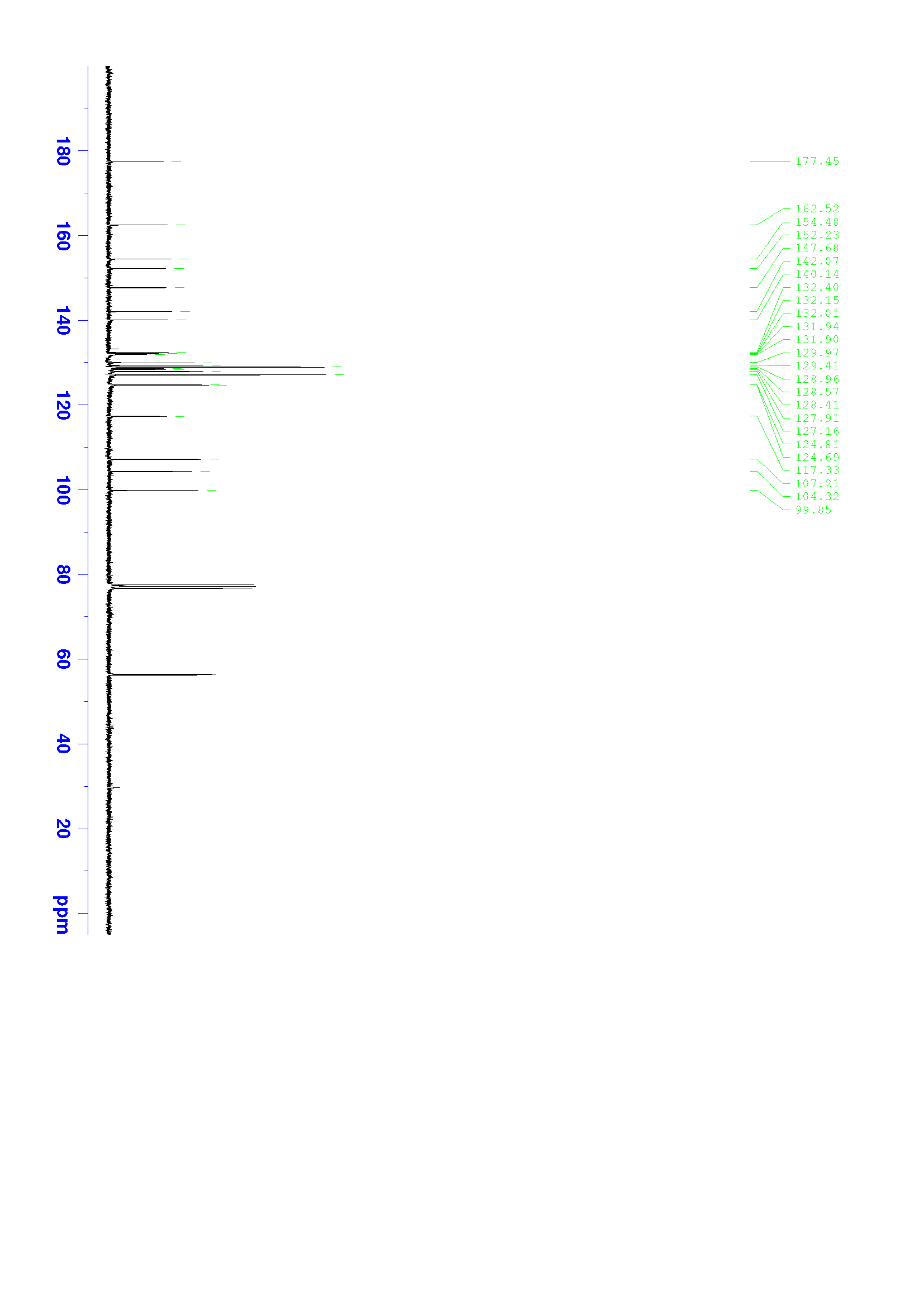


HRMS spectra of compound **9e**

^1^H NMR spectra of compound **9f** measured in CDCl_3_ at 300 MHz

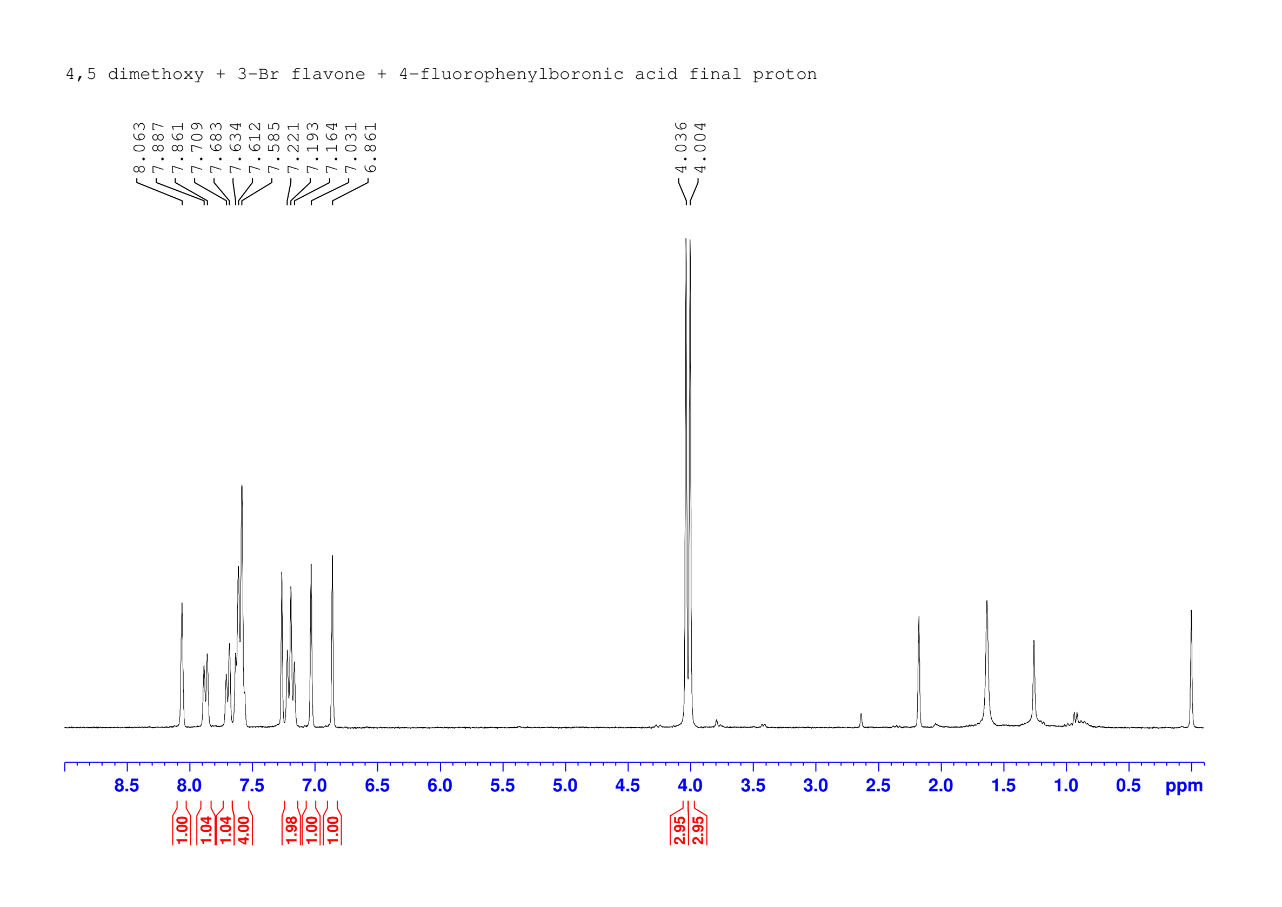


^13^C NMR spectra of compound **9f** measured in CDCl_3_ at 75 MHz

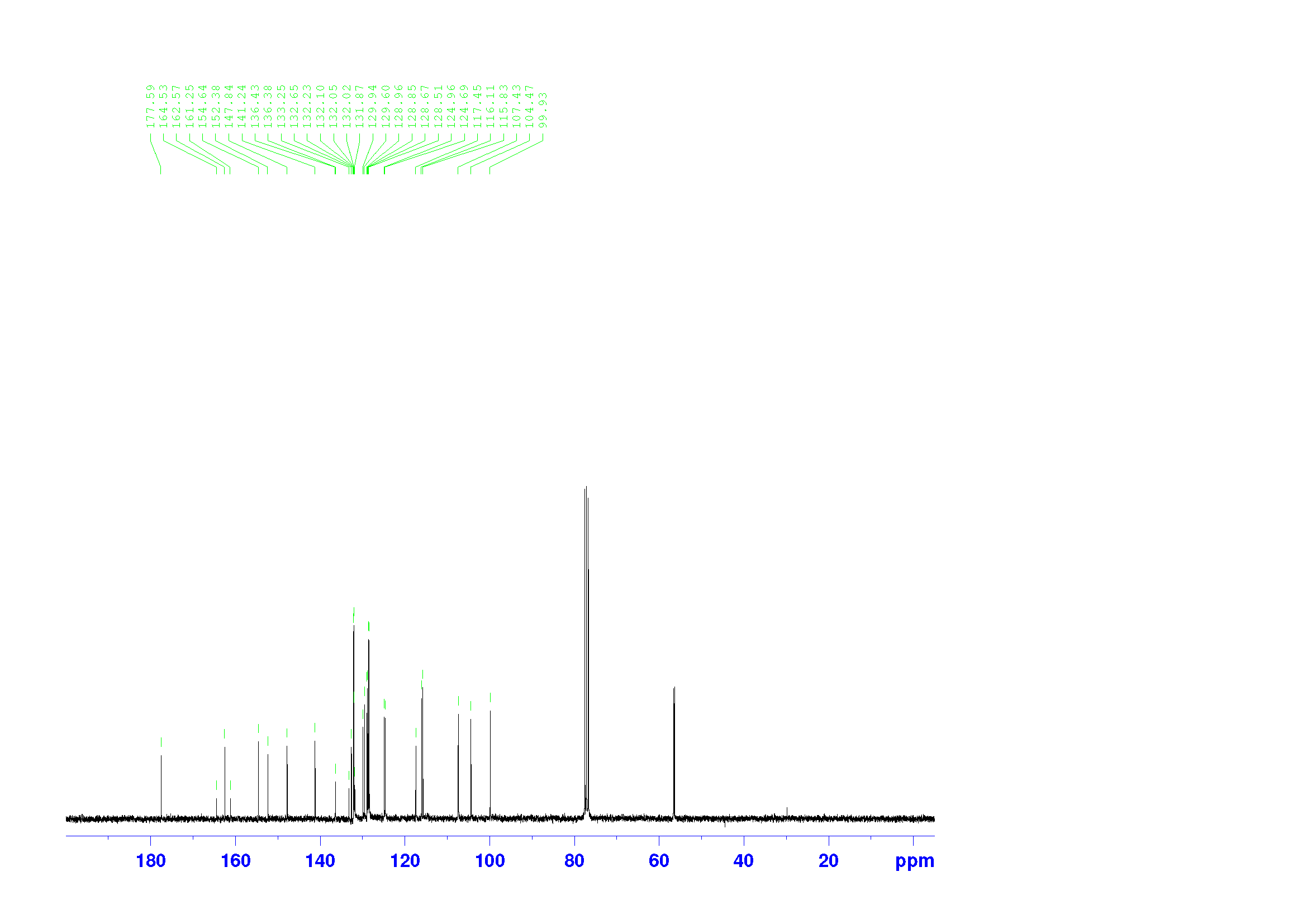


HRMS spectra of compound **9f**

^1^H NMR spectra of compound **9g** measured in CDCl_3_ at 300 MHz

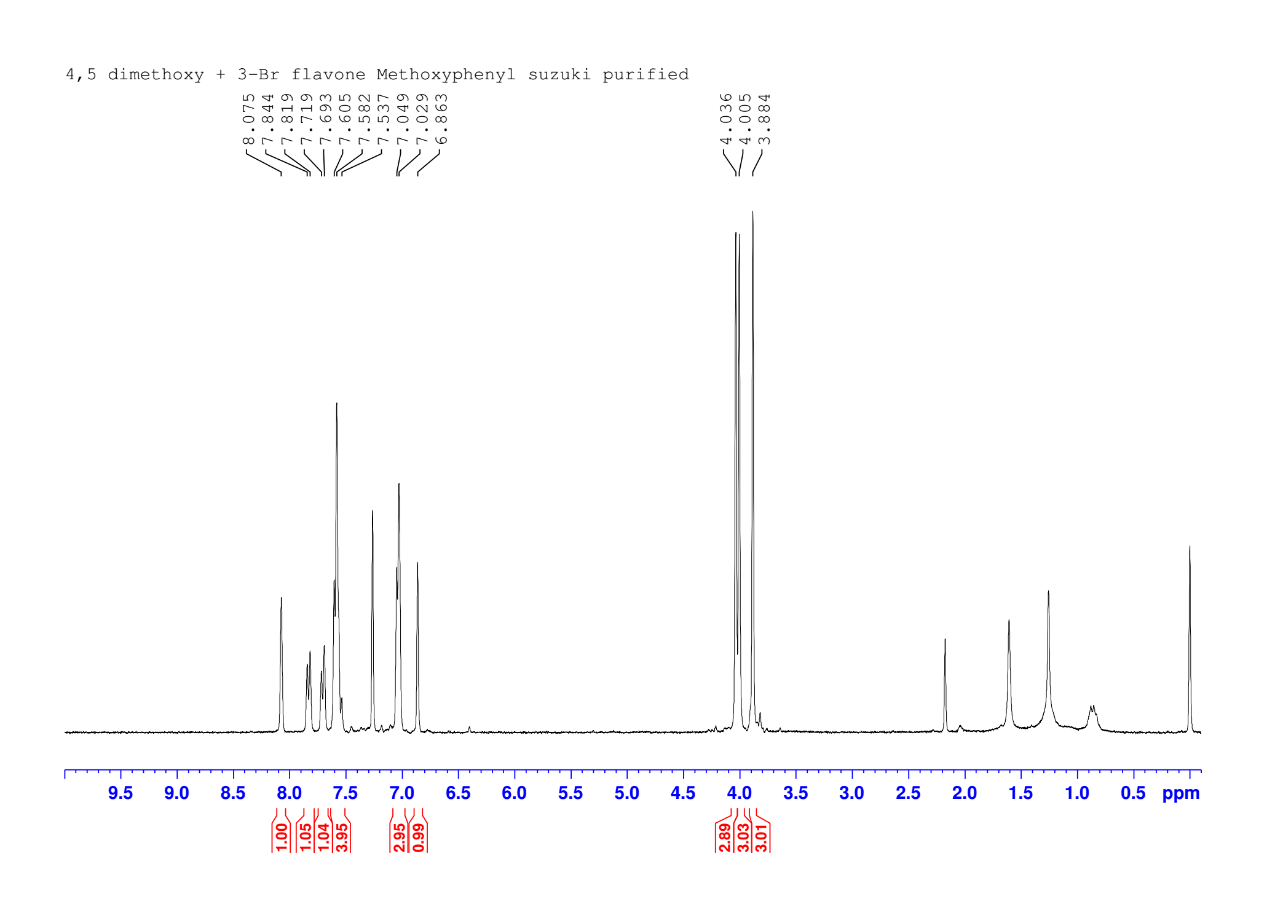


^13^C NMR spectra of compound **9g** measured in CDCl_3_ at 75 MHz

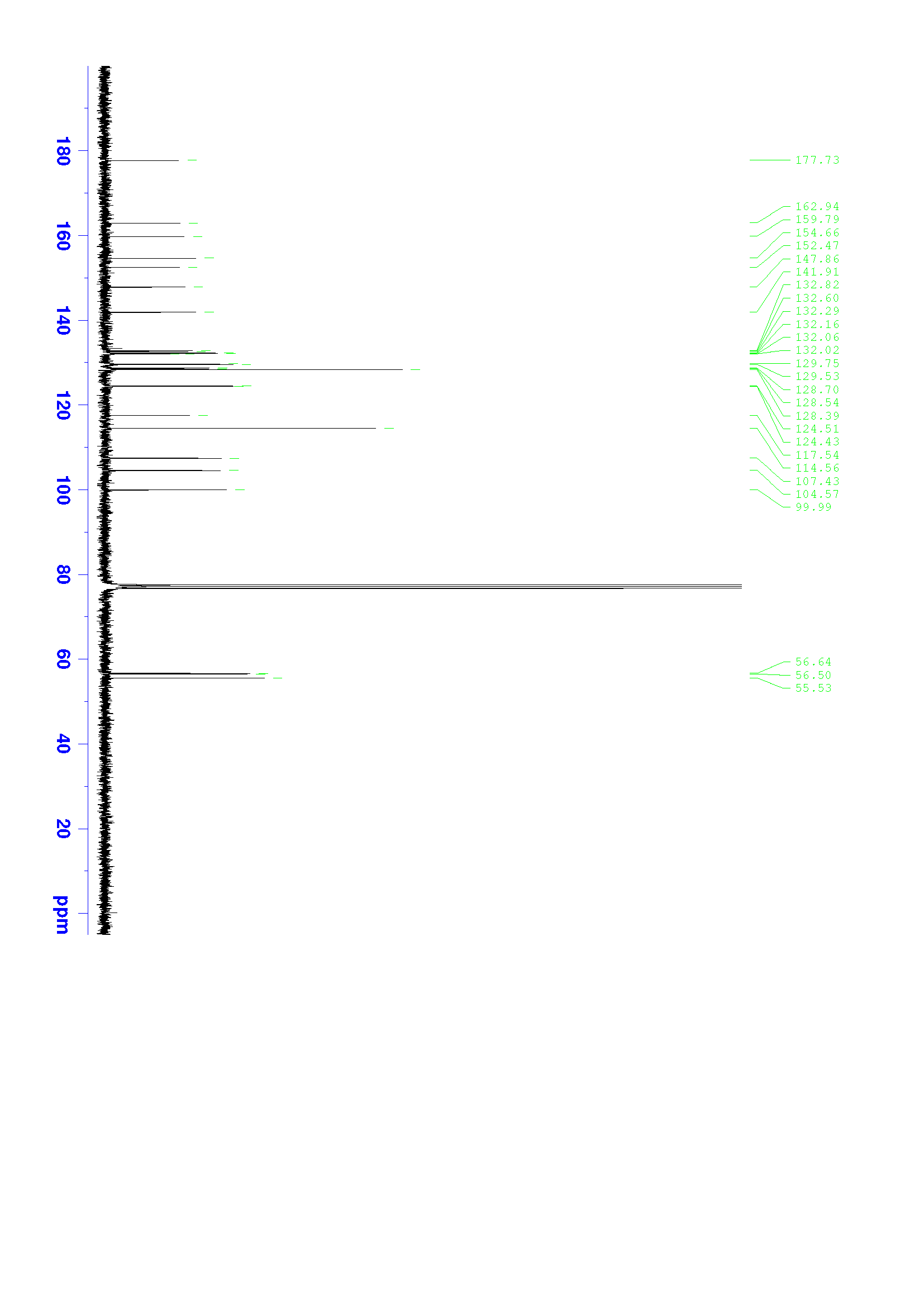


HRMS spectra of compound **9g**

^1^H NMR spectra of compound **9h** measured in CDCl_3_ at 300 MHz

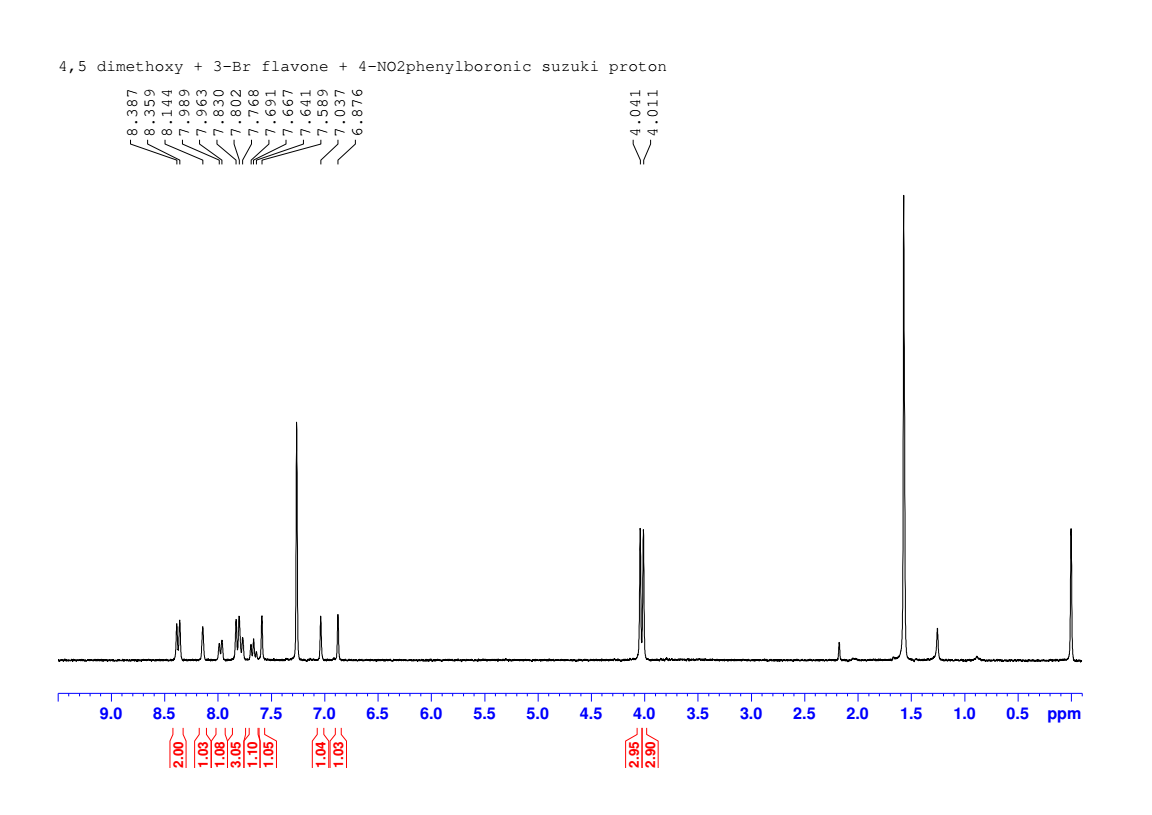


^1^H NMR spectra of compound **9i** measured in CDCl_3_ at 300 MHz

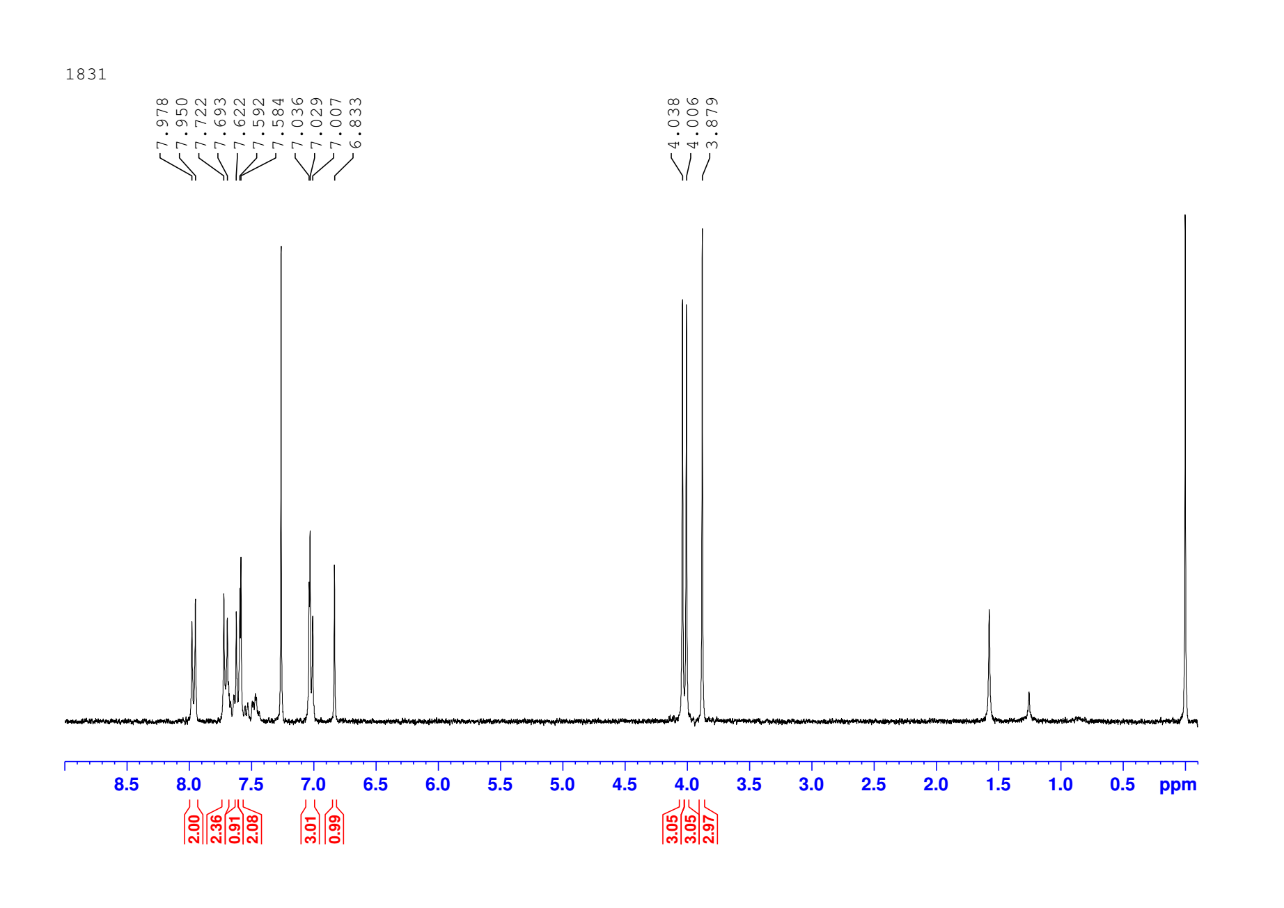


HRMS spectra of compound **9i**

^1^H NMR spectra of compound **10a** measured in tetrahydrofuran-*d*_8_ at 300 MHz

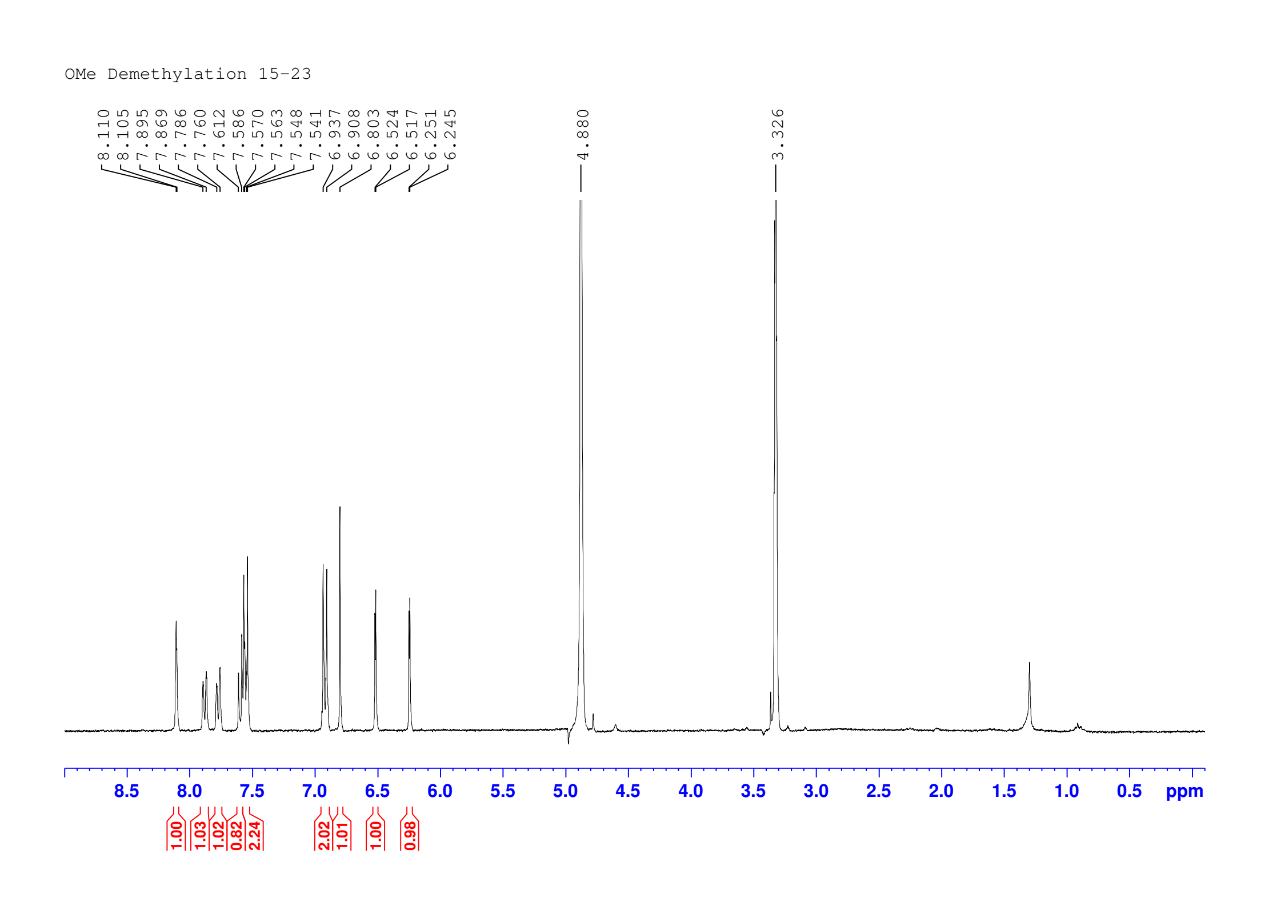


^13^C NMR spectra of compound **10a** measured in tetrahydrofuran-*d*_8_ at 75 MHz

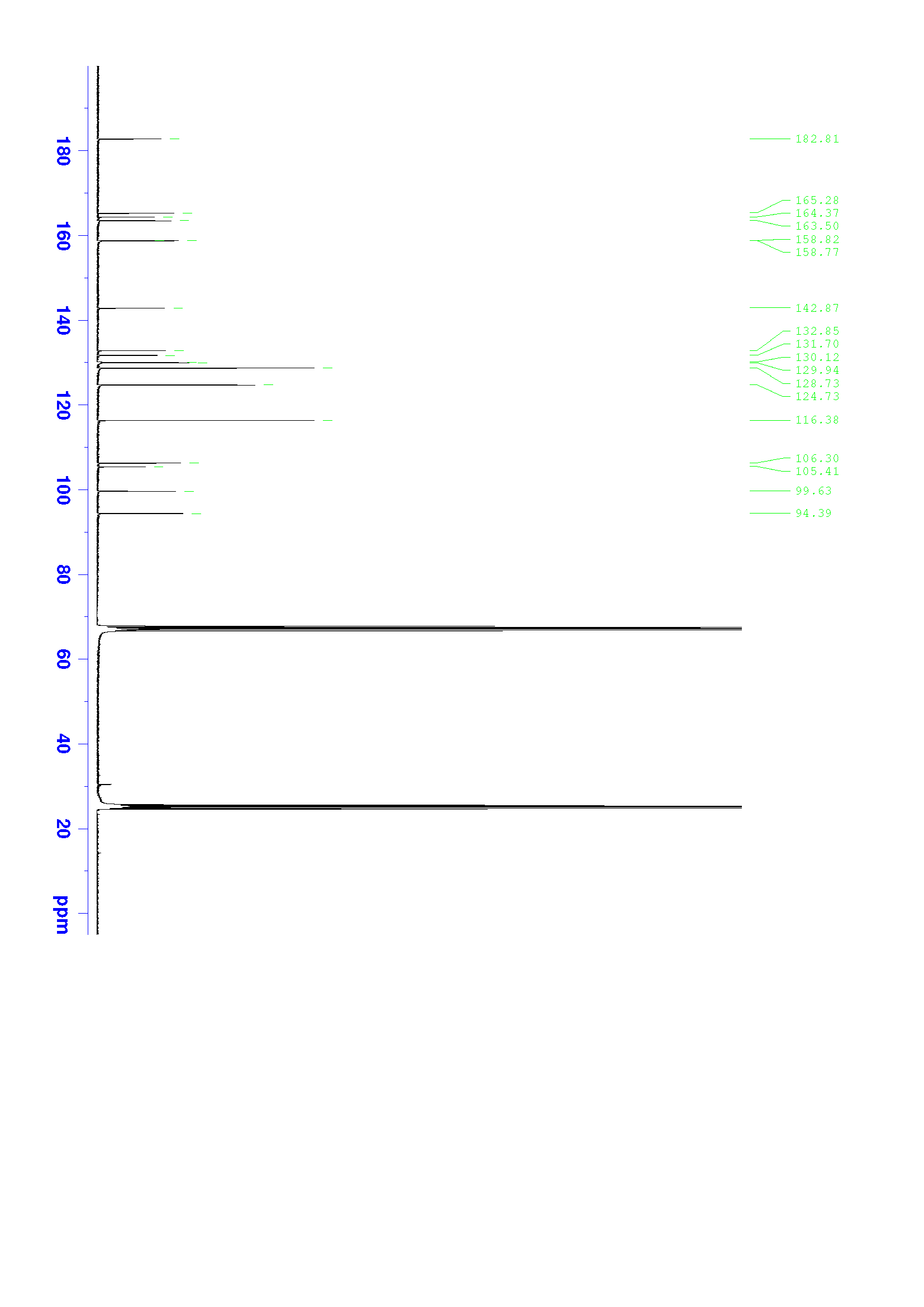


HRMS spectra of compound **10a**

^1^H NMR spectra of compound **10b** measured in CDCl_3_ at 300 MHz

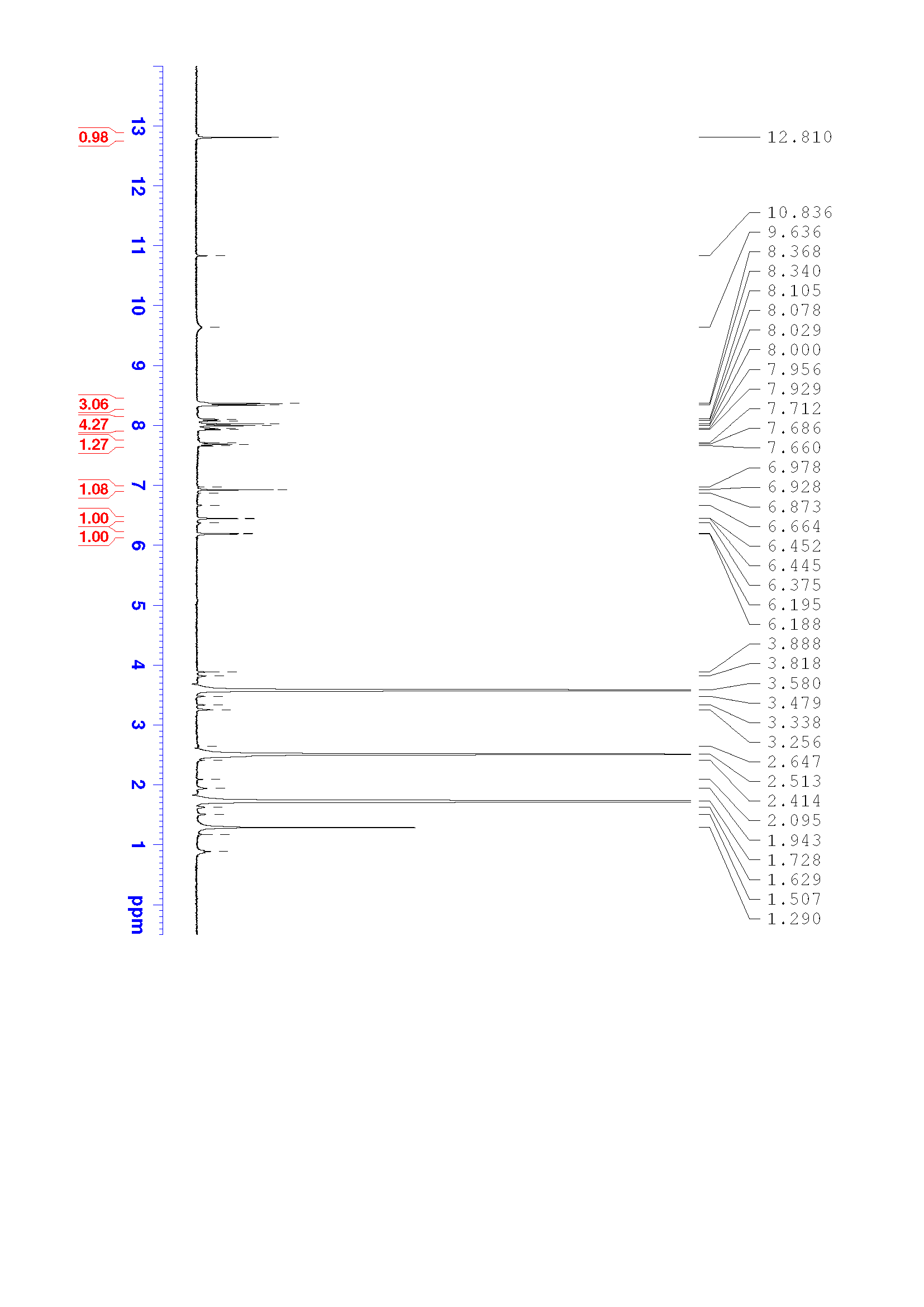


^13^C NMR spectra of compound **10b** measured in tetrahydrofuran-*d*_8_ at 75 MHz

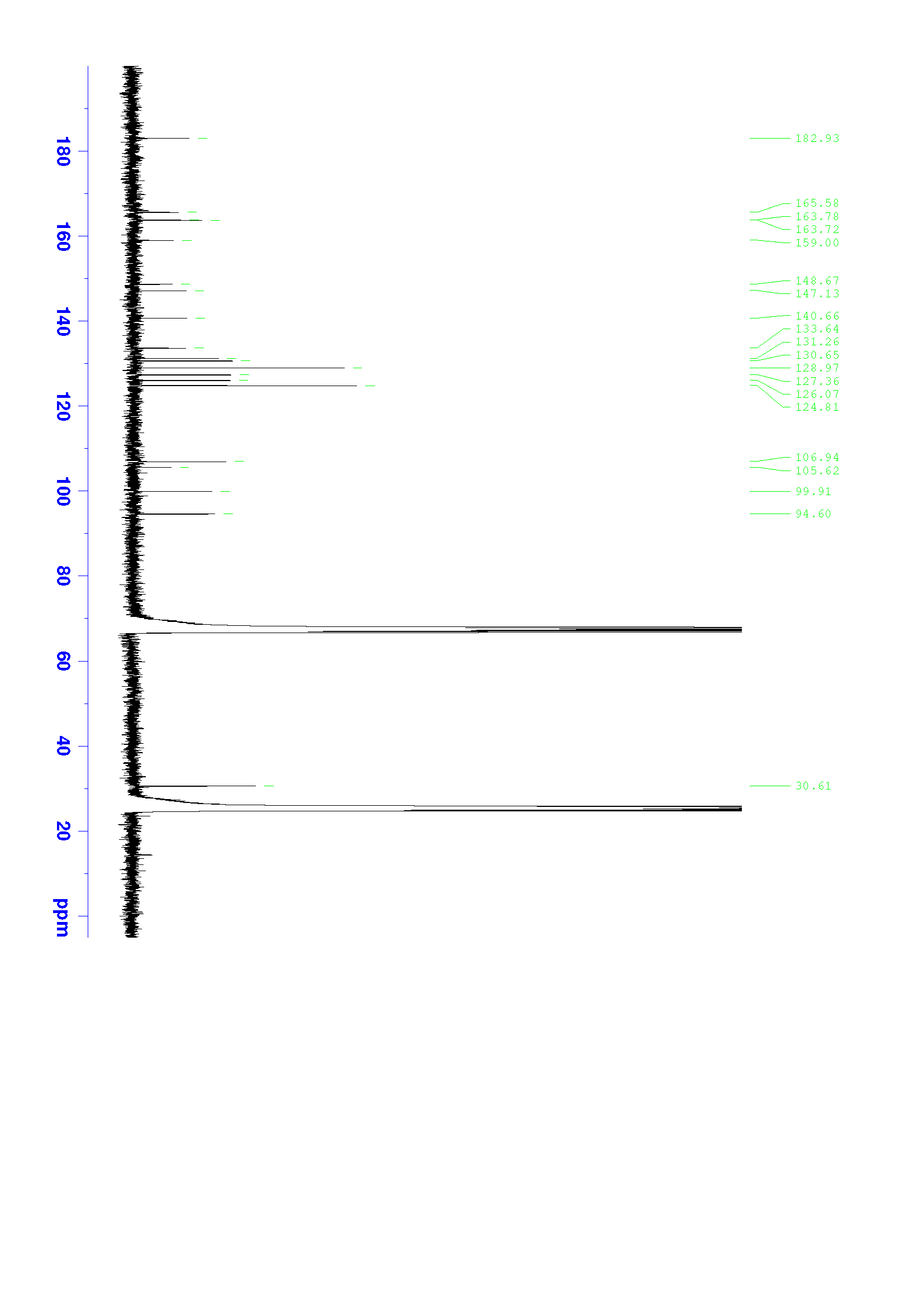


HRMS spectra of compound **10b**

^1^H NMR spectra of compound **10c** measured in tetrahydrofuran-*d*_8_at 300 MHz

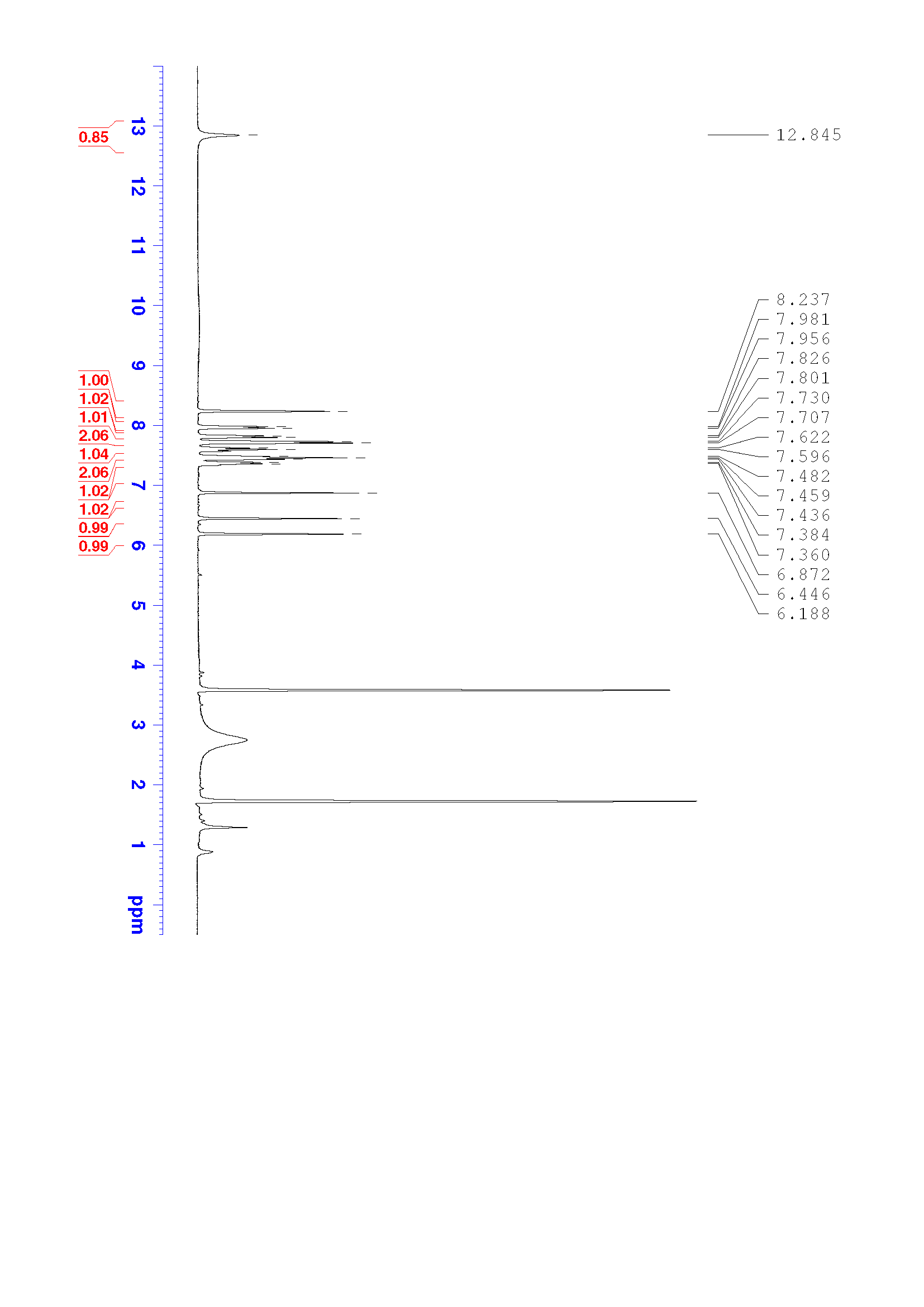


^13^C NMR spectra of compound **10c** measured in tetrahydrofuran-*d*_8_ at 75 MHz

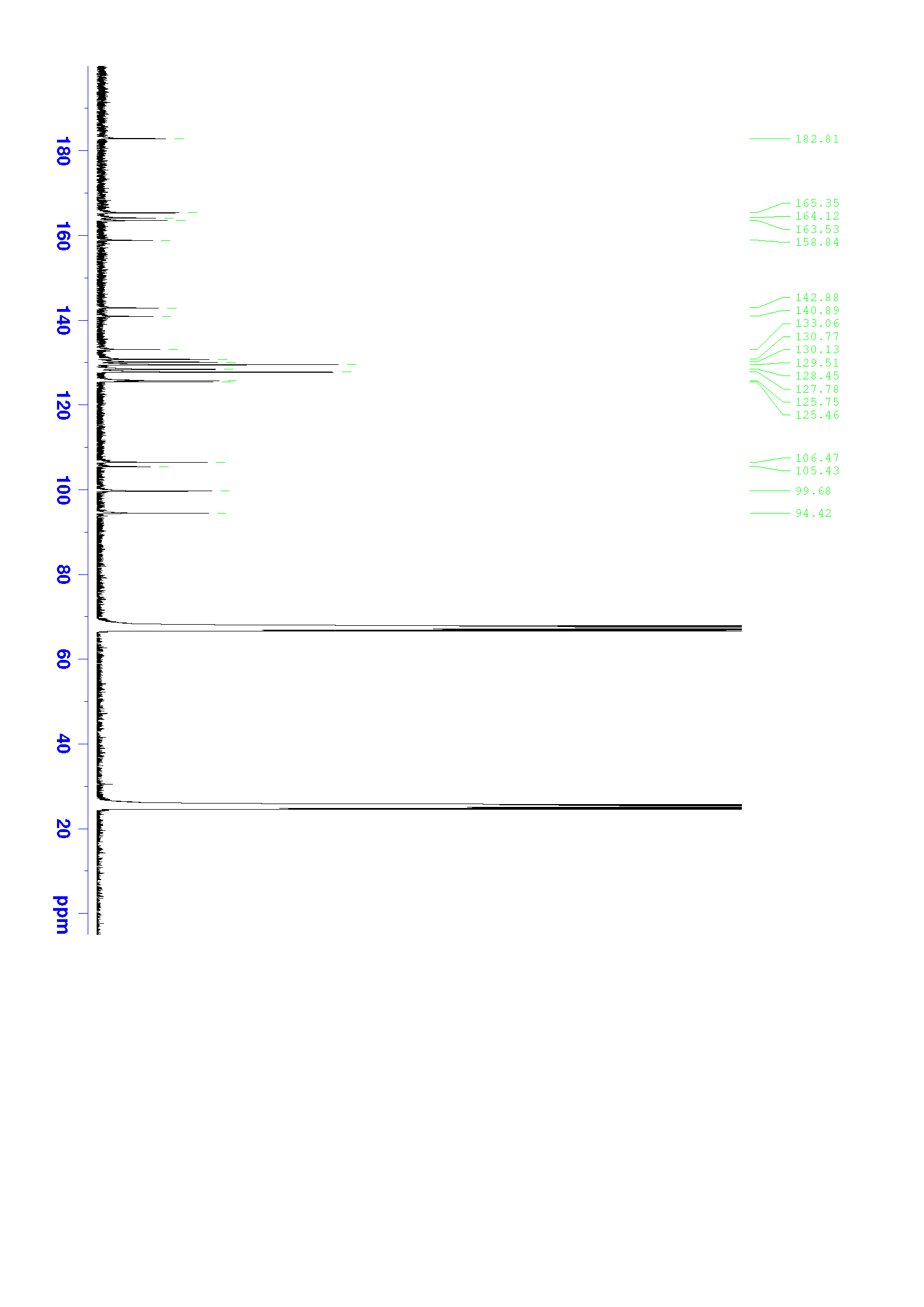


HRMS spectra of compound **10c**

^1^H NMR spectra of compound **10d** measured in tetrahydrofuran-*d*_8_ at 300 MHz

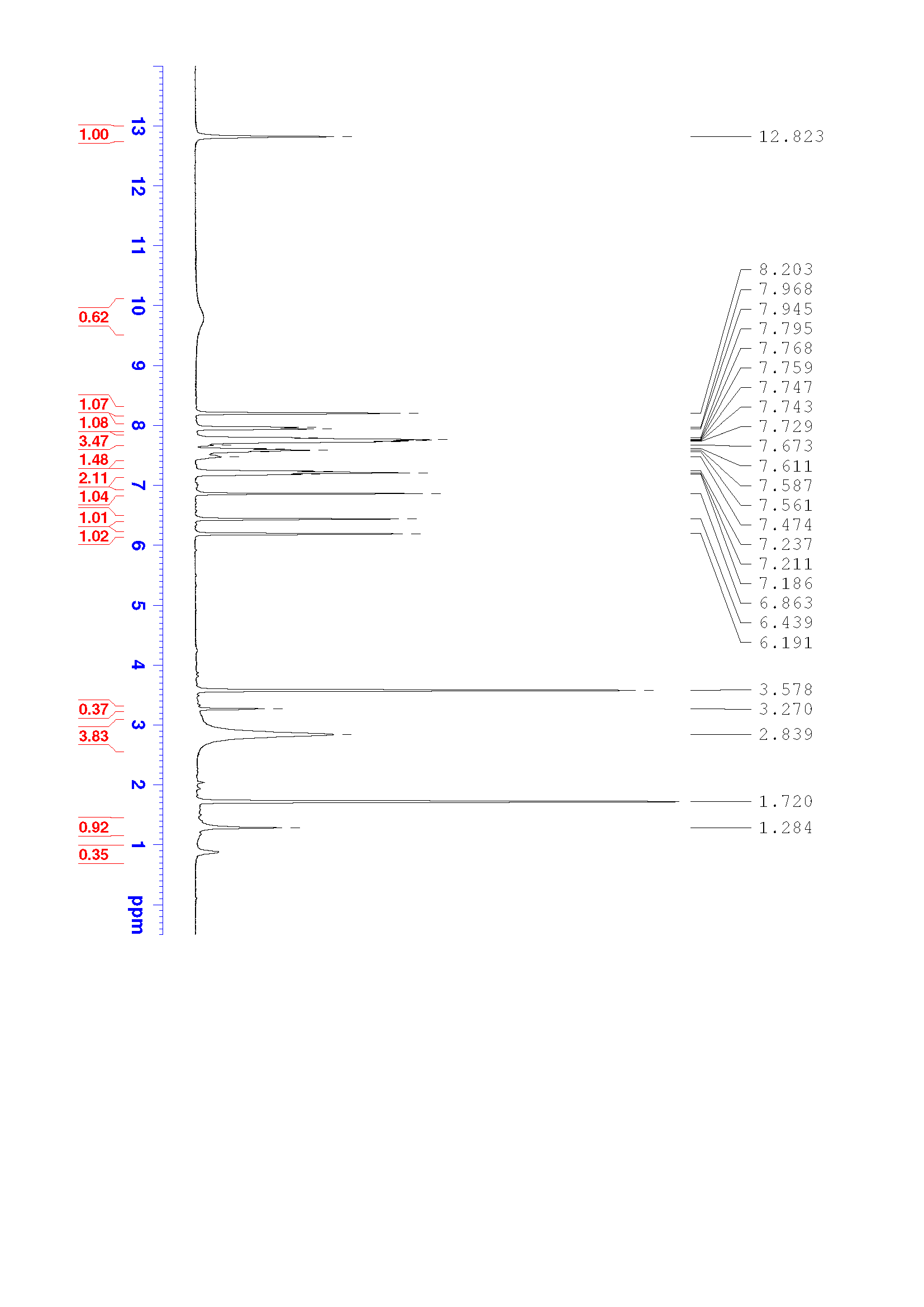


^13^C NMR spectra of compound **10d** measured in tetrahydrofuran-*d*_8_ at 75 MHz

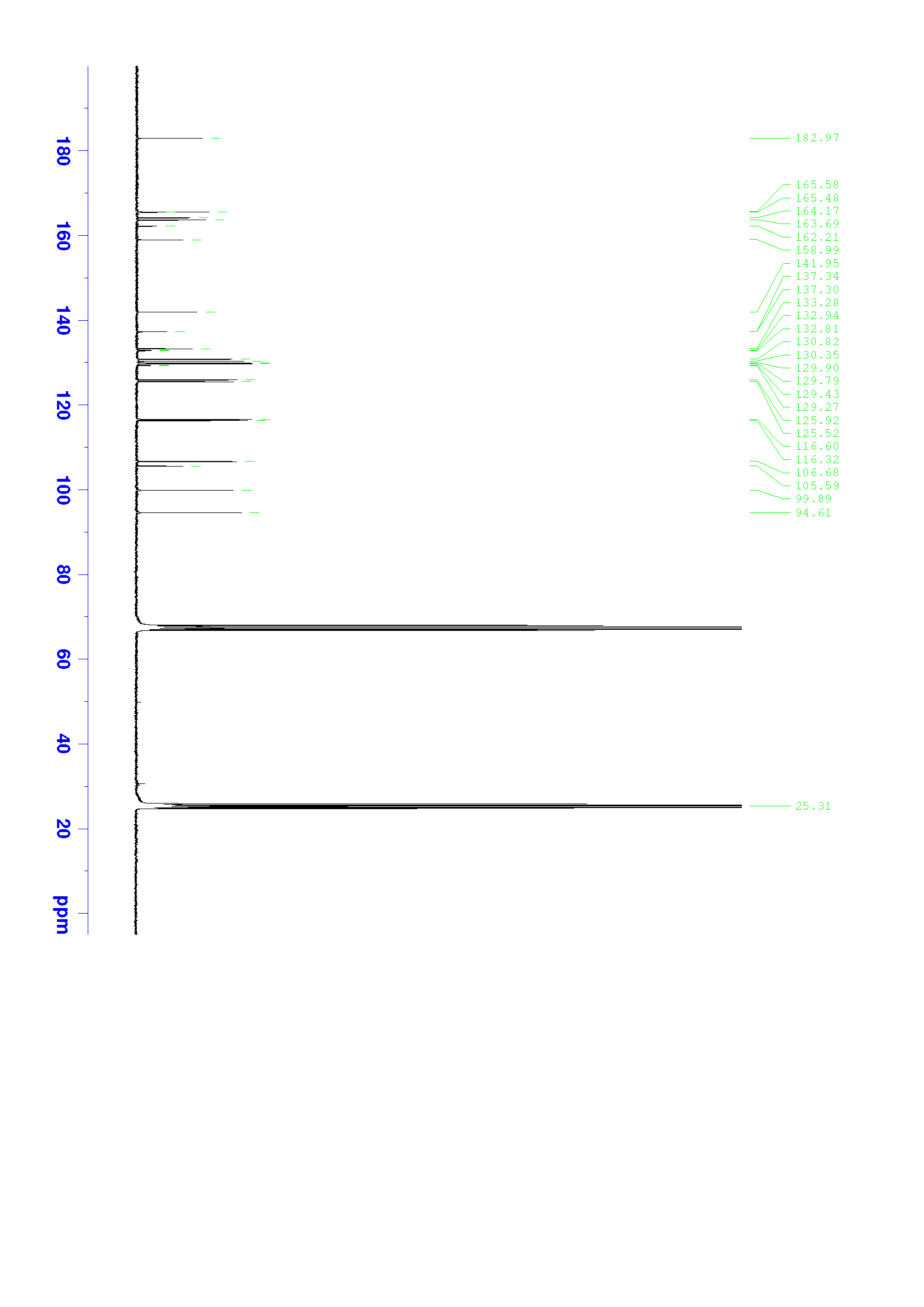


HRMS spectra of compound **10d**

^1^H NMR spectra of compound **10e** measured in tetrahydrofuran-*d*_8_ at 300 MHz

^
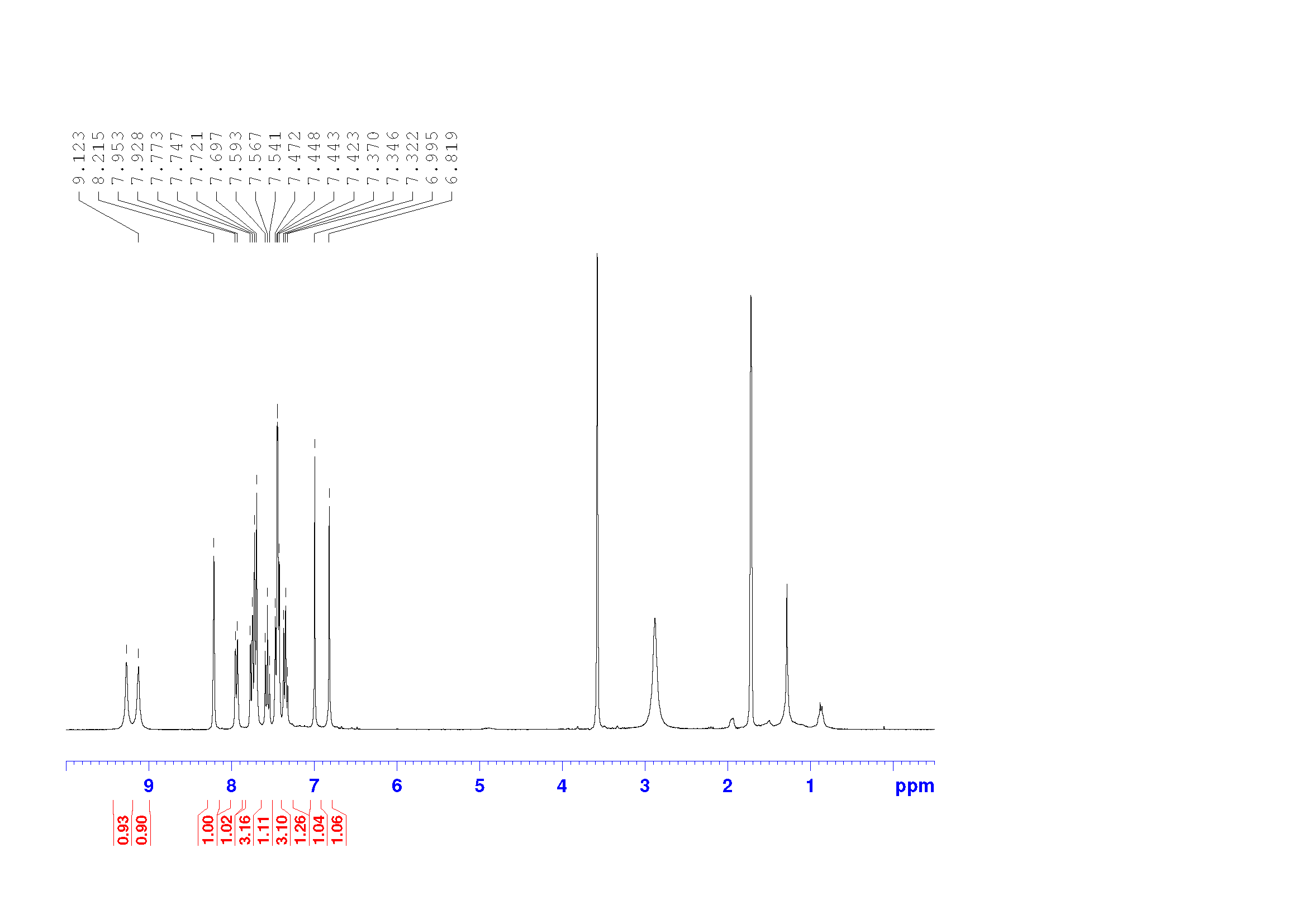
^

^13^C NMR spectra of compound **10e** measured in tetrahydrofuran-*d*_8_ at 75 MHz

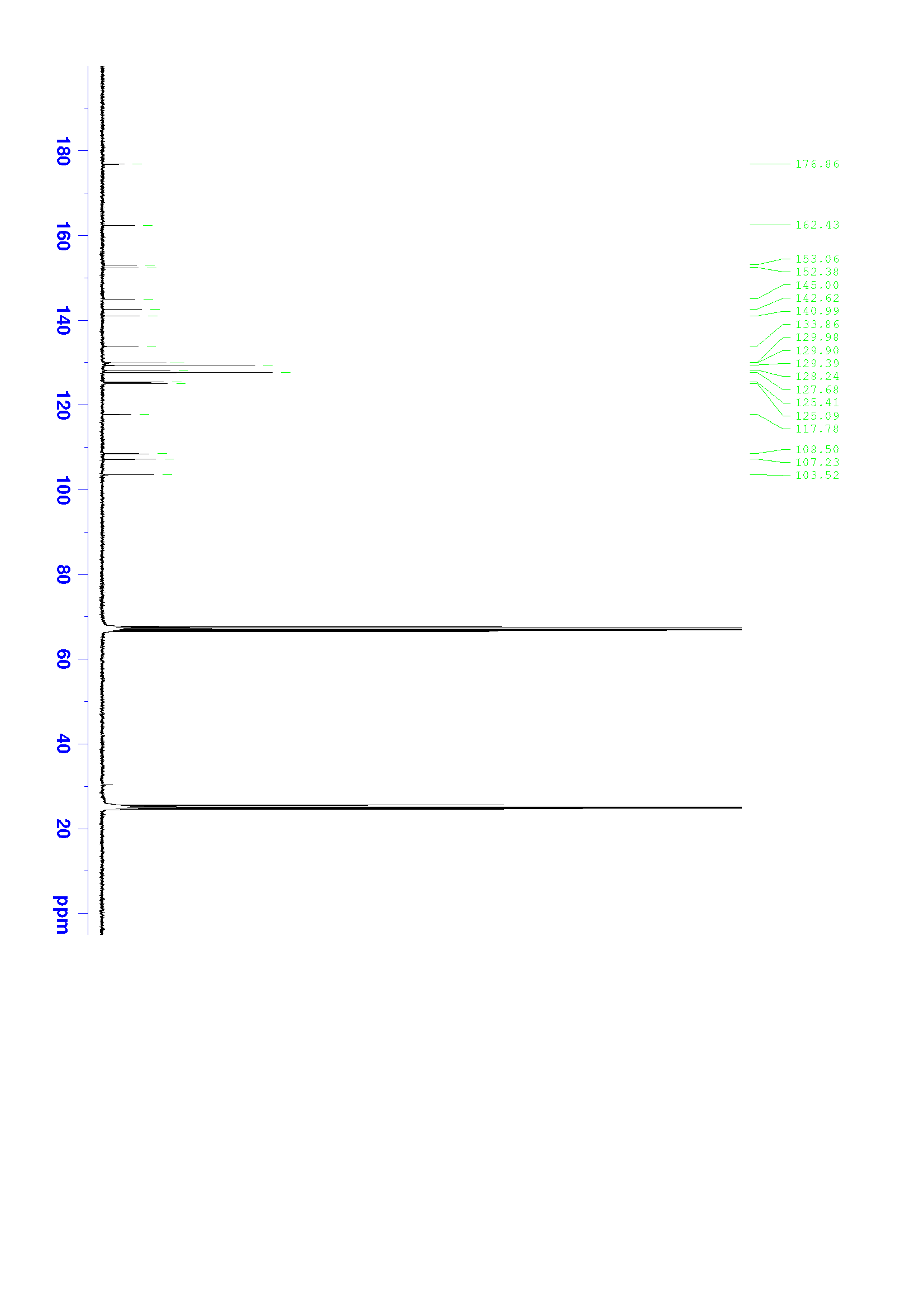


HRMS spectra of compound **10e**

^1^H NMR spectra of compound **10f** measured in tetrahydrofuran-*d*_8_ at 300 MHz


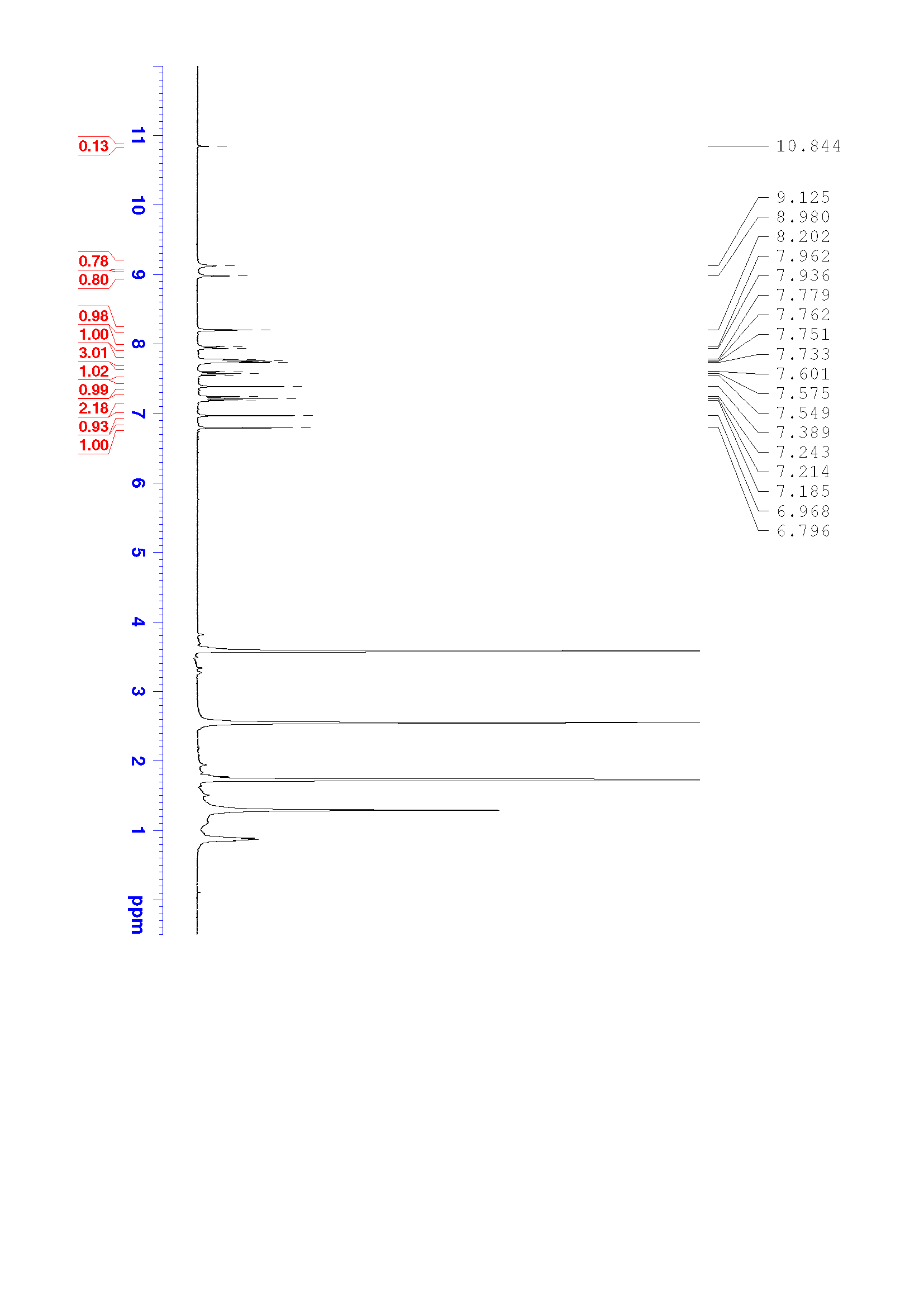


^13^C NMR spectra of compound **10f** measured in tetrahydrofuran-*d*_8_ at 75 MHz


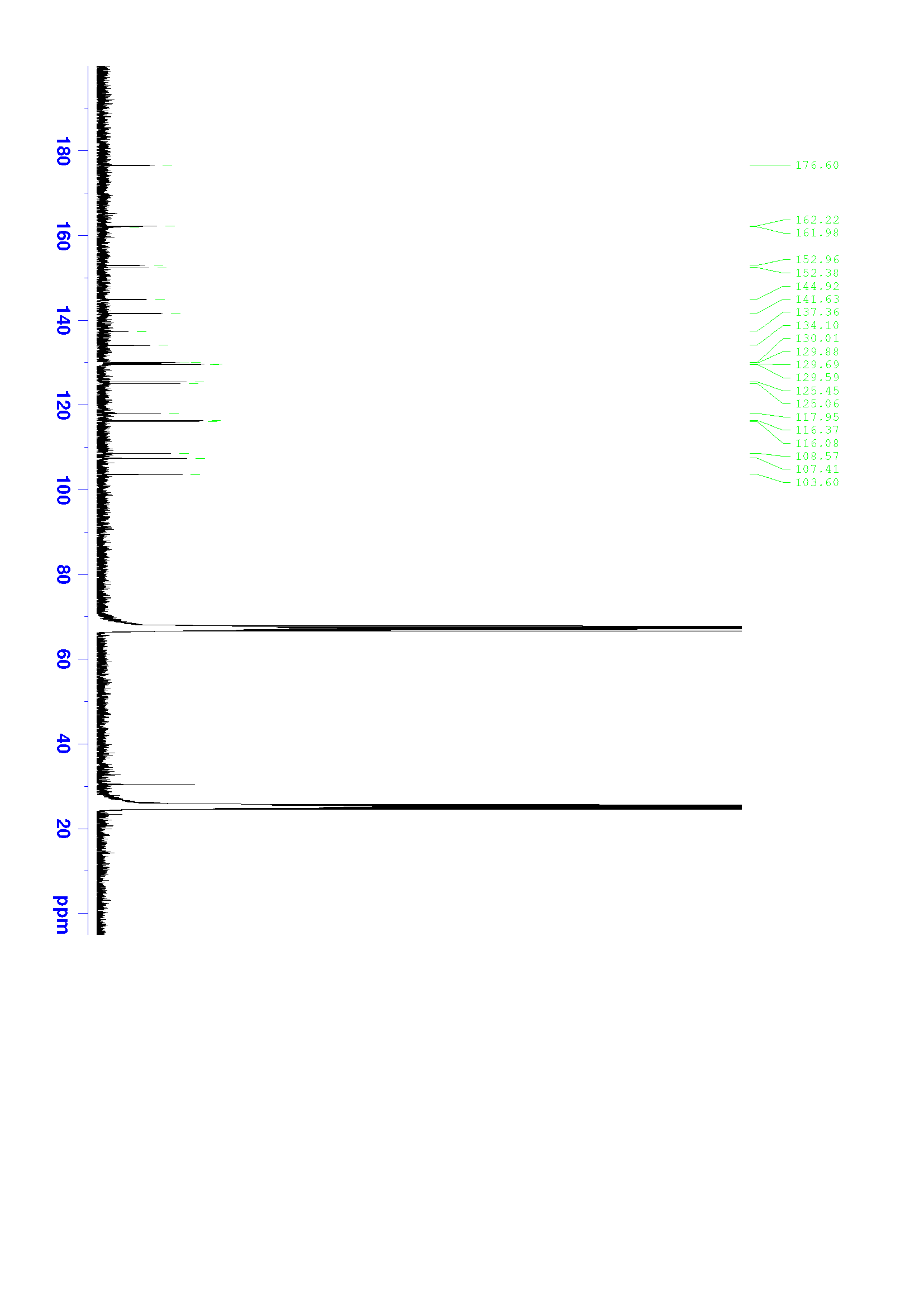


HRMS spectra of compound **10f**

^1^H NMR spectra of compound **10g** measured in CD_3_OD_3_ at 300 MHz


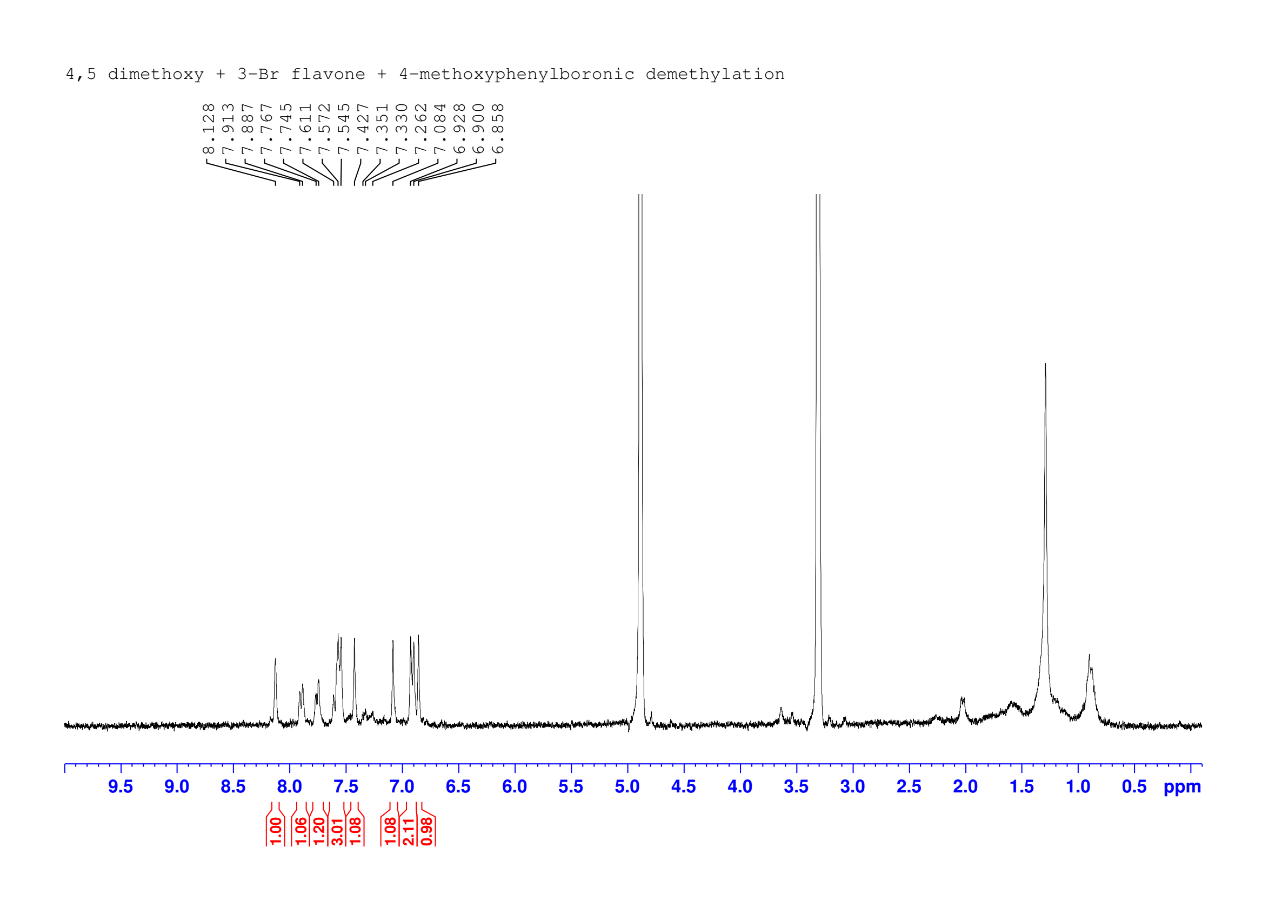


HRMS spectra of compound **10g**

^1^H NMR spectra of compound **10h** measured in CD_3_OD_3_ at 300 MHz


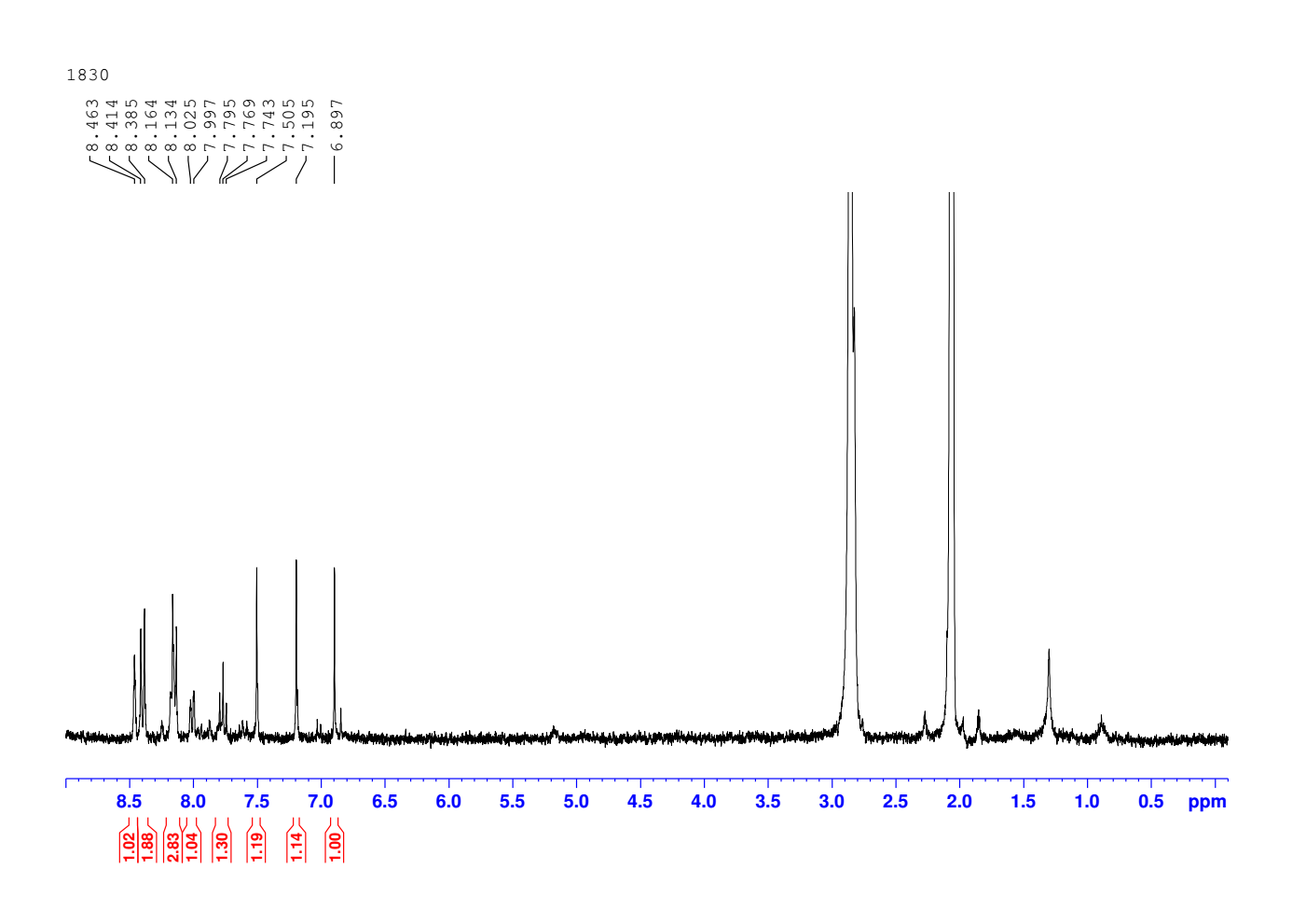


HRMS spectra of compound **10h**

^1^H NMR spectra of compound **10i** measured in CD_3_OD_3_ at 300 MHz


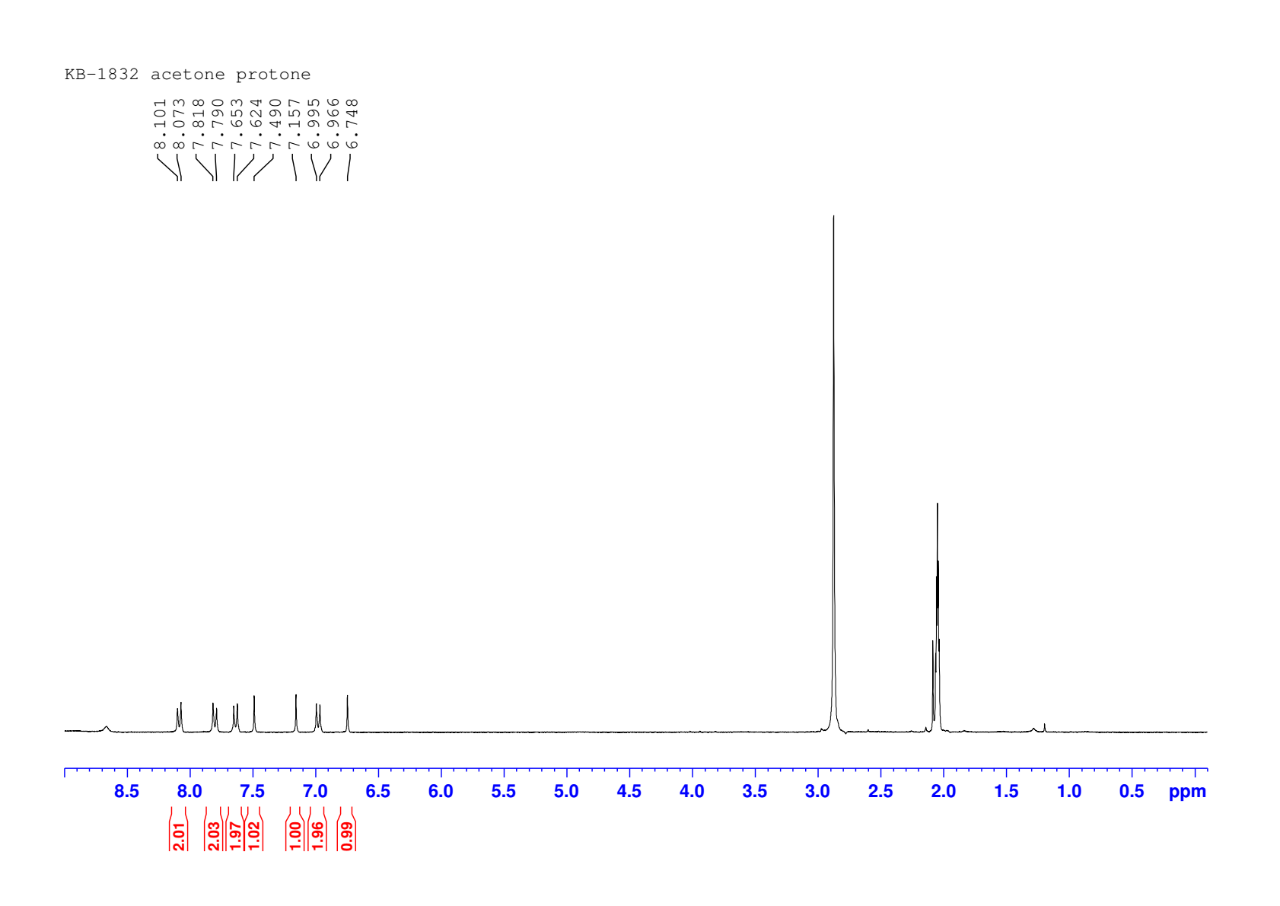


HRMS spectra of compound **10i**

^1^H NMR spectra of compound **11a** measured in CD_3_OD at 300 MHz


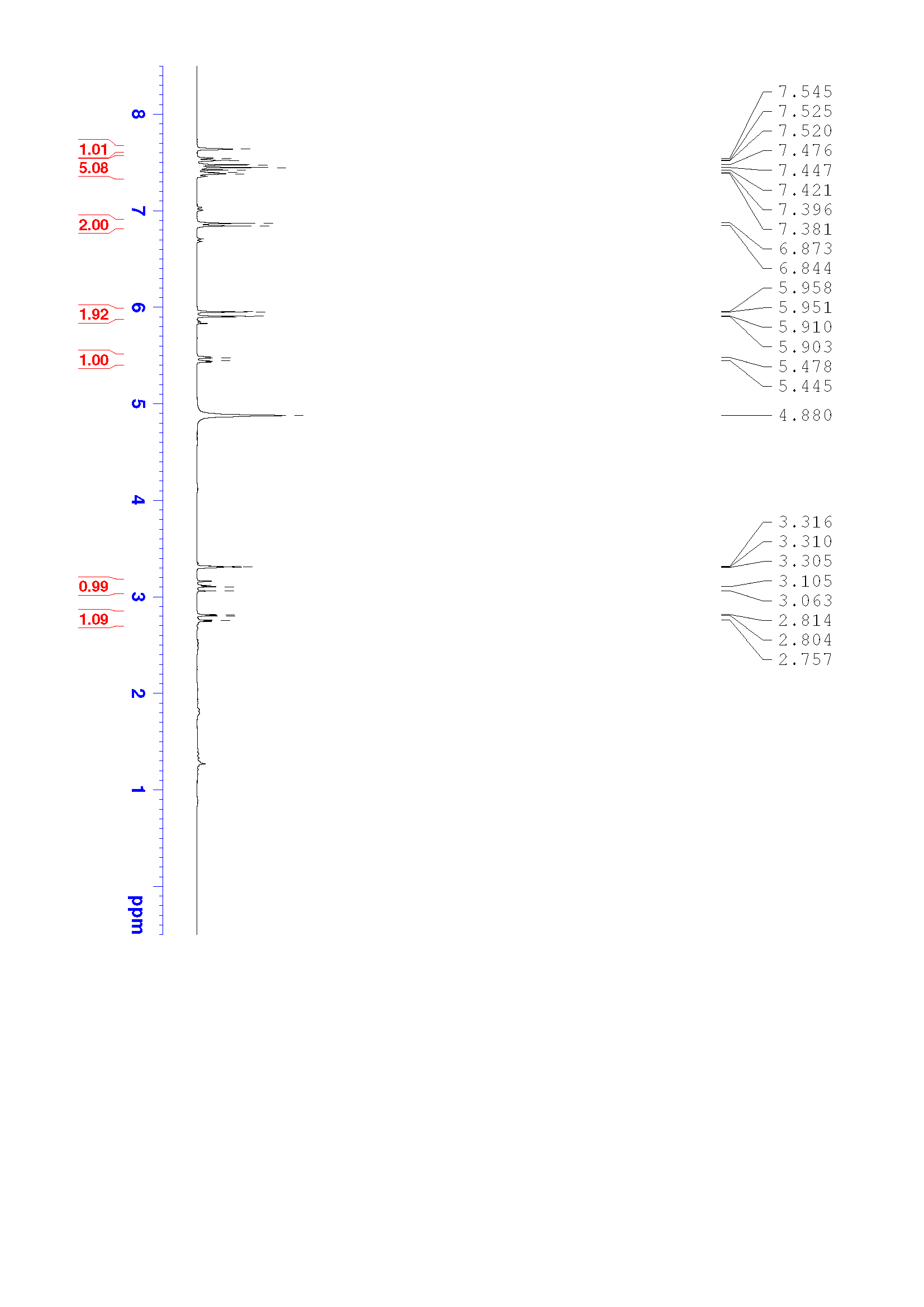


^13^C NMR spectra of compound **11a** measured in CD_3_OD at 75 MHz


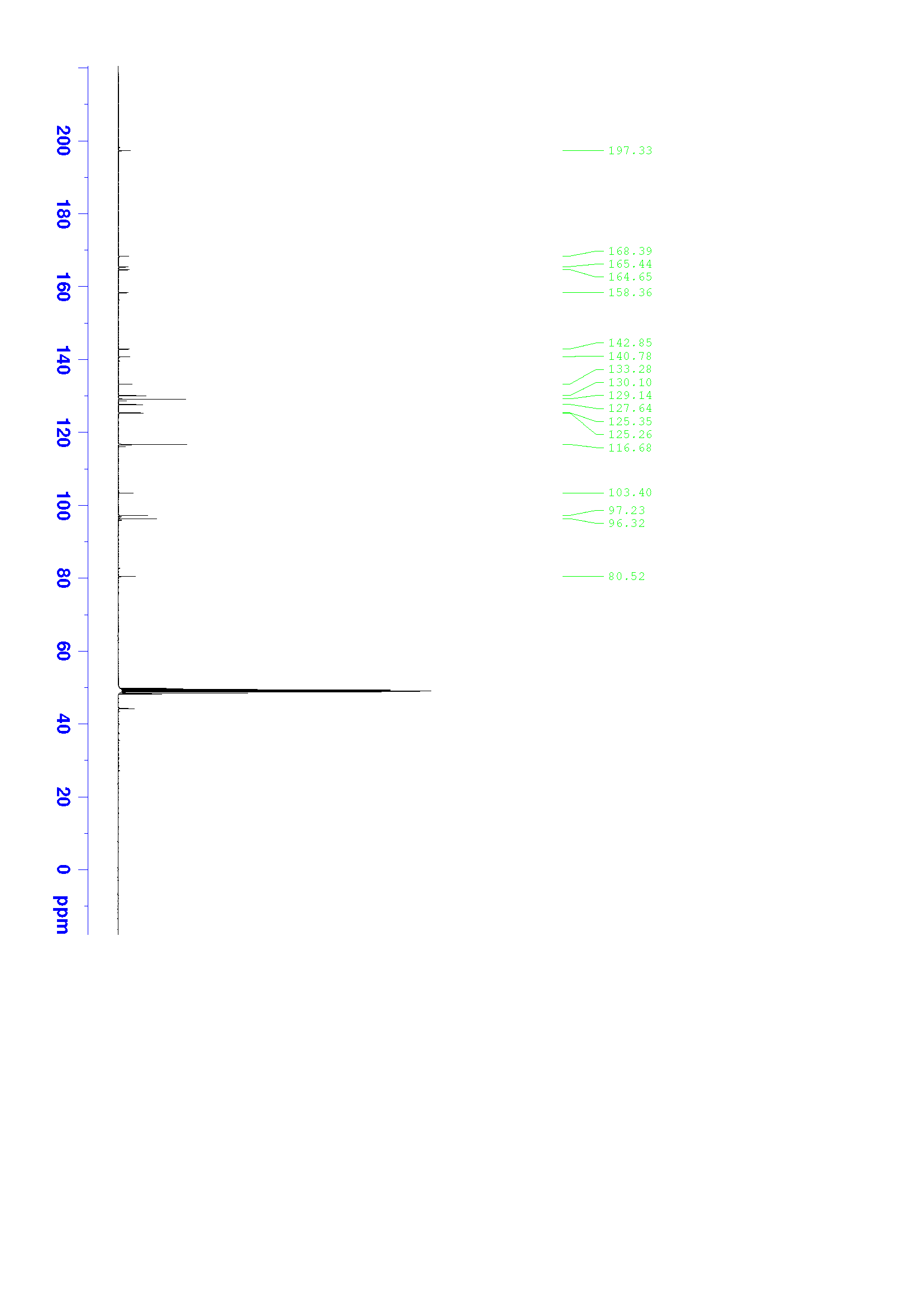


HRMS spectra of compound **11a**

**Figure S1**. Carbocation resonance structure of compound **11a**

**Microscale thermophoresis (MST) experiments**

All experimental preparation was conducted according to the manufacturer’s instructions (NanoTemper Technologies GmbH, Germany). Purified protein, TSLP, was labeled by using Monolith NT^TM^ Protein Labeling Kit RED-NHS 2^nd^ Generation. The labeled protein was adjusted to final concentration of 250nM, approximately. Serial diluents (16 samples) of the compound **1** were prepared with concentrations from 250 μM to 15.26 pM. Binding buffer was 20mM HEPES, pH 7.4, 150mM NaCl with 0.1% N-lauroylsarcosine sodium salt (Sigma-Aldrich, Germany), which was used to prevent surface adsorption of proteins to the capillaries. The labeled protein and serial diluents of compounds were mixed with 1 to 1 ratio to yield the final samples. Monolith NT.115 Premium Capillaries (NanoTemper Technologies GmbH, Germany) were filled with the mixtures, and then, loaded into Monolith NT.115 system for thermophoresis experiment. MO. AFFINITY ANALYSIS Software (NanoTemper Technologies GmbH, Germany) was used to determine the apparent *K_d_*.

**Pull-down assay**

HisPur Ni-NTA resin (Thermo Fisher Scientific Inc.) were equilibrated by 50mM sodium phosphate buffer at pH6.8 with 100mM NaCl. A 400 μL of 200 μM TSLPR with 8×His-tag at the C-terminus was added to the 20 μL of beads and incubated at 4℃ for 1hr, and then, washed by 1 mL of buffer for three times to remove the unbound sample. A 400 μL of 200 μM TSLP with FLAG-tag at the N-terminus was added and incubated with TSLPR bound beads. Unbound TSLP was washed by 1mL of buffer for three times. A series of compound **1** solution with different concentrations (0 to 100 μM) were added and incubated at 4℃ for 1hr. The beads were spun down and washed by 1 mL binding buffers for three times. After elution of the protein samples SDS-PAGE sample buffer, these samples were loaded for gel electrophoresis. TSLPR was stained with Coomassie Brilliant Blue (CBB), and FLAG-tagged TSLP was detected by immunoblotting with Monoclonal ANTI-FLAG® M2-Peroxidase (HRP) antibody (Sigma-Aldrich, Germany).

Figure S2. Western blot (TSLP, upper) and SDS-PAGE (TSLPR, lower) results from the pull-down assay to monitor the dose-dependent inhibition by compound **1**. A series of compound **1** solutions with different concentrations (0, 10, 50, 100μM) were used.


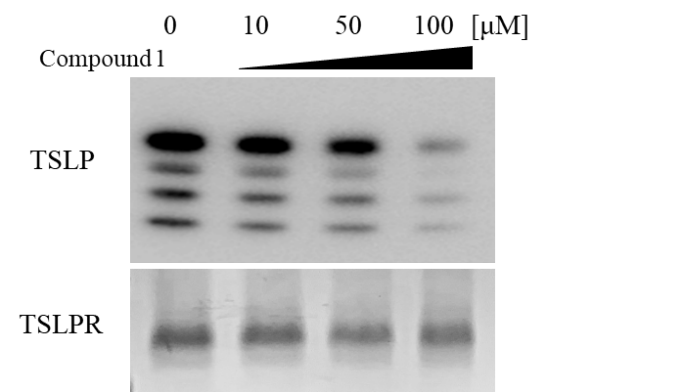


Figure S3. Plot of the bound fraction of compound **1** in the titration of the TSLP solution (250 nM), which gave the *K*_d_ value of compound 1 (27 μM).

Figure S4. Western blot of compounds **10e-10g** compared with compound **1**


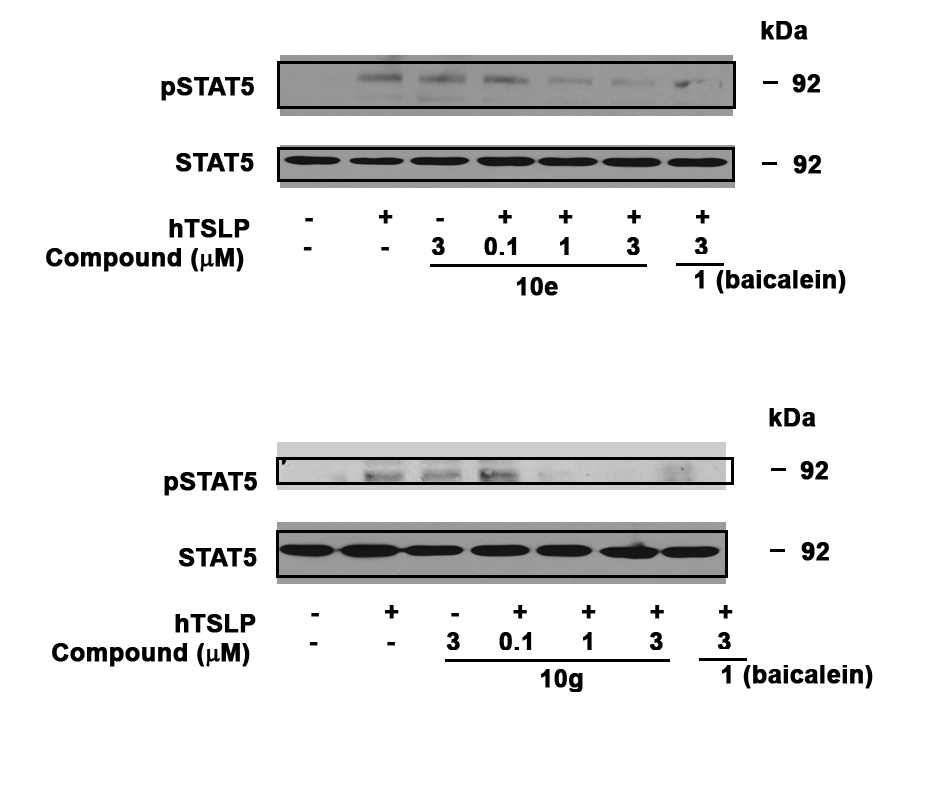


Table 1. STAT5 phosphorylation % of in-house natural products

| Code No. | STAT5 phosphorylation % | | |
| --- | --- | --- | --- |
|  | 3 μM | 10 μM | 30 μM |
| KUP-013 | 93.1 |  | 81.1 |
| KUP-014 | 83.0 |  | 77.4 |
| KUP-026 | 71.8 | 60.5 |  |
| KUP-027 | 70.3 | 66.4 |  |
| KUP-031 | 65.7 | 61.6 |  |
| KUP-037 | 95.1 | 99.9 |  |
| KUP-040 | 69.7 | 68.8 |  |
| KUP-043 | 64.9 | 67.6 |  |
| KUP-076 | 87.9 | 89.9 |  |
| KUP-077 | 94.0 |  | 96.7 |
| KUP-078 | 75.7 | 76.7 |  |
| KUP-079 | 89.4 | 88.6 |  |
| KUP-084 | 95.0 | 90.0 |  |
| KUP-085 | 85.1 |  | 86.5 |
| KUP-086 | 81.4 | 84.2 |  |
| KUP-088 | 111.9 |  | 104.3 |
| KUP-089 | 85.1 | 51.9 | 56.0 |
| KUP-094 | 89.2 | 86.2 |  |
| KUP-102 | 91.2 |  | 76.6 |
| KUP-109 | 76.6 |  | 72.8 |
| KUP-110 | 70.6 |  | 65.5 |
| KUP-116 | 80.8 | 78.5 |  |

Table 2. TSLP-inhibitory activities of in-house natural products by ELISA

| Code No. | % inhibition | |
| --- | --- | --- |
|  | **0.3 mM** | **1 mM** |
| KUP-010 | 78.6 ± 1.3 | 95.7 ± 1.1 |
| KUP-011 | 6.7 ± 1.7 | 32.0 ± 0.3 |
| KUP-014 | 16.7 ± 1.2 | 3.4 ± 0.0 |
| KUP-026 | 8.8 ± 1.1 | 13.9 ± 2.0 |
| KUP-027 | -3.1 ± 1.3 | -0.3 ± 0.9 |
| KUP-031 (Baicalein) | 52.5 ± 2.3 | 77.5 ± 1.6 |
| KUP-034 | 23.8 ± 4.0 | 24.6 ± 1.5 |
| KUP-040 | 12.1 ± 1.8 | 19.9 ± 0.2 |
| KUP-043 | -1.4 ± 1.8 | -1.7 ± 2.5 |
| KUP-056 | 11.0 ± 2.2 | 35.9 ± 1.4 |
| KUP-060 | -0.2 ± 1.4 | 8.6 ± 1.8 |
| KUP-080 | 23.7 ± 2.0 | 34.6± 3.9 |
| KUP-088 | 19.5 ± 1.2 | 24.8 ± 3.5 |
| KUP-089 | 27.3 ± 1.9 | 14.3± 0.9 |
| KUP-109 | 4.2 ± 1.5 | 2.5 ± 2.7 |
| KUP-110 | 20.9 ± 4.0 | 33.7 ± 10.2 |
| KUP-117 | -17.0 ± 1.1 | 4.0 ± 6.8 |
| KUP-118 | 14.0 ± 3.8 | 8.4 ± 5.8 |
| KUP-119 | 1.7 ± 9.0 | 11.6 ± 6.3 |
| KUP-120 | -5.9 ± 5.2 | -21.3 ± 0.2 |
| KUP-121 | -9.1 ± 6.4 | -10.9 ± 0.2 |
| KUP-122 | 12.8 ± 0.6 | 26.3 ± 0.0 |
| KUP-132 | 20.6 ± 2.8 | 35.5 ± 2.6 |
| KUP-137 | -8.6 ± 0.4 | 14.0 ± 0.8 |
| KUP-138 | 26.8 ± 3.0 | 46.5 ± 4.7 |
| KUP-143 | 1.2± 3.3 | 4.0 ± 0.6 |
| KUP-144 | 36.5 ± 3.2 | 83.5 ± 2.8 |
| KUP-145 | -7.0 ± 2.2 | 23.5 ± 1.9 |
| KUP-146 | 3.7± 1.9 | 9.8 ± 4.5 |
| KUP-147 | -3.6 ± 3.1 | -4.3 ± 0.6 |
